# The exceptional stem cell system of *Macrostomum lignano*: Screening for gene expression and studying cell proliferation by hydroxyurea treatment and irradiation

**DOI:** 10.1186/1742-9994-4-9

**Published:** 2007-03-09

**Authors:** Daniela Pfister, Katrien De Mulder, Isabelle Philipp, Georg Kuales, Martina Hrouda, Paul Eichberger, Gaetan Borgonie, Volker Hartenstein, Peter Ladurner

**Affiliations:** 1 Institute of Zoology, University of Innsbruck. Technikerstrasse 25, A-6020 Innsbruck, Austria; 2 Department of Biology, University of Ghent, Ledeganckstraat 35, B-9000 Ghent, Belgium; 3 Department of Radiotherapy and Radiation Oncology, University Hospital Innsbruck, Medical University Innsbruck. Anichstrasse 35, A-6020 Innsbruck, Austria; 4 Department of Molecular, Cell and Developmental Biology, University of California Los Angeles, Los Angeles, CA 90095, USA

## Abstract

**Background:**

Flatworms are characterized by an outstanding stem cell system. These stem cells (neoblasts) can give rise to all cell types including germ cells and power the exceptional regenerative capacity of many flatworm species. *Macrostomum lignano *is an emerging model system to study stem cell biology of flatworms. It is complementary to the well-studied planarians because of its small size, transparency, simple culture maintenance, the basal taxonomic position and its less derived embryogenesis that is more closely related to spiralians. The development of cell-, tissue- and organ specific markers is necessary to further characterize the differentiation potential of flatworm stem cells. Large scale in situ hybridization is a suitable tool to identify possible markers. Distinguished genes identified in a large scale screen in combination with manipulation of neoblasts by hydroxyurea or irradiation will advance our understanding of differentiation and regulation of the flatworm stem cell system.

**Results:**

We have set up a protocol for high throughput large scale whole mount in situ hybridization for the flatworm *Macrostomum lignano*. In the pilot screen, a number of cell-, tissue- or organ specific expression patterns were identified. We have selected two stem cell- and germ cell related genes – *macvasa *and *macpiwi *– and studied effects of hydroxyurea (HU) treatment or irradiation on gene expression. In addition, we have followed cell proliferation using a mitosis marker and bromodeoxyuridine labeling of S-phase cells after various periods of HU exposure or different irradiation levels. HU mediated depletion of cell proliferation and HU induced reduction of gene expression was used to generate a cDNA library by suppressive subtractive hybridization. 147 differentially expressed genes were sequenced and assigned to different categories.

**Conclusion:**

We show that *Macrostomum lignano *is a suitable organism to perform high throughput large scale whole mount in situ hybridization. Genes identified in such screens – together with BrdU/H3 labeling – can be used to obtain information on flatworm neoblasts.

## Background

Platyhelminthes possess an extraordinary stem cell system. A single cell type – pluripotent stem cells (neoblasts) – is responsible for cell renewal during growth, development, homeostasis and regeneration. Neoblasts comprise a pool of undifferentiated cells in the parenchyma [1-10]. Typically, they are characterized by their small size (6–10 μm), a high nuclear/cytoplasmic ratio, and they feature a large nucleus with a prominent nucleolus, a thin rim of cytoplasm with free ribosomes and few mitochondria and no or very little endoplasmic reticulum [2,7,11-14]. Also in parasitic platyhelminths, cells similar to neoblasts have been described [15-20].

We have applied bromodeoxyuridine labeling to show the distribution, migration and differentiation of S-phase neoblasts in *Macrostomum lignano *(Macrostomida, Platyhelminthes) [4,8,21] and a similar BrdU study was applied in planarian flatworms [9]. For example, in rhabditophoran flatworms, a large taxon that includes free-living forms such as e.g. macrostomid and polyclad flatworms, the planarians and all parasitic groups, the epidermis does not posses a proliferative compartment but is renewed from mesodermally located neoblasts. Most strikingly, germ cells are regenerated from neoblasts of small tissue pieces devoid of any germ line cells [22]. For *Macrostomum lignano*, restoration of germ line cells from tissue lacking gonads has been observed after 29 or 45 successive amputations [23] (Egger unpublished) of the same individual animals.

In recent years, a number of specific stem cell- and germ cell markers have been identified for different planarian species to study the distribution of neoblasts and to follow the fate of neoblast during regeneration. [10,24-29,31-34]. Recent reviews summarize the progress in understanding flatworm stem cells and germ cells in intact animals and during regeneration [22,35-39].

In order to obtain information on the neoblast cell dynamics in *M. lignano*, Hydroxyurea (HU) was applied to block neoblast proliferation [4,21]. Hydroxyurea is a DNA synthesis inhibitor. It arrests the cell cycle in early S-Phase by inhibiting the enzyme ribonucleotide reductase [40]. The initiation of DNA replication is not affected. HU starves the DNA polymerase at the replication forks for dNTPs [41]. In *M. lignano*, differences in the sensitivity of somatic stem cells and germ cell proliferation to HU exposure were observed [4]. Furthermore, HU treatment was used to characterize the differentiation time of specific cells by determining the effects on the expression level of a *wnt *antagonist (Hrouda unpublished). The pool of precursor cells for specific differentiated cells is limited by HU treatment. Another widely used method to eliminate proliferating cells is irradiation. The reduction of gene expression after irradiation has been shown for different planarian species and various stem cell- and germ cell related genes [24,28-30,42].

In this study we have applied large scale whole mount in situ hybridization and identified several cell- and tissue specific markers for *M. lignano*. Two genes – a *vasa*-like and a *piwi*-like gene – were selected for further studies. First, the effect of HU treatment on the expression level of the germ cell specific *vasa*-like gene *macvasa *was followed during prolonged periods of HU treatment. Second, the effect of different intensities of irradiation on the expression of the stem- and germ cell marker *macpiwi *was monitored. In addition, we observed that irradiation induced a reduction of cell proliferation, using BrdU to label S-phase cells and an anti-phosphorylated H3 antibody to stain mitoses. In summary, our results show that we can use genes identified in a large-scale whole mount in situ hybridization screen in combination with cell proliferation studies to obtain information on stem cells and germ cells in the platyhelminth *M. lignano*.

## Results

In this study we tried to achieve two goals: (1) to give the proof of principle for *Macrostomum *as a suitable organism for large scale in situ screening. (2) to use selected genes for studying the reduction of cell proliferation by hydroxyurea treatment or irradiation.

We have established a protocol to perform large-scale whole mount *in situ *hybridization for the flatworm *Macrostomum lignano*. The screen revealed a variety of genes showing characteristic expression patterns. Based on this pilot study it will be possible to perform a high throughput large scale whole mount in situ hybridization screen of *Macrostomum *ESTs in the future.

From the current screening, two genes, a v*asa*- and a *piwi*-like gene, were selected to study the effects of hydroxyurea (HU) treatment or gamma irradiation on stem cells and germ cells. Little is known about the cell types, morphology and the process of spermatogenesis in basal flatworms. Therefore knowledge about expression of germ cell specific genes was very much wanting. *Macvasa *and *macpiwi *can be used as stem cell and germ line markers. By e.g. food deprivation, chemical treatment – such as HU – or irradiation we will be able to contribute to the understanding of germ line development in basal flatworms. Detailed information on the *macvasa *and the *macpiwi *gene and expression during postembryonic development and regeneration will be published elsewhere. In addition to gene expression experiments, BrdU labeling was applied to confirm the reduction of cell proliferation induced by HU or irradiation. Furthermore, a library enriched for proliferation or germ cell related genes was generated by suppressive subtractive hybridization of HU treated animals.

### Large-scale whole mount in situ hybridization

**Figure 1:**
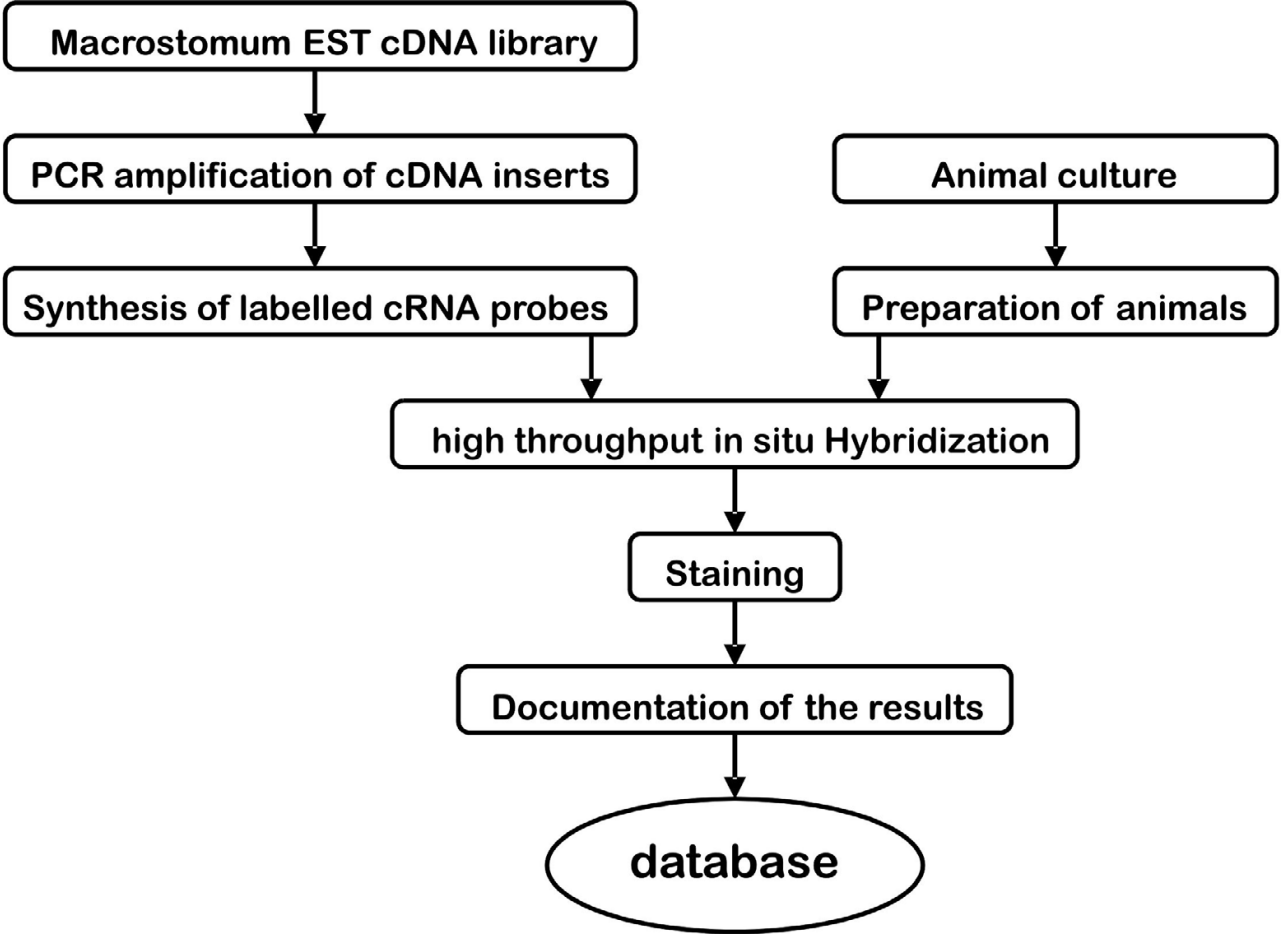
**Methods flowchart**. From ESTs to expression of stem cell and germ line related genes in the flatworm *Macrostomum lignano*.

**Figure 2:**
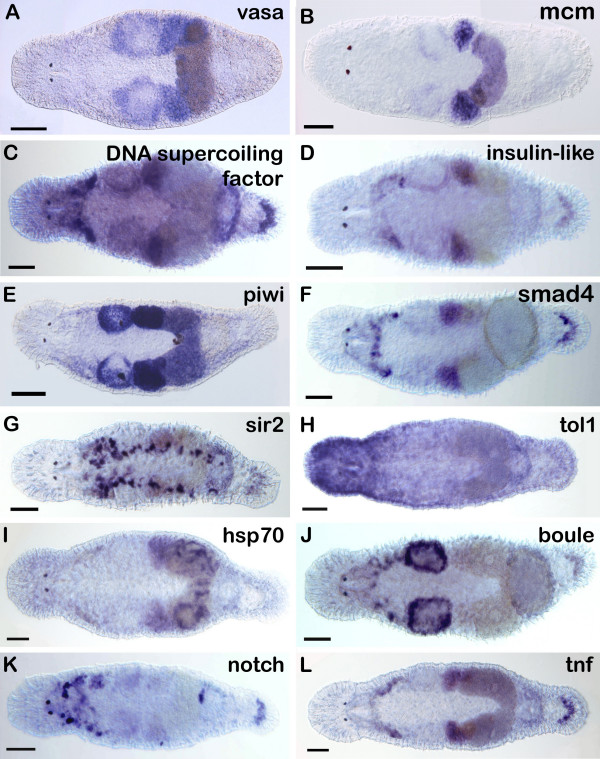
In situ hybridizations in *M. lignano *with selected genes performed with HTI Biolane robot.

We have initiated a pilot study on large-scale whole mount in situ hybridization of *Macrostomum lignano*. We established an efficient strategy for screening a large number of genes for their expression pattern by whole-mount in situ hybridization in adult *M. lignano *(Fig. 1). Our goal was to identify stem cell and germ cell marker genes to analyze their spatial and temporal expression patterns and to study their function. For the current pilot study we have selected 96 genes based on annotations of the *M. lignano *EST project [43] [see Additional file 1]. Genes were selected from different categories [44] like e.g. RNA-binding, cell cycle, transcription factors, chromosome/nuclear structure, growth factors. We have upscaled processing of probe synthesis and converted necessary steps of the in situ hybridization protocol onto the BioLane HTI robot. A number of genes showed cell- and tissue-specific expression patterns (Fig. 2). For example, expression in the gonads was observed for *vasa-*, *MCM*-, *insulin*-, *piwi*-, *smad4*-, *hsp70*-, *boule*- and *tnf*-like genes (2A, B, D, E, I, J, L). Most of these genes were expressed in both gonads, the testes and the ovaries, except for two genes. *Boule *was expressed mainly in the male gonads (2J) and a complementary pattern could be observed with *hsp70*, which expression was restricted to the female gonads (2I). *Piwi *was expressed at a basic level in the stem cells in addition to the expression in both gonads (2E). Specific cell types were stained by *sir2*- and *notch*-like in situ hybridization (2G, K). The distribution of *sir2*-like transcripts was in the gut region, as well as in the pharyngeal glands (2G) and *notch*-like transcripts were found in the pharynx-region (2K). *Tol1 *showed an expression pattern biased to the anterior region of the animal, with strong expression in the head (2H). A subset of cells was labeled by *DNA supercoiling factor*-, *smad4*-, *notch*- and *tnf*-like riboprobes in the tail plate (2C, F, K, L).

### Effects of Hydroxyurea treatment

Hydroxyurea (HU) inhibits DNA-Synthesis and arrests cells in early S-Phase. From literature it is known that HU primarily affects spermatogenesis [45]. Our results corroborate these findings for *Macrostomum*. We observed a decrease of *macvasa *expression in the testes of HU-treated animals. Currently, it is not clear whether spermatogonia, spermatocytes or spermatides are affected specifically. However, by means of histological sections of *macvasa *in situ hybridizations it will be possible to further address this question.

**Figure 3:**
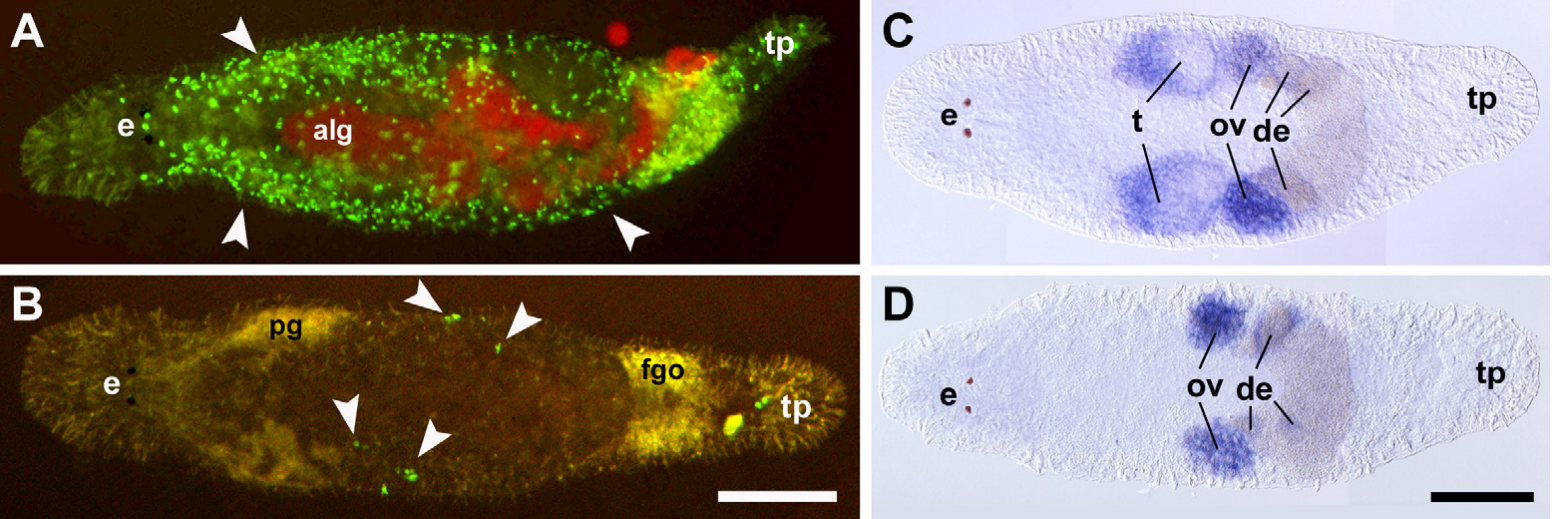
**Treatment of *Macrostomum lignano *with Hydroxyurea**. (A) Non-treated control animal with BrdU-labeled S-phase cells arranged in two lateral bands (arrowheads). (e) alg remnants of digested food algae showing red autofluorescence, tp tail plate. (B) BrdU stainings of *M. lignano *treated with 2.5 mM Hydroxyurea for 8.5 days. Specimen shows about 10 BrdU-labelled cells in S-phase (arrowheads). Some background can be seen in the pharynx glands (pg) and the female genital opening (fgo). tp tail plate. scalebar 100 μm. (C) In situ hybridization of control animal with *macvasa*. Signal present in male (t testes) and female gonads (ov ovaries) and developing eggs (de). e eyes, tp tail plate. (D) In situ hybridization with *macvasa *after 11 days of HU-treatment. Only female gonads (ov ovaries) show signal. Male gonads (t testes) show no expression of *macvasa*. e eyes, de developing eggs, ov ovaries, t testes, scalebar 100 μm.

Here we demonstrate that the *vasa*-like gene *macvasa *was expressed in testes and ovaries of adult *M. lignano *in control animals (Fig. 3C). In order to look at *macvasa *expression after HU-treatment of *M. lignano*, in situ hybridizations were performed. HU-treated animals do not show *macvasa *expression in the male gonads (Fig. 3D). In ovaries, however, the level of expression appeared only slightly reduced or unchanged compared to control animals (Fig. 3D).

In addition to in situ hybridization experiments the effects of HU-treatment on proliferating cells were examined by labeling S-phase cells with BrdU. The distribution of S-phase cells in control animals showed two bands of proliferating cells along the lateral sides and clusters of S-phase cells within the testes (Fig. 3A, see also [8]). After 48 hours of 2.5 mM HU incubation no significant differences between the control- and the treated group were detected. After 3.5 days about 50% of the S-phase cells disappeared and after 5.5 days BrdU-labeled cells could only be seen in the gonads. After 8.5 days and 10 days only about 10–40 S-phase cells remained stained in the reproductive organs (Figure 3B).

### Influence of irradiation on the stem cell population

**Figure 4:**
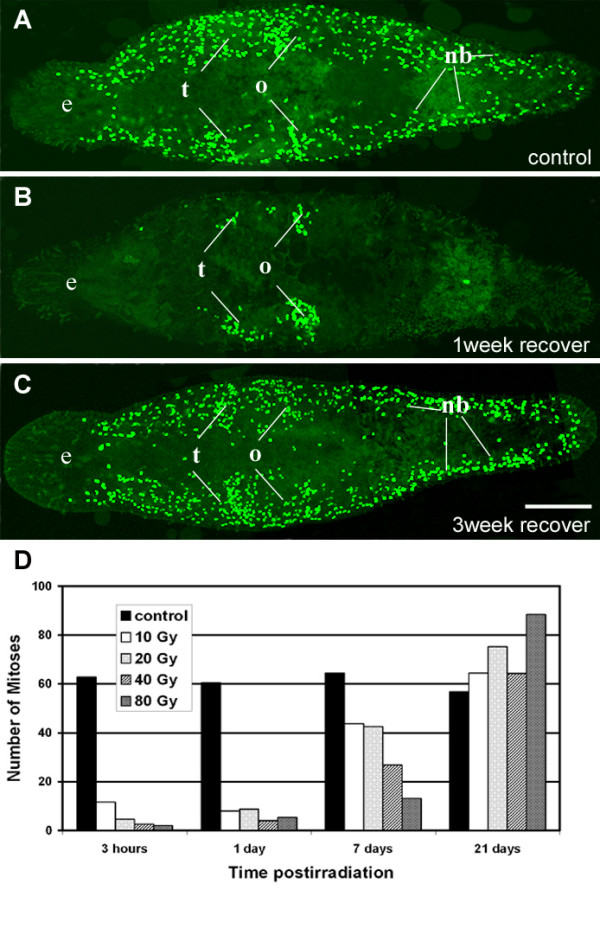
**Influence of irradiation on the stem cell population as shown by BrdU-incorporation in *M. lignano***. (A) Confocal projection of labeled S-phase cells in non treated control animal after 30 minutes BrdU-pulse. Note bilaterial distribution of BrdU cells and clusters in testes (t) and ovaries (o). (B) Confocal projection of labeled S-phase cells after one week recovering from 80 Gy irradiation. BrdU labeled cells can be found only in gonads. (C) Confocal projection after 3 weeks recovering from irradiation, BrdU incorporation was comparable with control. (D) Effect of irradiation on mitoses. Note the dramatic reduction in the number of mitoses 3 hours and 1 day postirradiation and the recovery of cell proliferation after 7 and 21 days. scalebar 100 μm.

**Figure 5:**
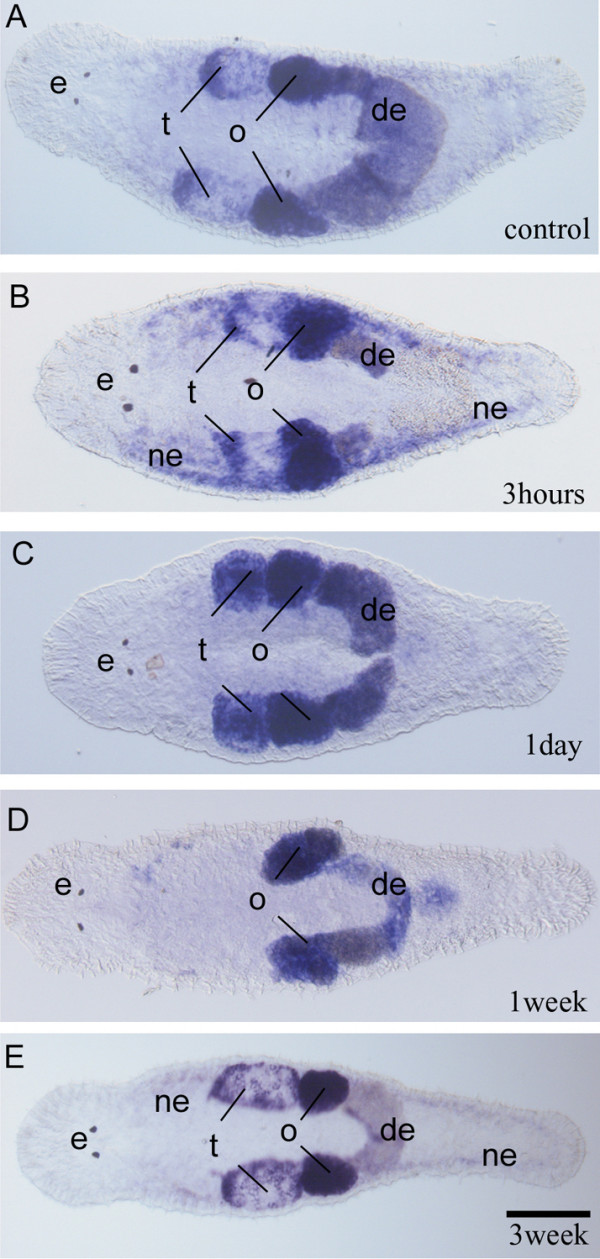
**Influence of irradiation on the stem cell population as shown by *macpiwi *expression in *Macrostomum***. (A) *Macpiwi *expression in control adult. Signal was present in testes (t), ovaries (o), developing eggs (de) as well as at a basic level in somatic stem cells. (B) Expression of *macpiwi *in somatic stem cells 3 hours postirradiation. (C) After 1 day recovering from irradiation, *macpiwi *expression in somatic stem cells is lacking, but present in the gonads. (D) *Macpiwi *expression 1 week postirradiation (80 Gray). Note the complete loss of *macpiwi *expression in testis. (E) *Macpiwi *expression resembles default expression pattern 3 weeks postirradiation (80 Gray). scalebar 100 μm.

We have irradiated *M. lignano *with doses of 10, 20, 40, and 80 Gray, respectively. Effects on cell proliferation were examined by BrdU/phos-H3 stainings and *macpiwi *gene [see Additional file 2] expression after 3 hours, 1 day, 7 days and 21 days (Fig. 4, 5).

The number of mitoses was decreased significantly 3 hours after irradiation and remained at a low level at 1 day postirradiation (Fig. 4D). Cell proliferation has partially recovered one week after irradiation but reflected a correlation to the irradiation levels. Low levels of irradiation (10 and 20 Gray) picked up to about 70% of the number of mitoses compared to control animals while higher doses (40 and 80 Gray) got back to less than 30%, respectively. After 3 weeks postirradiation the number of mitoses at all irradiation doses was even higher than the number of mitotic cells of control animals (Fig. 4D). We could not collect data on the number of S-phase cells 3 h and 24 h postirradiation. However, earlier experiments revealed that cell proliferation of somatic stem cells was strongly reduced after 2 h and 24 h at irradiation levels of 25, 50, 100, 150, and 200 Gray (Ladurner unpublished). In the current experiment, we observed differences in the number of S-phase cells between lower doses and higher doses of irradiation after 1 week. At lower doses (10, 20 Gy) the number of S-phase cells was comparable with control animals. In contrary, at higher doses (40, 80 Gy) BrdU incorporation was only detected in gonadal cells (Fig. 4B). Three weeks postirradiation animals recovered completely and the pattern of BrdU incorporation corresponded to the distribution of BrdU labeled cells of control animals (compare Fig. 4A,C).

The *piwi *homologue *macpiwi *was expressed in both testis and ovary, as well as in somatic stem cells (Fig. 5A). *Macpiwi *expression was still present in somatic neoblasts and gonadal cells 3 h post irradiation (Fig. 5B). After 1 day recovering, *macpiwi *expression was observed in the gonads, but lacking in somatic stem cells (Fig. 5C). Comparable to cell proliferation results, the influence of the irradiation dose (10 or 20 Gy versus 40 or 80 Gy) became apparent 1 week postirradiation. Animals irradiated with 10 or 20 Gy do not show an alteration in *macpiwi *expression compared to control animals (data not shown). However, worms treated with 40 or 80 Gy, have lost *macpiwi *expression in testis and somatic stem cells (Fig. 5D). After 3 weeks postirradiation, *macpiwi *was expressed comparable to control animals in testes, ovaries and in somatic stem cells (Fig. 5E).

### Subtraction library

**Figure 6:**
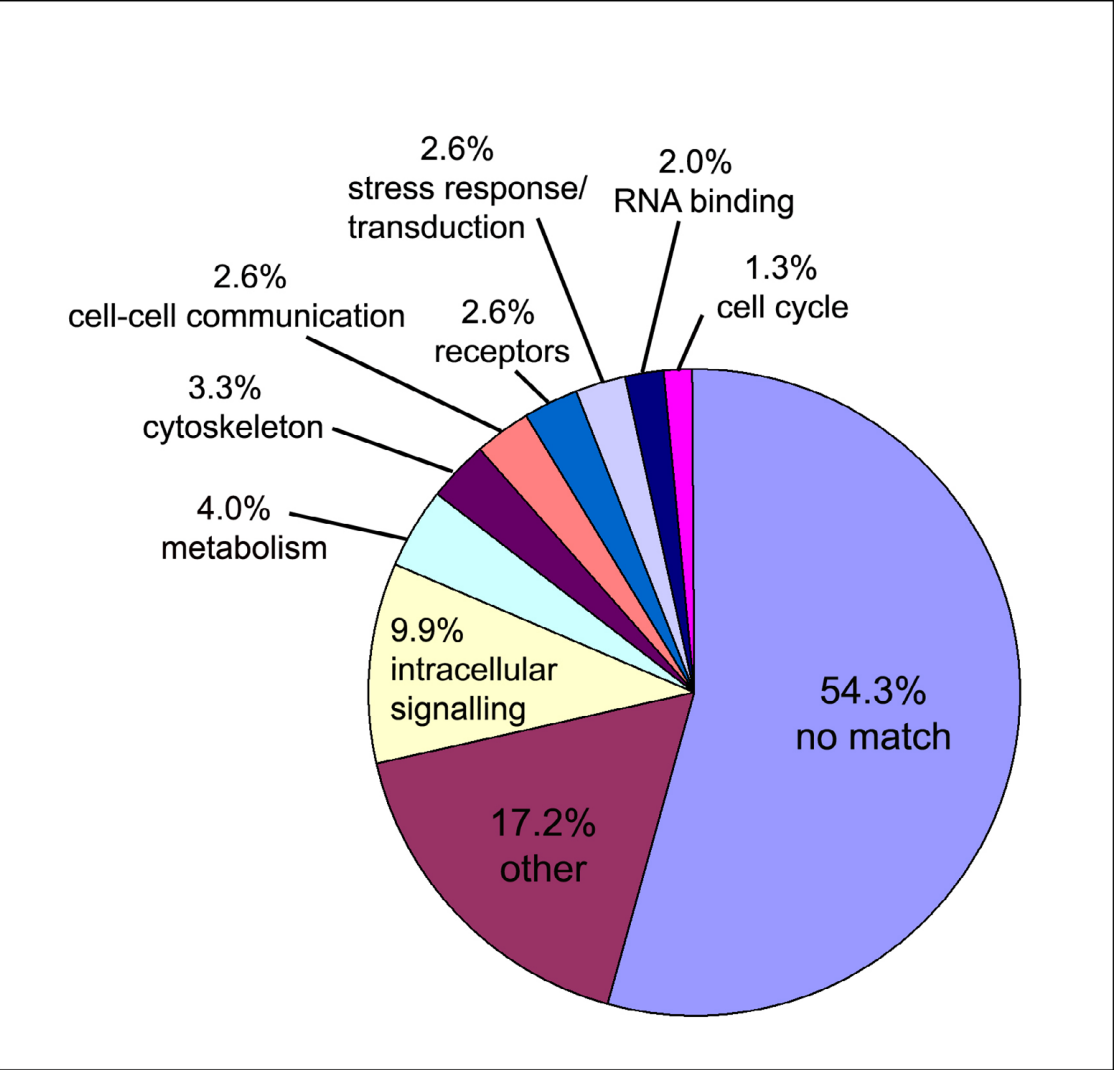
**Groups of differentially expressed genes of a subtraction library of Hydroxyurea-treated *M. lignano***. The pie chart shows 10 categories of differentially expressed and sequenced genes (n = 147) that were used for BLASTx analysis (Expect value ≤ 1 × 10^-3^). See text for example of genes of each category.

In order to identify differentially expressed genes specific for stem cells and germ line cells, a cDNA subtraction library was produced. cDNA of HU treated animals was subtracted from control animals to enrich for respective genes. The differential screening yielded 148 clones. Of these 147 were sequenced and used for BLAST analysis. BLAST hits were assigned to 10 gene categories in terms of their associated biological processes, cellular components and molecular functions similar to the *M. lignano *EST project [43] and are depicted in a pie chart according to their percentage (Figure 6). A list of the top ten BLAST results for all clones with E values ≤ 1 × 10^-2 ^is shown in a table [see Additional file 3]. In the category "RNA binding" e.g. SNRPF protein (similar to *Mus musculus *SNRPF) could be identified (E value of 6e-26). The gene codes for small nuclear ribonucleoprotein polypeptide F that belongs to the snrnp sm proteins family. The molecular function is RNA binding, RNA splicing factor activity and a transesterification mechanism. It is involved in mRNA processing, nuclear mRNA splicing via spliceosome and located in the nucleus. In the category "Intracellular signalling" an Arginine kinase (E-value of 2e-06) was identified, as well as a protein phosphatase Y (Serine/threonine specific protein phosphatase, *Drosophila*, E value of 4e-09), a sperm cAMP-dependent protein kinase catalytic subunit (E value of 2e-35), casein kinase I (E value of 2e-17) and several phosphatases. In the "transduction" category a FMRF-amide-gated Na-channel (E value of 1e-04) and a CocoaCrisp Protein (Rattus norvegicus, E value of 5e-04), which belongs to a family of extracellular domains (Human glioma pathogenesis-related protein GliPR). The category "other" includes a serine protease that is newborn-larvae specific (*Trichinella spiralis*, E value of 2e-04). In the "receptor" category a kremen protein 1 precursor (E value 5e-4) and Kremen2 (*Xenopus laevis*, E value 2e-4) are included. In "Metabolism" a putative tubulin-tyrosine ligase (E value 2e-4) and a cysteine sulfinic acid decarboxylase (E value of 4e-19) was found.

## Discussion

### Effect of inhibition of cell proliferation by Hydroxyurea

HU treatment of *M. lignano *lead to a substantial reduction in the number of S-phase cells after 3.5 days of HU incubation. At 8.5 days and 10 days HU exposure, somatic neoblasts did not show BrdU incorporation. In the gonads, however, a small number of BrdU labeled cells were present. Moreover, expression of the germ line marker *macvasa *was restricted to the ovaries and developing eggs. Testes were completely devoid of *macvasa *expression. A similar phenomenon was observed in mice. There, spermatogonia and spermatocytes were found to be affected selectively by HU [45]. In vivo HU exposure induced testicular germ cell apoptosis and damaged cells were eliminated by phagocytosis by neighbouring cells. In Cnidarians, HU has an effect on nerve cells. The production of nerveless *Hydra attenuata *by hydroxyurea treatments was shown by Sacks and Davis [46].

For *Macrostomum lignano*, two groups of animals – HU treated and controls – were used to generate a subtraction library enriched for differentially expressed genes specific for stem cells and germ line cells. Suppressive Subtractive hybridization was also used to e.g. isolate differentially expressed genes involved in interactions between the snail host and the parasitic flatworm *Schistosoma mansoni*, for investigating mechanisms of wound healing [47] or the identification of progenitor-cell-specific genes [48].

### Large scale whole mount in situ hybridization

We have demonstrated that *M. lignano *can be used for high throughput large scale whole mount in situ hybridization. With the development of high-throughput robot systems large-scale in situ hybridization, screens have been performed in several organisms such as e.g. *Drosophila*, *Ciona*, *Xenopus*, *Schmidtea*, *Danio*, *Medaka and Mus *[49-57]. In these screens, the high potential of large scale in situ hybridization for the discovery of new genes has been shown. A large number of cell-, tissue- and organ specific markers were identified. A variety of valuable molecular markers of developmental studies became available from these screens. In *M. lignano*, we could identify gonad-specific genes such as *insulin-*, *smad4*-, *tnf-, MCM- *and *hsp70-like*, which were predominantly expressed in the ovaries. On the contrary, a *boule-like *gene was expressed only in the testes and with *vasa *and *piwi*, we found markers that are present in both gonads of the simultaneous hermaphrodite *M. lignano*. These genes will prove to be useful markers for experiments dealing with sex allocation or mating experiments.

The expression patterns of *sir2 *and *insulin-like *qualify these genes as candidates for studying aging. Riboprobes of four other genes, *DNA supercoiling factor-, smad4-, notch-, tnf-like *probably stain adhesive organ cell bodies. These findings may be used for differentiation and regeneration studies. With high throughput large scale whole mount in situ hybridization, genes with expression patterns interesting for different research groups become readily available and can be cloned and sequenced from the respective cDNA library.

### Effect of inhibition of cell proliferation by irradiation

Irradiation of *M. lignano *with different doses of gamma rays has shown that an irradiation sensitive population of proliferative cells exist in this animal. Few hours postirradiation, the somatic compartment of S-phase and mitotic cells has been strongly reduced while cell proliferation was still present in the gonads. After one week, BrdU incorporation was comparable to control animals irradiated with 10 or 20 Gray while mitoses were reduced to 70%. Higher doses (40 and 80 Gy) resulted in a lack of somatic stem cell proliferation while S-phase cells were visible in the gonads. At three weeks postirradiation animals of all irradiation levels showed normal S-phase cell distribution pattern. In contrast, irradiation of different planarian species with 30 Gy always resulted in a complete absence of proliferating stem cells [24,28,30,58,59]. In *M. lignano*, after 3 h postirradiation *macpiwi *was still expressed in mesenchymal and gonadal cells. In cancer cells, gene expression profiling revealed an increase of expression of genes involved in e.g. in DNA repair [60]. *Piwi*-like genes are not considered as repair genes but play a role in regulation of stem cells and germ cells. This molecular function of *piwi*-like genes could result in the presence or absence of expression after irradiation. At one week postirradiation, *macpiwi *expression completely disappeared in the testes and somatic stem cells of worms irradiated with 40 or 80 Gy. Interestingly, in mice, the expression of e.g. growth factor genes was decreased specifically in testicular germ cells while somatic cells (Sertoli) maintained expression.

The recovery of cell proliferation in *Macrostomum *could have different explanations: First, somatic stem cells entered cell cycle arrest upon irradiation but were able to re-enter the cell cycle after 2–3 weeks of recovery. Second, non cycling neoblasts became activated and re-established the neoblast pool. A number of non cycling neoblasts have been observed in *M. lignano *[61]. Third, gonadal stem cells recover the somatic stem cell population. An entry of cells from the ovary has been observed to contribute to the somatic stem cell population during regeneration in planarians [62]. Fourth, methodological considerations have to be taken into account. It might be possible that differences in the application of the respective irradiation levels in *M. lignano *and in planarians are not equal despite the fact that the same doses, i.e. the number of Gray, were given. The actual "effective dose" applied could account for observed differences in the sensitivity to irradiation of macrostomid and planarian taxa. A complete elimination of S-phase cells in *M. lignano *was not achieved while planarians required transplantation enriched neoblasts of donor to be rescued from 30 Gy irradiation dose [59]. Moreover, *Macrostomum *irradiated with 40 or 80 Gy regenerated and remained alive while planarians disintegrated.

### Subtraction library

BLAST analyses of the 147 obtained differentially expressed clones revealed a majority of sequences that did not show any match with BLAST databases (54.3%, "no match"). This group may contain some novel stem cell and germ line specific genes in *M. lignano*, in particular genes that are connected with testis-specific expression pattern. Surprisingly, we have not found genes homologous to known stem cells or proliferation genes despite the dramatic reduction of cells in S-phase after HU treatment (Figs. 3A,B). Most likely the majority of mRNA was not reduced substantially after HU incubation resulting in a low representation of such genes in the SSH library.

## Conclusion

The tissue architecture of flatworms and the mode of cell turnover during development, homeostasis and regeneration is unique within the animal kingdom. All cells, including the germ line, are derived from totipotent somatic cells called neoblasts. The fact that the molecular regulation of stem cells and the germ line appears to be conserved between species and phyla, makes flatworms suitable organisms to study questions related with stem cell biology. *Macrostomum lignano *is a small worm (1 mm), highly transparent, easy to culture and has a simple tissue organisation. Substantial background on morphology, development and regeneration are available and cell- and molecular biology tools have been worked out, especially RNA interference to knock down genes can be applied by injection or soaking (Pfister, De Mulder, unpublished data). The current study shows that *M. lignano *can be used for high throughput analysis to identify cell-, tissue- or organ specific markers. In addition, we prove that it is possible to manipulate the *M. lignano *stem cell system with hydroxyurea or irradiation and combine it with the study of cell proliferation and gene expression. In summary, an extended screen for genes will result in a capacious assortment of markers and can open new avenues to study development, regeneration and other research fields.

## Methods

### cRNA probe synthesis for large scale *in situ *hybridizations

The *Macrostomum *EST library was cloned directedly into Sport 6.1 vectors (Invitrogen) with T7 for antisense transcription [43]. PCRs with M13 standard primers were performed to generate templates for RNA probe synthesis. PCR conditions were 5 minutes at 95°C, 35 cycles (95°C for 45 s, 55°C for 45 s, 72°C for 1 min 20 s), 72°C for 2 minutes. Riboprobes were synthesized with the RNA in vitro transcription master mix (final volume of 10 μl per well) using the T7 transcription polymerase from Promega. Transcription was performed after manufacturer's instructions except for the use of DIG-labeling mixture from Roche. 1 μl of each transcription was checked by gel electrophoresis and 9 μl transcription reaction were diluted in 500 μl HybMix and used directly for the *in situ *hybridization screen.

### *In situ *hybridization screening with the HTI Biolane robot

For the pilot screening, batches of ~1000 animals were pretreated for the prehybridization similar to the whole mount *in situ *protocol with the following exceptions: both de- and re-hydration series were always performed in methanol. For hybridization, about 20 animals each were distributed into the mesh vessels of the robot system (HTI Biolane, Hölle and Hütter AG). Mesh vessels were placed into plastic vials. Pre-hybridization was perfomed in 100% HybMix for 2 h at 55°C (identical to the standard *Macrostomum in situ *hybridization protocol below). For the screen, the whole transcription reaction was diluted in 500 μl HybMix and used. For hybridization, the samples were re-incubated at 55°C for 1.5 days with the riboprobes. The stringent washes with SSC mixtures were performed according to *Macrostomum *standard protocol. Vials were placed first into the robot rack, which then was placed into the robot plastic container. Washes were then performed in a 55°C oven outside the robot and media were exchanged manually. Starting from the MAB washes the *in situ *post washes were performed within the robot. Color development was performed manually with NBT/BCIP in 24-well plates at 37°C within 15 minutes. This was followed by ethanol and PBS washes, as well as embedding and photographing of the samples according to the standard protocol.

### In situ hybridization protocol for *M. lignano *with *macvasa or macpiwi*

Animals were relaxed in 1:1 7,14% MgCl2:artificial sea water (ASW) for 5 minutes and 10 minutes in pure 7,14% MgCl2. Fixation was done in 4% PFA in 0.1 M PBS for one hour at room temperature. Fixative was removed with three 5-minutes 1× PBS-Tween (0.1%) washes, and animals were dehydrated by ascending Methanol series and stored at -20°C. Whole mount in situ hybridization was performed using a newly developed protocol for *M. lignano *[63] based on a modified in situ protocol for *Hydra *[64,65]. Animals were rehydrated by an Ethanol series (100 % Ethanol for 5 × 5 minutes, 75%, 50% and 25% Ethanol with DEPC-treated H_2_O for 5 minutes each) followed by three washes with 1× PBS-Tween. Proteinase-K treatment (20 μg/ml) was done at room temperature for 17 minutes and stopped with Glycine (4 mg/ml in 1× PBS-Tween). Animals were incubated 3 × 5 minutes in 1× PBS-Tween, 2 × 5 minutes in 0.1 M TEA, 2 × 5 minutes in 0.1 M TEA with acetic anhydride (400:1), 2 × 5 minutes in 0.1 M TEA with acetic anhydride (200:1) and 2 × 5 minutes in 1× PBS-Tween. Animals were refixed in 4% PFA in 0.1 M PBS for 20 minutes at room temperature followed by 5 × 5 minutes 1× PBS-Tween washes. Animals were heat-fixed at 80°C for 20 minutes, shaking. Subsequently an incubation in 1× PBS-Tween/HybMix (1:1) for 10 minutes at room temperature and in 100% HybMix (50% formamide, 5× SSC, 100 μg/ml heparin, 0.1% Tween, 0.1% CHAPS, 200 μg/ml yeast tRNA, 1× Denhardt's) for 10 minutes at 55°C was performed. Animals were prehybridized in fresh HybMix at 55°C for 2 hours. Riboprobe was added (working concentration 0.075 ng/μl for *macvasa *and 0.1 ng/ml for *macpiwi*) after denaturation (7 minutes at 95°C and snap chilled on ice). Hybridization time was 2.5 days at 55°C for *macvasa *and 1.5 days at 55°C for *macpiwi*, shaking continuously at 300 rpm. Stringent washes at 62°C replaced HybMix subsequently with 2× SSC, followed by two washes with 2× SSC/0.1% CHAPS at 62°C for 30 minutes each and two MAB washes (10 minutes each at room temperature). Animals were blocked in 1% blocking solution (Roche) in MAB at 4°C for two hours. DIG-AP-antibody (Roche) incubation followed for four hours at 4°C (1:2000 in blocking solution). Animals were washed in MAB at room temperature overnight and washed with MAB for 70 minutes changing the solution frequently. 2 × 5 minutes incubation steps followed in NTMT (0.1 M NaCl, 0.05 M MgCl2), 0.1 M Tris (pH 9.5), 0.1% Tween-20). Colour development was performed with a NBT/BCIP system (Roth) in the dark at 37°C for 55 minutes for *macpiwi*. For *macvasa *developing time was 10 minutes at 37°C and 8 minutes at room temperature. Frequent Ethanol washes were done followed by rehydration (3 × 5 minutes PBS). Animals were embedded in self-hardening Glycerin/Mowiol 4–88 solution. In situ hybridizations were analyzed and pictures taken using a Leica DM5000 microscope and a Pixera Penguin 600CL digital camera. Assembly and editing was performed with Adobe^® ^Photoshop^® ^7.0 Software. Template DNA for producing DIG-labeled riboprobe was produced by standard PCR techniques. For *macpiwi*, primer couples were TGCTCAAGCTGGTGTTGCTGGTC and GTCTTGTTGTTGTGCCGCGTGAG and for *macvasa*, primer couples were CCCGGTCGCTCTTATACTGACTCC and GGTTGGCACGGAACATCCTCTC. Probes were generated using a DIG RNA labeling KIT SP6/T7 (Roche) according to the manufacturer's protocol, revealing a *macpiwi *probe of 864 bp and a *macvasa *probe of 1067 bp. Partial sequences of *vasa*-(ANGU3256) and *piwi*-like (ANGU7606) genes were obtained from the *Macrostomum lignano *EST database [44]. Detailed information on both genes will be published separately.

### Hydroxyurea treatment and subtraction library

Test animals were kept under normal culture conditions [66] on glass petri dishes in a climate chamber on a diatom layer of *Nitzschia curvilineata *in Guillard's nutrient-enriched artificial seawater f/2 medium [67]. Antibiotics were added (Kanamycin 1:1000). Hydroxyurea (HU) concentration in the medium was 4.0 mM.

In order to identify differentially expressed genes specific for stem cells and germ line cells, a subtraction library was created by culturing two groups of animals on algae for 15 days. Group A consisted of HU-treated *M. lignano *(4 batches at 250 animals each) by soaking the animals in HU-solution. Group B were non-treated *M. lignano *(4 batches at 250 animals each). After 10 days of gradually increasing HU incubation (2.8 mM to 4.0 mM) control stainings with BrdU were performed and still showed 30–60 S-phase cells per animal in the gonads. The incubation time was continued until 15 days and total RNA was isolated. Total RNA of both groups of animals (HU-treated and non-treated) was checked on 1.2% Agarose Gel and sent to EVROGEN^® ^(Moscow, Russia). Evrogen made cDNA of both RNA samples, cloned 40 ng of purified cDNA into the pAL9 vector and used E. coli for transformation. Subtractive hybridization was performed using the SSH method (Suppression Subtractive Hybridization as described in [68,69]. The subtraction revealed 147 differentially expressed clones.

### BrdU/phos-H3 double labeling

Worms were soaked for 30 minutes in 5 mM BrdU (Bromodeoxyuridine, Sigma). After BrdU incorporation, animals were washed twice with f/2 and relaxed in 1:1 7.14% MgCl_2_:artificial sea water (ASW) for 5 minutes, followed by 15 minutes in pure 7.14% MgCl_2_. Animals are fixed for 1 hour at RT in 4% PFA in 0.1 M PBS, followed by multiple washing with PBS-T (0.1%). Protease treatment (0.22 μg/ml Protease XIV (30 min, 37°C) was followed by incubation for 10 minutes in 0.1 N HCl on ice and further denaturation in 2 N HCl (1 h, 37°C). A 3 × 5 minutes wash in PBS-T to remove acid completely was followed by 30 minutes blocking in BSA -T. Primary antibody (mouse-anti-BrdU, Roche), 1:600 in PBS-BSA-T was incubated at 4°C overnight. After washing with PBS-T (3 × 5 minutes), specimens were incubated with a secondary antibody (FITC-goat-anti-mouse (DakoCytomation), 1:150 at RT in darkness for 1 h and washed again with PBS-T for 3 × 5 minutes. Specimens were mounted in Vectashield (Vector Laboratories) and animals observed with a Leica DM5000 fluorescent microscope. For BrdU/phosH3 double labeling, the anti-phosphorylated H3 antibody (Upstate Biotechnology) was added to the primary antibody incubation in a concentration of 1:150 in PBS-BSA-T. For detection, a TRITC-swine-anti-rabbit antibody (DakoCytomation) was used at a final dilution of 1:150. The number of mitoses was counted using a Leica DM5000 fluorescent microscope and a mechanical tally counter. Because of the low number of mitotic cells, the use of a software-based quantification according to [70] was not necessary.

### Irradiation

Worms in f/2 culture medium of 5 mm depth were irradiated with 10, 20, 40 or 80 Gray (Gy) using gamma rays of an IBL-437C-instrument with a Cs-137 source (660 KeV; 700 cGy/min; 189 TBq nominal Activity). Animals were killed 3 hours, 24 hours, 7 days and 21 days after initial irradiation and examined for cell division (BrdU/H3-P) as well as *macpiwi *expression.

## Declaration of competing interests

The author(s) declare that they have no competing interests.

## Authors' contributions

DP carried out HU experiments, *macvasa *in situ hybridizations, participated in designing the study and drafted parts of the manuscript. KDM performed irradiation experiments and *macpiwi *in situ hybridizations. IP has run the large scale ISH pilot screen. GK participated in isolation of *macvasa*. MH has done HU experiments in relation with cell differentiation. PE was responsible for the technical aspect of irradiation. GB has contributed to irradiation, *macpiwi *results and manuscript drafting. VH was engaged in EST sequencing and preparation of the manuscript. PL has designed the study, performed irradiation experiments, interpreted results, and helped to draft the manuscript.

All authors read and approved the final manuscript.

## Supplementary Material

Additional File 1 Selected genes for pilot in situ screen. The table shows a list of genes that were selected for the pilot in situ screen including the clone numbers from the *M. lignano *EST project. Click here for file

Additional File 2 Orthology of *macpiwi*. Alignment of *macpiwi *with different *piwi *genes characterized in other flatworms. Click here for file

Additional File 3 Subtraction library clone list. Clone list from the subtraction library, containing clone name, length (bp), accession number, top ten BLAST homology, score, E value and the assigned category. Click here for file
