# Supplementary material for: The exceptional stem cell system of Macrostomum lignano: Screening for gene expression and studying cell proliferation by hydroxyurea treatment and irradiation
Source: Front Zool. 2007 Mar 9;4:9. doi: 10.1186/1742-9994-4-9 (PMC1828727; doi:10.1186/1742-9994-4-9)
Supplement: Additional File 1 — Selected genes for pilot in situ screen. The table shows a list of genes that were selected for the pilot in situ screen including the clone numbers from the M. lignano EST project. [file 1742-9994-4-9-S1.pdf]

Additional file 1:

Selected genes for pilot in situ screen. ANGU numbers according to EST-IDs from *M. lignano* EST project (http://macest.biology.ucla.edu/macest/)

1.	ANGU570	membrane-type frizzled related protein; Receptors
2.	ANGU781	chromosome/nuclear structure Histone H3, family 3a
3.	ANGU879	protein phosphatases Atg05580
4.	ANGU919	RNA binding ELAV-like 1
5.	ANGU1007	ribosomal components 40S ribosomal protein S3
6.	ANGU1040	translation elongation factor 1alpha
7.	ANGU1103	microtubules beta tubulin
8.	ANGU1138	Transcription factor Wolf-hirschhorn syndrome candidate 1
9.	ANGU1141	apoptosis TSARG2
10.	ANGU1217	bicaudal
11.	ANGU1293	HSP70
12.	ANGU1314	TOL-1/takeout
13.	ANGU1679	GABA
14.	ANGU1698	protein phosphatases Ser/Thr 2A
15.	ANGU1731	microtubules eml2 protein
16.	ANGU1740	chaperonin
17.	ANGU1810	microtubules tub-1
18.	ANGU1835	RNA binding Ribonuclear protein 97D
19.	ANGU1858	8-hydroxy-guanine glycosylase DNA repair
20.	ANGU1901	hormone/GF/temptin
21.	ANGU1907	Transcription factor zink protein 600
22.	ANGU1911	translational elongation factor 1alpha
23.	ANGU1931	post-translational modification
24.	ANGU1938	RNA processing similar to DEAD
25.	ANGU1972	endoctic receptor Endo180
26.	ANGU2050	SFRP-2
27.	ANGU2138	cyclophilin
28.	ANGU2156	ras-like
29.	ANGU2157	hormone/GF takeout
30.	ANGU2260	protein kinase B - raf
31.	ANGU2261	Transcription factor zink finger protein
32.	ANGU2295	STAT2
33.	ANGU2378	protein kinase adenylate kinase isoenzyme-2
34.	ANGU2434	Golgi unknown
35.	ANGU2700	Tetraspanin 66E; Adhesion
36.	ANGU2717	Transcription factor ETS DNA binding protein Yan
37.	ANGU2734	Transcription factor Fank-1
38.	ANGU2738	petidylprolyl isomerase; Post-Translational Modification
39.	ANGU2747	similar to armadillo-repeat containing…
40.	ANGU2750	Receptors CG2839
41.	ANGU2757	stress response: alcohol dehydrogenase class III
42.	ANGU2804	translation factor novel protein similar to…
43.	ANGU2833	Thick/thin filaments A60607 tropomyosin-fluke
44.	ANGU2876	cyclin B1
45.	ANGU2876	cyclin B1
46.	ANGU2887	translation elongation factor EF1gamma
47.	ANGU2903	microfilaments cofilin like protein
48.	ANGU2904	transcription factor similar to SOH1

49.	ANGU2936	membrane protein, induced TNF factor
50.	ANGU3044	xolloid
51.	ANGU3103	arginine kinase
52.	ANGU3143	transcription factor FLJ40321 protein
53.	ANGU3164	vertebrates RIKEN cDNA
54.	ANGU3215	degradation cathepsin L-like cystein proteinase A
55.	ANGU3256	vasa
56.	ANGU3332	protein kinase casein kinase 2 beta
57.	ANGU3379	transcription factor TAR
58.	ANGU3391	caspase-3
59.	ANGU3415	transcription factor KOW
60.	ANGU3466	membrane protein SCO-spondin
61.	ANGU3467	receptor growth hormone inducible transmembrane protein
62.	ANGU3482	cell cycle division nuclear distribution gene
63.	ANGU3546	translation factor elongation 4e
64.	ANGU3569	lamin
65.	ANGU3685	transducktion RACK G protein
66.	ANGU3769	transcription factor spacial delta
67.	ANGU3791	receptor GABA-BR1b
68.	ANGU3815	protein kinase CK2alpha SU
69.	ANGU3912	transcription factor FOXj3 protein
70.	ANGU3957	immunology preS1 binding protein
71.	ANGU4026	XWnt11
72.	ANGU4196	boule; RNA-binding protein
73.	ANGU4210	transcription factor Rec
74.	ANGU4277	DNA supercoiling-factor
75.	ANGU4381	ERK5
76.	ANGU4510	groucho
77.	ANGU4525	receptor TLR3
78.	ANGU4574	receptor nicotinic acetylcholine receptor
79.	ANGU4714	SIR2-like histone deacetylase
80.	ANGU5083	Xolloid
81.	ANGU5174	DNA-Topoisomerase
82.	ANGU5290	cell cycle division pescadilla homolog1
83.	ANGU5302	ribosomal components 60S
84.	ANGU5468	cell cycle division polo like kinase 4
85.	ANGU5731	stress response heat shock protein 60
86.	ANGU5871	insulin-like growth factor receptor
87.	ANGU5879	protein phosphatases Ser/Thr protein phosphatases
88.	ANGU5895	Smad4
89.	ANGU5903	transcription factor CCR4
90.	ANGU6039	transcrition factor zink finger protein 403
91.	ANGU6051	cdc2
92.	ANGU6098	transcription factor paired
93.	ANGU6130	Notch receptor protein
94.	ANGU6303	programmed cell death 6-interacting protein
95.	ANGU6519	transcription factor zinc finger PDZ5
96.	ANGU6547	transcription factor BTF3
97.	ANGU7183	tropomyosin 2
98.	ANGU7189	transcription factor EGF-response factor 2
99.	ANGU7606	piwi
100.	PCR	actin
101.	PCR	MCM2
