# Supplementary material for: The exceptional stem cell system of Macrostomum lignano: Screening for gene expression and studying cell proliferation by hydroxyurea treatment and irradiation
Source: Front Zool. 2007 Mar 9;4:9. doi: 10.1186/1742-9994-4-9 (PMC1828727; doi:10.1186/1742-9994-4-9)
Supplement: Additional File 2 — Orthology of macpiwi. Alignment of macpiwi with different piwi genes characterized in other flatworms. [file 1742-9994-4-9-S2.pdf]

Additional file 2:

Alignment of *macpiwi* to the different *piwi* genes characterized in the planarian flatworms *Schmidtea mediterranea* (Smedwi1 DQ186985, Smedwi2 DQ186986) and *Dugesia japonica* (DjPiwi1 AJ865376).

```
                  *        20         *        40         *        60         *        80         *       100
Macpiwi : MSQQPKGRGRARGRSGPRPEEVSSGASFAPAPAAPAPAPVAAARPAPPPGLSAQAGVAGPSGDQQNTGRGVGRAVERGGAAQQAGDPGQLSERMAGLRVG : 100
Smedwi1 : -------------------------------------------------------------------------------------------MDSTNVTTD :   9
Smedwi2 : ----------------------------------------------------------------------------------MEEIPVKVAKLEANGSET :  18
DjPiwi1 : -----------------------------------------------------------------------------------------MEIELIRKNLD :  11

                  *       120         *       140         *       160         *       180         *       200
Macpiwi : GEASGDRGGLQRKLLRFQLANVRPEGAAGKKGSSGAPWQVMTNFVRINTEDKWCAYQLAADFRPMLGAKPLREKLLN---DAQQVPKPFVYDGGCLYTKQ : 197
Smedwi1 : PNLRPRRGGLQRRFAIIEPA-LQPDTVTDKIGKDGRVWSLKSNYANFNIKVEGPFYMYDVVMENKSIRTKLELLLAYANSKNNGQP--FCFDGRRLFTTS : 106
Smedwi2 : RGKMGNRGGLRGTFRIVEPN-LQPADITEKTGNIGRTWKLQSNFTKFSVLRSEKFMMYDSVFSQVGLSPKVKLQFIVRVCQENKLPGAFCFDGRRLFTSE : 117
DjPiwi1 : GIRIERRGGLQKRFRIVEPD-LQPNETKDKIGNVGKVWRLQSNFTKFSVNKSEKYIMYDTTFSKMGLSPKLKLQILVRICKEYNLPGSFCYDGRRLFTSD : 110

                  *       220         *       240         *       260         *       280         *       300
Macpiwi : PLAPAEGVKFAASIKSWSGEVAIRQWGVIEKNTPQWLHLMNWVLGKTQSHLNMQMVGRNFYYPDQATKLPQHRMEVWPGFEMAIKPSDGPLMLHVDVSHK : 297
Smedwi1 : KWHSDEDTENLVIKD---VEISIRLLTTFLKNSEEYYQMINIVFNNIQIFMGQEKIGRQFFLGSNCESGGIRE--GPSFFKMDNFKILGGYSTTITRGTN : 201
Smedwi2 : KWHGESDIQEFSHDE---KKMTLRLVSTILPETEEYYQMINVLLNNLQVMLGQERIGKGYFLSPNITKDEVPRNSGPTFFETNSFKVLGGFGTTLQRGTK : 214
DjPiwi1 : NWTEGSDIKEFTIDE---KKVSLRLVSTFLSDTEEYYQMINVLLNNLQIIMGQEKIGKGYFLGSNSNKDNVVKNIGPTFFETNNFKVLGGFGTTLQRGTR : 207

                  *       320         *       340         *       360         *       380         *       400
Macpiwi : IIHMSTVLDVMYDMYNRNPGN-----FQAAATDALVGQIVLTRHNNKTYRIDAIEWDKRVTETFPRYNKSTRQSEEISLAQYFAQTYNVNLSDSEQPILM : 392
Smedwi1 : ENGTASTLLYLERINRVLNENNVVSVYRRDQIDQLIGRDIITKYNNKTYRISDIKEMD-------VNDEVQLGDKKISYLNYFKQRYNINLKNDKQPFVI : 294
Smedwi2 : PTGELTTLLYIERINRVLNDNSVMKAYNRRSIDQLIGRDIITKYNNKTYRISEIKEMN-------VDEKFEMGGRTLSYAEYFKERYNIRLTQGDQPFVL : 307
DjPiwi1 : PNGQLTTLLYIERINRVLNDNSVLVTYNRKNIDQLIGRDILTKYNNKTYRISEIKEMN-------INETFEMVGRTLSYAQYFKERYNINLTTEQQPFVL : 300

                  *       420         *       440         *       460         *       480         *       500
Macpiwi : SKPRKRDLPPTK-------------GGRREQAENVLLPPEVCFMTGLTDEMRADRNLMKDLATFTRIDPSTRCGKLVKFASTMAQNRECQRVLGEFGLAV : 479
Smedwi1 : SRVKRSMVRPKEK-------TENEPEGPTETDQSLSIPGELCYLCGFSDSERSNINLQKNLGTVLKREPRERLNDIRDFCKWTG-AGKSKDYMKSWGMCI : 386
Smedwi2 : TRVKKPMRRERKKKDEEGVEKEKEKEAPEEKDMTLNIPGELCFLCGFSDQEKSNMDLQKNLGCVLKREPRERLDDIPAYCNWIKNSDAATGMCNKWQLKI : 407
DjPiwi1 : TRVKKIARRDKRKE-------HNENEAPEDMDLTLNITGELCFLCGFSDQEKSNIDLQKNLGCVLKRGPRERLDDIPSFCDWIKTNNNSQGMSNKWGLKI : 393

                  *       520         *       540         *       560         *       580         *       600
Macpiwi : APNAVEATARQLAPETVQMTKPIRVDPERADWDNALKGNGMFQPVNCKNWIFVYSQRDEQAAADFCQKLSRASQSLGMSFGEPYWLAVAQDRDQLWVKTI : 579
Smedwi1 : EEQPITIRGRELAPVDIFSNEVKINTKIPDDWKFGEIKFDVPKGTAHRFGVLVVD-RNPSHFNNFIEDVKREIGRLRINYTMDSISSCRSNEVEDALTDF : 485
Smedwi2 : DNKPLEIEGRELPPCDVISGGSKINEKMGDDWKFGRVQFDIKRDRKHEIDVVIVD-RNDFQYKNFMNDVEQELRNMRIDARVGKVNTCGPNDVERCLNDA : 506
DjPiwi1 : DNTPLEIEGRELPHCDVISGGQRINEKIKDDWKFGKVQFNVQKGVKHEIDVVIVD-RNDSQYGNFLNDVEYEIKQLRFDAKIGRVLVCSANDVERSLNDV : 492

                  *       620         *       640         *       660         *       680         *       700
Macpiwi : EENIDQGLDLVFCLLPSNKKQRYDSIKRLCYVNKPVPSQCVLTK--TIRNPAKVMSVATKVALQISCKLGDVAMAVSIPMRRTMIVGMDTYHDKRQSVSV : 677
Smedwi1 : IR--VSKVHMALVFIPDD---KVYAKVKNFTMSTGLLTQCVTQRNGSNRDDRRRKTVADKSVMQMFSKLGYDPWGINLKMAPTMIVGLDTFHSKTGKRSV : 580
Smedwi2 : ARSGSGCAKMALVFVPDD---RVYAKVKSFTMSTGLLTQCVTTRNGTNRNDKRRKVVSSKTVMQIFAKFGYDPWTVEIKLRPTMIVGMDTYHNKSSKKSI : 603
DjPiwi1 : MRSGSGCAKIALVFVPDD---RVYAKVKSFTISTGLLTQCVTTRNGTNRNDKRRQVVSSKTVMQIFSKFGYDPWTIDIKMRPTMIVGMDTFHNKGSKKSI : 589

                  *       720         *       740         *       760         *       780         *       800
Macpiwi : QGIVFSLNETFTQYYSYSPIVKGGKAELHNRLEVGFNLALQKFREKNGDLPTRIILYRDGVGDSMLEEVKNSELLQLKQSLSKIYGERMSSLGFKAIVVK : 777
Smedwi1 : QASVFSISAKFSQYISFVN-SSKGKNEFHENLGKNFLTALTTFQNKFNTMPLRLIIYRDGVGDSQLAFTKKFETDAVMKMIEKIYEN--QTLPQIIYVVV : 677
Smedwi2 : QASVFSINSTFTQYMSFVVNSPKGRQEFHETLGKNFNLALEDFKKRYDILPQRILVFRDGVGDNQLQFTKNFEVDAMKPLIENIYKGNGFPVPQIIYVIV : 703
DjPiwi1 : HASVFSINSTFSQYMSFAN-SPKGKQEFHDTLSGNFKAALTEFKRIYKILPVRIMVYRDGVGDSQLQFTKQFEVDAMKPLVENIYKENGCQVPQIIYVIV : 688

                  *       820         *       840         *       860         *       880         *       900
Macpiwi : KLVSSRMFRKQGSQLRNPAPGTVLDDVITMPNFVDFFLVSQYVNQGTVTPTHYNMIEDVNDANIK--------PDQVQQLTYKLTHLYFNWPGTIRVPAP : 869
Smedwi1 : KKRISVKFFKDGA---NPNPGTVVDEKIVKPNFYEFYLVSQKTTKGTASPTNYNVLMDTKFTNKKTNEVSVMSPSVLQQITYSLTHLYFNWMGTIRVPVP : 774
Smedwi2 : KKRVGTKLFNRGN---NPNPGTVVDKEIVKPNFYEFFLVSQRTTKGTATPTNYNVLEDTRLLTKKG-TMDPMAPNELQKITYALTHLYFNWMGTIRVPVP : 799
DjPiwi1 : KKRIGTKLFNRGD---NANPGTIVDKEIVKPNFFEFFLVSQRVTRGTASPTNYNVLEDTRYQNKAG-IIEAITPNELQRITYTLTHLYFNWMGTIRVPVP : 784

                  *       920         *
Macpiwi : CHYAHRLAYLVGQNLMEEP----SPQICDRLFFL : 899
Smedwi1 : THYAHRLAELVGKIHRCVTPPAINDRIRERLFYL : 808
Smedwi2 : VHYAHRLAELVGKVHKCSNPVAINERIRNRLFYL : 833
DjPiwi1 : VHYAHKLAELIGKVHKCSTPALINERIRNRLFYL : 818
```
