# Supplementary material for: The exceptional stem cell system of Macrostomum lignano: Screening for gene expression and studying cell proliferation by hydroxyurea treatment and irradiation
Source: Front Zool. 2007 Mar 9;4:9. doi: 10.1186/1742-9994-4-9 (PMC1828727; doi:10.1186/1742-9994-4-9)
Supplement: Additional File 3 — Subtraction library clone list. Clone list from the subtraction library, containing clone name, length (bp), accession number, top ten BLAST homology, score, E value and the assigned category. [file 1742-9994-4-9-S3.pdf]

Additional file 3: Subtraction library BLAST results top 10. Engine: NCBI. Database: NR.

<table>
<tr><th>Clone</th><th>bp</th><th>ID</th><th>Homology</th><th>Score</th><th>E value</th><th>category</th></tr>
<tr><td></td><td></td><td></td><td></td><td></td><td></td><td></td></tr>
<tr><td>1_Zoo_I_D05_36_07</td><td>579</td><td>emb|CAE01426.2|</td><td>protein kinase A catalytic subunit 2 [Aspergillus fumigatus]</td><td>68</td><td>1,00E-10</td><td>RNA binding</td></tr>
<tr><td></td><td></td><td>gb|AAC67395.1|</td><td>mitogen- and stress-activated protein kinase-2 [Homo sapiens]</td><td>62</td><td>6,00E-09</td><td></td></tr>
<tr><td></td><td></td><td>ref|NP_003933.1|</td><td>ribosomal protein S6 kinase, 90kDa, polypeptide 4; ribosomal protein kinase B; mitogen- and stress-activated protein kinase 2; ribosomal protein S6 kinase, 90kD, polypeptide 4 [Homo sapiens] sp|O7567</td><td>62</td><td>6,00E-09</td><td></td></tr>
<tr><td></td><td></td><td>gb|AAH47896.1|</td><td>RPS6KA4 protein [Homo sapiens]</td><td>62</td><td>6,00E-09</td><td></td></tr>
<tr><td></td><td></td><td>ref|XP_408854.1|</td><td>hypothetical protein AN4717.2 [Aspergillus nidulans FGSC A4] gb|EAA60759.1| hypothetical protein AN4717.2 [Aspergillus nidulans FGSC A4]</td><td>62</td><td>7,00E-09</td><td></td></tr>
<tr><td></td><td></td><td>emb|CAG13167.1|</td><td>unnamed protein product [Tetraodon nigroviridis]</td><td>62</td><td>7,00E-09</td><td></td></tr>
<tr><td></td><td></td><td>gb|AAF15553.1|</td><td>Rsk-2 [Xenopus laevis]</td><td>61</td><td>1,00E-08</td><td></td></tr>
<tr><td></td><td></td><td>gb|AAH12964.1|</td><td>Ribosomal protein S6 kinase, polypeptide 4 [Mus musculus]</td><td>61</td><td>2,00E-08</td><td></td></tr>
<tr><td></td><td></td><td>gb|AAC69577.1|</td><td>ribosome S6 protein kinase [Homo sapiens]</td><td>61</td><td>2,00E-08</td><td></td></tr>
<tr><td></td><td></td><td>ref|NP_004746.2|</td><td>ribosomal protein S6 kinase, 90kDa, polypeptide 5 isoform a; mitogen- and stress-activated protein kinase 1; ribosomal protein S6 kinase, 90kD, polypeptide 5 [Homo sapiens] sp|O75582|K6A5_HUMAN F</td><td>61</td><td>2,00E-08</td><td></td></tr>
<tr><td>1_Zoo_I_F11_86_11</td><td>445</td><td>gb|AAC67395.1|</td><td>mitogen- and stress-activated protein kinase-2 [Homo sapiens]</td><td>46</td><td>2,00E-04</td><td>RNA binding</td></tr>
<tr><td></td><td></td><td>gb|AAH47896.1|</td><td>RPS6KA4 protein [Homo sapiens]</td><td>46</td><td>2,00E-04</td><td></td></tr>
<tr><td></td><td></td><td>ref|NP_003933.1|</td><td>ribosomal protein S6 kinase, 90kDa, polypeptide 4; ribosomal protein kinase B; mitogen- and stress-activated protein kinase 2; ribosomal protein S6 kinase, 90kD, polypeptide 4 [Homo sapiens] sp|O7567</td><td>46</td><td>2,00E-04</td><td></td></tr>
<tr><td></td><td></td><td>emb|CAA73554.1|</td><td>Serine/Threonine protein kinase [Sycon raphanus]</td><td>45</td><td>2,00E-04</td><td></td></tr>
<tr><td></td><td></td><td>emb|CAA66181.1|</td><td>G protein-coupled receptor kinase GRK4B [Rattus norvegicus]</td><td>45</td><td>3,00E-04</td><td></td></tr>
<tr><td></td><td></td><td>ref|NP_075217.1|</td><td>G protein-coupled receptor kinase 2, groucho gene related [Rattus norvegicus] sp|P70507|GRK4_RAT G protein-coupled receptor kinase 4 (G protein-coupled receptor kinase GRK4) emb|CAA66180.1| G</td><td>45</td><td>3,00E-04</td><td></td></tr>
<tr><td></td><td></td><td>gb|AAH12964.1|</td><td>Ribosomal protein S6 kinase, polypeptide 4 [Mus musculus]</td><td>45</td><td>4,00E-04</td><td></td></tr>
<tr><td></td><td></td><td>ref|NP_062370.1|</td><td>G protein-coupled receptor kinase 2, groucho gene related; G protein-coupled receptor kinase 4 [Mus musculus] sp|O70291|GRK4_MOUSE G protein-coupled receptor kinase 4 (G protein-coupled recept</td><td>44</td><td>5,00E-04</td><td></td></tr>
<tr><td></td><td></td><td>gb|AAA68709.2|</td><td>Protein kinase c protein 2, isoform c [Caenorhabditis elegans]</td><td>44</td><td>5,00E-04</td><td></td></tr>
<tr><td></td><td></td><td>pir||T15903</td><td>protein kinase C homolog - Caenorhabditis elegans</td><td>44</td><td>5,00E-04</td><td></td></tr>
<tr><td>1_Zoo_II_F02_110_12</td><td>326</td><td>emb|CAE66872.1|</td><td>Hypothetical protein CBG12250 [Caenorhabditis briggsae]</td><td>120</td><td>7,00E-27</td><td>RNA binding</td></tr>
<tr><td></td><td></td><td>ref|NP_194751.1|</td><td>small nuclear ribonucleoprotein F, putative / snRNP-F, putative / Sm protein F, putative [Arabidopsis thaliana] sp|Q9SUM2|RUXF_ARATH Probable small nuclear ribonucleoprotein F (snRNP-F) (Sm prote</td><td>119</td><td>1,00E-26</td><td></td></tr>
<tr><td></td><td></td><td>ref|XP_312266.1|</td><td>ENSANGP00000002801 [Anopheles gambiae] gb|EAA07677.2| ENSANGP00000002801 [Anopheles gambiae str. PEST]</td><td>118</td><td>3,00E-26</td><td></td></tr>
<tr><td></td><td></td><td>ref|XP_357414.1|</td><td>similar to SNRPF protein [Mus musculus]</td><td>117</td><td>4,00E-26</td><td></td></tr>
<tr><td></td><td></td><td>emb|CAG04169.1|</td><td>unnamed protein product [Tetraodon nigroviridis]</td><td>117</td><td>7,00E-26</td><td></td></tr>
<tr><td></td><td></td><td>dbj|BAD27986.1|</td><td>putative small nuclear ribonucleoprotein polypeptide F [Oryza sativa (japonica cultivar-group)]</td><td>117</td><td>7,00E-26</td><td></td></tr>
<tr><td></td><td></td><td>ref|XP_345815.1|</td><td>similar to Small nuclear ribonucleoprotein F (snRNP-F) (Sm protein F) (Sm-F) (SmF) [Rattus norvegicus]</td><td>116</td><td>1,00E-25</td><td></td></tr>
<tr><td></td><td></td><td>gb|AAH66015.1|</td><td>Unknown (protein for IMAGE:5715059) [Mus musculus]</td><td>116</td><td>1,00E-25</td><td></td></tr>
<tr><td></td><td></td><td>ref|NP_003086.1|</td><td>small nuclear ribonucleoprotein polypeptide F [Homo sapiens] sp|Q15356|RUXF_HUMAN Small nuclear ribonucleoprotein F (snRNP-F) (Sm protein F) (Sm-F) (SmF) pir||S55053 Sm protein F - human em</td><td>116</td><td>1,00E-25</td><td></td></tr>
<tr><td></td><td></td><td>emb|CAG33032.1|</td><td>SNRPF [Homo sapiens]</td><td>116</td><td>1,00E-25</td><td></td></tr>
<tr><td>1_Zoo_I_G01_7_13</td><td>557</td><td>ref|NP_172940.1|</td><td>expressed protein [Arabidopsis thaliana] gb|AAF79235.1| F10B6.27 [Arabidopsis thaliana] gb|AAL62389.1| unknown protein [Arabidopsis thaliana] gb|AAM48020.1| unknown protein [Arabidopsis thaliana]</td><td>64</td><td>1,00E-10</td><td>cell cycle/division</td></tr>
<tr><td></td><td></td><td>ref|NP_680337.2|</td><td>expressed protein [Arabidopsis thaliana]</td><td>62</td><td>8,00E-10</td><td></td></tr>
<tr><td></td><td></td><td>dbj|BAD29572.1|</td><td>putative ORFX [Oryza sativa (japonica cultivar-group)]</td><td>55</td><td>2,00E-09</td><td></td></tr>
<tr><td></td><td></td><td>gb|AAF74286.1|</td><td>ORFX [Lycopersicon esculentum] gb|AAO12185.1| fw2.2 [Lycopersicon esculentum] gb|AAO12186.1| fw2.2 [Lycopersicon esculentum] gb|AAO12187.1| fw2.2 [Lycopersicon esculentum] gb|AAO12188.1|</td><td>55</td><td>8,00E-09</td><td></td></tr>
<tr><td></td><td></td><td>gb|AAO12196.1|</td><td>fw2.2 [Lycopersicon pennellii]</td><td>55</td><td>8,00E-09</td><td></td></tr>
<tr><td></td><td></td><td>gb|AAO12190.1|</td><td>fw2.2 [Lycopersicon pimpinellifolium] gb|AAO12191.1| fw2.2 [Lycopersicon pimpinellifolium] gb|AAO12192.1| fw2.2 [Lycopersicon pimpinellifolium] gb|AAO12193.1| fw2.2 [Lycopersicon parviflorum] gb|AA</td><td>55</td><td>8,00E-09</td><td></td></tr>
<tr><td></td><td></td><td>gb|AAF74287.1|</td><td>ORFX [Lycopersicon pennellii]</td><td>55</td><td>8,00E-09</td><td></td></tr>
<tr><td></td><td></td><td>gb|AAO12189.1|</td><td>fw2.2 [Lycopersicon cheesmanii]</td><td>54</td><td>2,00E-08</td><td></td></tr>
<tr><td></td><td></td><td>ref|NP_172941.1|</td><td>expressed protein [Arabidopsis thaliana] gb|AAF79233.1| F10B6.29 [Arabidopsis thaliana] dbj|BAC43448.1| unknown protein [Arabidopsis thaliana] gb|AAO64083.1| unknown protein [Arabidopsis thaliana</td><td>57</td><td>2,00E-08</td><td></td></tr>
<tr><td></td><td></td><td>gb|AAT44867.1|</td><td>DUF614 protein [Branchiostoma belcheri tsingtaunese]</td><td>60</td><td>3,00E-08</td><td></td></tr>
<tr><td>1_Zoo_II_G02_111_14</td><td>442</td><td>emb|CAE75015.1|</td><td>Hypothetical protein CBG22919 [Caenorhabditis briggsae]</td><td>57</td><td>8,00E-08</td><td>cell cycle/division</td></tr>
<tr><td></td><td></td><td>ref|NP_501992.1|</td><td>protein phosphatase family member (4L516) [Caenorhabditis elegans] pir||T25259 phosphoprotein phosphatase (EC 3.1.3.16) T25B9.2 [similarity] - Caenorhabditis elegans emb|CAA94374.1| Hypothetica</td><td>57</td><td>1,00E-07</td><td></td></tr>
<tr><td></td><td></td><td>ref|NP_502041.1|</td><td>predicted CDS, protein phosphatase family member (4L719) [Caenorhabditis elegans] pir||T27314 phosphoprotein phosphatase (EC 3.1.3.16) Y69E1A.4 [similarity] - Caenorhabditis elegans emb|CAA222</td><td>57</td><td>1,00E-07</td><td></td></tr>
<tr><td></td><td></td><td>emb|CAE74022.1|</td><td>Hypothetical protein CBG21670 [Caenorhabditis briggsae]</td><td>56</td><td>2,00E-07</td><td></td></tr>
<tr><td></td><td></td><td>ref|NP_505470.1|</td><td>protein phosphatase family member (5K44) [Caenorhabditis elegans] pir||T21322 phosphoprotein phosphatase (EC 3.1.3.16) 1 F25B3.4 [similarity] - Caenorhabditis elegans emb|CAA94756.1| Hypothetica</td><td>55</td><td>2,00E-07</td><td></td></tr>
<tr><td></td><td></td><td>ref|NP_499229.1|</td><td>protein phosphatase family member (3L126) [Caenorhabditis elegans] pir||G88572 protein T16G12.7 [imported] - Caenorhabditis elegans emb|CAA83616.1| Hypothetical protein T16G12.7 [Caenorhabditis</td><td>55</td><td>3,00E-07</td><td></td></tr>
<tr><td></td><td></td><td>emb|CAE65057.1|</td><td>Hypothetical protein CBG09902 [Caenorhabditis briggsae]</td><td>55</td><td>3,00E-07</td><td></td></tr>
<tr><td></td><td></td><td>sp|P11612|PPY_DROME</td><td>Serine/threonine protein phosphatase PP-Y pir||PAFFY phosphoprotein phosphatase (EC 3.1.3.16) Y - fruit fly (Drosophila melanogaster) emb|CAA68808.1| unnamed protein product [Drosophila melanog</td><td>55</td><td>4,00E-07</td><td></td></tr>
<tr><td></td><td></td><td>ref|NP_476689.1|</td><td>CG10930-PA [Drosophila melanogaster] gb|AAF57771.1| CG10930-PA [Drosophila melanogaster] gb|AAL68035.1| AT05565p [Drosophila melanogaster]</td><td>55</td><td>4,00E-07</td><td></td></tr>
<tr><td></td><td></td><td>emb|CAE63788.1|</td><td>Hypothetical protein CBG08329 [Caenorhabditis briggsae]</td><td>54</td><td>6,00E-07</td><td></td></tr>
<tr><td>1_Zoo_I_A01_1_01</td><td>433</td><td>ref|NP_014536.1|</td><td>cell wall integrity and stress response component 3; Wsc3p [Saccharomyces cerevisiae] sp|Q12215|WSC3_YEAST Cell wall integrity and stress response component 3 precursor pir||S51892 probable me</td><td>41</td><td>0,004</td><td>stress response</td></tr>
<tr><td></td><td></td><td>gb|AAC02272.1|</td><td>mucin [Homo sapiens]</td><td>40</td><td>0,01</td><td></td></tr>
<tr><td></td><td></td><td>ref|NP_997126.2|</td><td>mucin 19; sublingual apomucin [Mus musculus] gb|AAS77382.2| Muc19 precursor [Mus musculus]</td><td>40</td><td>0,01</td><td></td></tr>
<tr><td></td><td></td><td>ref|XP_354871.1|</td><td>similar to sublingual apomucin [Mus musculus]</td><td>40</td><td>0,01</td><td></td></tr>
<tr><td></td><td></td><td>gb|AAF13032.1|</td><td>intestinal mucin 3 [Homo sapiens]</td><td>40</td><td>0,01</td><td></td></tr>
<tr><td></td><td></td><td>ref|XP_390611.1|</td><td>hypothetical protein FG10435.1 [Gibberella zeae PH-1] gb|EAA68209.1| hypothetical protein FG10435.1 [Gibberella zeae PH-1]</td><td>40</td><td>0,01</td><td></td></tr>
<tr><td></td><td></td><td>gb|AAO49800.1|</td><td>MUC19 [Mus musculus]</td><td>40</td><td>0,01</td><td></td></tr>
<tr><td></td><td></td><td>gb|AAO38851.1|</td><td>sublingual apomucin [Mus musculus]</td><td>40</td><td>0,01</td><td></td></tr>
<tr><td></td><td></td><td>ref|XP_168578.4|</td><td>mucin 3B [Homo sapiens]</td><td>39</td><td>0,022</td><td></td></tr>
<tr><td></td><td></td><td>ref|XP_374501.1|</td><td>similar to intestinal mucin 3 [Homo sapiens]</td><td>39</td><td>0,022</td><td></td></tr>
</table>

Additional file 3: Subtraction library BLAST results top 10. Engine: NCBI. Database: NR.

| Clone | bp | ID | Homology | Score | E value | category |
|---|---|---|---|---|---|---|
| 1 Zoo I F03 22 11 | 434 | ref\|XP 354871.1\| | similar to sublingual apomucin [Mus musculus] | 42 | 0,003 | stress response |
| | | ref\|NP 997126.2\| | mucin 19; sublingual apomucin [Mus musculus] gb\|AAS77382.2\| Muc19 precursor [Mus musculus] | 42 | 0,003 | |
| | | gb\|AAO38851.1\| | sublingual apomucin [Mus musculus] | 42 | 0,003 | |
| | | gb\|AAO49800.1\| | MUC19 [Mus musculus] | 42 | 0,003 | |
| | | emb\|CAC83675.1\| | mucin 5 [Homo sapiens] | 41 | 0,006 | |
| | | ref\|NP 014536.1\| | cell wall integrity and stress response component 3; Wsc3p [Saccharomyces cerevisiae] sp\|Q12215\|WSC3_YEAST Cell wall integrity and stress response component 3 precursor pir\|\|S51892 probable me | 40 | 0,007 | |
| | | ref\|XP 390611.1\| | hypothetical protein FG10435.1 [Gibberella zeae PH-1] gb\|EAA68209.1\| hypothetical protein FG10435.1 [Gibberella zeae PH-1] | 40 | 0,013 | |
| | | gb\|AAF13032.1\| | intestinal mucin 3 [Homo sapiens] | 39 | 0,028 | |
| | | pir\|\|S53363 | mucin 5AC (clone JER58) - human (fragment) emb\|CAA84032.1\| mucin [Homo sapiens] | 39 | 0,028 | |
| | | gb\|AAC02272.1\| | mucin [Homo sapiens] | 39 | 0,028 | |
| 1 Zoo I F05 38 11 | 581 | gb\|AAF80601.1\| | FMRFamide-gated Na+ channel [Helisoma trivolvis] | 48 | 1,00E-04 | transduction |
| | | ref\|NP 491214.1\| | degenerin mec-4 family member (1E341) [Caenorhabditis elegans] sp\|O01635\|DEGY_CAEEL Degenerin-like protein ZK770.1 in chromosome I pir\|\|T34468 hypothetical protein ZK770.1 - Caenorhabditis e | 39 | 0,004 | |
| | | sp\|Q25011\|FANA HELA | FMRFamide-activated amiloride-sensitive sodium channel (FANACH) pir\|\|S68434 FMRFamide-activated sodium channel protein, amiloride-sensitive - brown garden snail emb\|CAA63084.1\| FMRFamide-a | 43 | 0,005 | |
| | | emb\|CAE66660.1\| | Hypothetical protein CBG11997 [Caenorhabditis briggsae] | 39 | 0,006 | |
| | | gb\|AAK20896.1\| | FMRFamide-gated and pH-modulated sodium channel [Lymnaea stagnalis] | 40 | 0,03 | |
| | | ref\|XP 321465.1\| | ENSANGP00000008568 [Anopheles gambiae] gb\|EAA00980.1\| ENSANGP00000008568 [Anopheles gambiae str. PEST] | 39 | 0,067 | |
| | | ref\|XP 309730.1\| | ENSANGP00000012636 [Anopheles gambiae] gb\|EAA05433.1\| ENSANGP00000012636 [Anopheles gambiae str. PEST] | 38 | 0,11 | |
| | | ref\|XP 309733.1\| | ENSANGP00000012506 [Anopheles gambiae] gb\|EAA05551.1\| ENSANGP00000012506 [Anopheles gambiae str. PEST] | 37 | 0,2 | |
| | | sp\|O13263\|SCG2 XENL | Amiloride-sensitive sodium channel gamma-2-subunit (Epithelial Na(+) channel gamma-2 subunit) (Gamma-2 ENAC) (Nonvoltage-gated sodium channel 1 gamma-2 subunit) (SCNEG2) (Gamma-2 NACH | 37 | 0,25 | |
| | | ref\|XP 309731.1\| | ENSANGP00000012533 [Anopheles gambiae] gb\|EAA05580.1\| ENSANGP00000012533 [Anopheles gambiae str. PEST] | 37 | 0,33 | |
| 1 Zoo I H02 16 16 | 523 | ref\|NP 989985.1\| | CocoaCrisp [Gallus gallus] gb\|AAK16497.1\| CocoaCrisp [Gallus gallus] | 50 | 3,00E-05 | transduction |
| | | emb\|CAF98015.1\| | unnamed protein product [Tetraodon nigroviridis] | 49 | 5,00E-05 | |
| | | ref\|NP 956764.1\| | hypothetical protein MGC63636 [Danio rerio] gb\|AAH55176.1\| Hypothetical protein MGC63636 [Danio rerio] | 49 | 6,00E-05 | |
| | | dbj\|BAB55081.1\| | unnamed protein product [Homo sapiens] | 47 | 2,00E-04 | |
| | | gb\|AAQ89150.1\| | trypsin inhibitor [Homo sapiens] | 47 | 2,00E-04 | |
| | | ref\|NP 113664.1\| | hypothetical protein DKFZp434B044 [Homo sapiens] emb\|CAB66795.1\| hypothetical protein [Homo sapiens] | 47 | 2,00E-04 | |
| | | gb\|AAH63012.1\| | Unknown (protein for MGC:74865) [Homo sapiens] | 45 | 5,00E-04 | |
| | | ref\|XP 237258.2\| | similar to CocoaCrisp [Rattus norvegicus] | 45 | 5,00E-04 | |
| | | sp\|P17326\|AT1A ARTS | Sodium/potassium-transporting ATPase alpha chain (Sodium pump) (Na+/K+ ATPase) pir\|\|S06635 Na+/K+-exchanging ATPase (EC 3.6.3.9) alpha chain (clone alpha-2850) - brine shrimp emb\|CAA6881 | 45 | 0,001 | |
| | | ref\|NP 113649.1\| | CocoaCrisp [Homo sapiens] gb\|AAG43287.1\| putative secretory protein precursor [Homo sapiens] gb\|AAK16495.1\| CocoaCrisp [Homo sapiens] gb\|AAH20514.1\| CocoaCrisp [Homo sapiens] gb\|AAQ8892 | 45 | 0,001 | |
| 1 Zoo I C06 43 06 | 473 | ref\|XP 390611.1\| | hypothetical protein FG10435.1 [Gibberella zeae PH-1] gb\|EAA68209.1\| hypothetical protein FG10435.1 [Gibberella zeae PH-1] | 47 | 1,00E-04 | receptors |
| | | ref\|XP 331659.1\| | hypothetical protein [Neurospora crassa] gb\|EAA35466.1\| hypothetical protein [Neurospora crassa] emb\|CAE76318.1\| related to glyoxal oxidase precursor [Neurospora crassa] | 47 | 2,00E-04 | |
| | | ref\|XP 391273.1\| | hypothetical protein FG11097.1 [Gibberella zeae PH-1] gb\|EAA74748.1\| hypothetical protein FG11097.1 [Gibberella zeae PH-1] | 46 | 3,00E-04 | |
| | | ref\|XP 385939.1\| | hypothetical protein FG05763.1 [Gibberella zeae PH-1] gb\|EAA75838.1\| hypothetical protein FG05763.1 [Gibberella zeae PH-1] | 45 | 4,00E-04 | |
| | | ref\|XP 323510.1\| | hypothetical protein [Neurospora crassa] gb\|EAA31492.1\| hypothetical protein [Neurospora crassa] | 45 | 5,00E-04 | |
| | | emb\|CAD11404.1\| | hypothetical protein [Neurospora crassa] | 45 | 5,00E-04 | |
| | | gb\|EAA56004.1\| | hypothetical protein MG01655.4 [Magnaporthe grisea 70-15] | 44 | 0,001 | |
| | | sp\|Q90Y90\|KRM1 XENL | Kremen protein 1 precursor (Kringle-containing protein marking the eye and the nose) (Dickkopf receptor) dbj\|BAB64294.1\| KREMEN [Xenopus laevis] | 44 | 0,001 | |
| | | ref\|XP 409797.1\| | hypothetical protein AN5660.2 [Aspergillus nidulans FGSC A4] gb\|EAA62753.1\| hypothetical protein AN5660.2 [Aspergillus nidulans FGSC A4] | 44 | 0,001 | |
| | | emb\|CAC18170.2\| | related to beta-1, 3 exoglucanase precursor [Neurospora crassa] | 44 | 0,001 | |
| 1 Zoo I E01 5 09 | 563 | gb\|AAN64661.1\| | kremen2 [Xenopus laevis] | 47 | 2,00E-04 | receptors |
| | | ref\|XP 385939.1\| | hypothetical protein FG05763.1 [Gibberella zeae PH-1] gb\|EAA75838.1\| hypothetical protein FG05763.1 [Gibberella zeae PH-1] | 44 | 0,001 | |
| | | emb\|CAG08177.1\| | unnamed protein product [Tetraodon nigroviridis] | 43 | 0,004 | |
| | | ref\|NP 446101.1\| | kringle-containing transmembrane protein 1 [Rattus norvegicus] sp\|Q924S4\|KRM1_RAT Kremen protein 1 precursor (Kringle-containing protein marking the eye and the nose) (Dickkopf receptor) dbj\|BAB | 43 | 0,004 | |
| | | sp\|Q96MU8\|KRM1 HUM | Kremen protein 1 precursor (Kringle-containing protein marking the eye and the nose) (Dickkopf receptor) | 42 | 0,006 | |
| | | ref\|NP 700358.1\| | kringle-containing transmembrane protein 1 isoform 1 precursor; kringle-coding gene marking the eye and the nose; kringle containing transmembrane protein [Homo sapiens] dbj\|BAB40969.1\| kringle-cor | 42 | 0,006 | |
| | | ref\|NP 115772.1\| | kringle-containing transmembrane protein 1; kringle-coding gene marking the eye and the nose [Mus musculus] sp\|Q99N43\|KRM1_MOUSE Kremen protein 1 precursor (Kringle-containing protein marking | 42 | 0,006 | |
| | | ref\|NP 114434.3\| | kringle-containing transmembrane protein 1 isoform 2 precursor; kringle-coding gene marking the eye and the nose; kringle containing transmembrane protein [Homo sapiens] | 42 | 0,006 | |
| | | dbj\|BAB71180.1\| | unnamed protein product [Homo sapiens] | 42 | 0,006 | |
| | | dbj\|BAC38952.1\| | unnamed protein product [Mus musculus] | 42 | 0,006 | |
| 1 Zoo I E09 69 09 | 471 | ref\|XP 331659.1\| | hypothetical protein [Neurospora crassa] gb\|EAA35466.1\| hypothetical protein [Neurospora crassa] emb\|CAE76318.1\| related to glyoxal oxidase precursor [Neurospora crassa] | 47 | 2,00E-04 | receptors |
| | | ref\|XP 385939.1\| | hypothetical protein FG05763.1 [Gibberella zeae PH-1] gb\|EAA75838.1\| hypothetical protein FG05763.1 [Gibberella zeae PH-1] | 46 | 2,00E-04 | |
| | | ref\|XP 390611.1\| | hypothetical protein FG10435.1 [Gibberella zeae PH-1] gb\|EAA68209.1\| hypothetical protein FG10435.1 [Gibberella zeae PH-1] | 46 | 3,00E-04 | |
| | | ref\|XP 391273.1\| | hypothetical protein FG11097.1 [Gibberella zeae PH-1] gb\|EAA74748.1\| hypothetical protein FG11097.1 [Gibberella zeae PH-1] | 45 | 4,00E-04 | |
| | | gb\|EAA56004.1\| | hypothetical protein MG01655.4 [Magnaporthe grisea 70-15] | 45 | 5,00E-04 | |
| | | sp\|Q90Y90\|KRM1 XENL | Kremen protein 1 precursor (Kringle-containing protein marking the eye and the nose) (Dickkopf receptor) dbj\|BAB64294.1\| KREMEN [Xenopus laevis] | 45 | 5,00E-04 | |
| | | ref\|XP 409797.1\| | hypothetical protein AN5660.2 [Aspergillus nidulans FGSC A4] gb\|EAA62753.1\| hypothetical protein AN5660.2 [Aspergillus nidulans FGSC A4] | 45 | 7,00E-04 | |
| | | ref\|XP 323510.1\| | hypothetical protein [Neurospora crassa] gb\|EAA31492.1\| hypothetical protein [Neurospora crassa] | 44 | 0,001 | |
| | | emb\|CAD11404.1\| | hypothetical protein [Neurospora crassa] | 44 | 0,001 | |
| | | emb\|CAC18170.2\| | related to beta-1, 3 exoglucanase precursor [Neurospora crassa] | 44 | 0,001 | |

Additional file 3: Subtraction library BLAST results top 10. Engine: NCBI. Database: NR.

| Clone | bp | ID | Homology | Score | E value | category |
|---|---|---|---|---|---|---|
| 1_Zoo_II_A02_105_02 | 440 | ref\|XP_390611.1\| | hypothetical protein FG10435.1 [Gibberella zeae PH-1] gb\|EAA68209.1\| hypothetical protein FG10435.1 [Gibberella zeae PH-1] | 47 | 8,00E-05 | receptors |
| | | ref\|XP_331659.1\| | hypothetical protein [Neurospora crassa] gb\|EAA35466.1\| hypothetical protein [Neurospora crassa] emb\|CAE76318.1\| related to glyoxal oxidase precursor [Neurospora crassa] | 47 | 1,00E-04 | |
| | | ref\|XP_391273.1\| | hypothetical protein FG11097.1 [Gibberella zeae PH-1] gb\|EAA74748.1\| hypothetical protein FG11097.1 [Gibberella zeae PH-1] | 46 | 2,00E-04 | |
| | | ref\|XP_385939.1\| | hypothetical protein FG05763.1 [Gibberella zeae PH-1] gb\|EAA75838.1\| hypothetical protein FG05763.1 [Gibberella zeae PH-1] | 45 | 2,00E-04 | |
| | | emb\|CAD11404.1\| | hypothetical protein [Neurospora crassa] | 45 | 3,00E-04 | |
| | | ref\|XP_323510.1\| | hypothetical protein [Neurospora crassa] gb\|EAA31492.1\| hypothetical protein [Neurospora crassa] | 45 | 3,00E-04 | |
| | | sp\|Q90Y90\|KRM1_XENL | Kremen protein 1 precursor (Kringle-containing protein marking the eye and the nose) (Dickkopf receptor) dbj\|BAB64294.1\| KREMEN [Xenopus laevis] | 44 | 5,00E-04 | |
| | | gb\|EAA56004.1\| | hypothetical protein MG01655.4 [Magnaporthe grisea 70-15] | 44 | 5,00E-04 | |
| | | emb\|CAC18170.2\| | related to beta-1, 3 exoglucanase precursor [Neurospora crassa] | 44 | 7,00E-04 | |
| | | ref\|XP_409797.1\| | hypothetical protein AN5660.2 [Aspergillus nidulans FGSC A4] gb\|EAA62753.1\| hypothetical protein AN5660.2 [Aspergillus nidulans FGSC A4] | 44 | 7,00E-04 | |
| 1_Zoo_I_C05_35_05 | 447 | ref\|XP_393020.1\| | similar to CG6120-PA [Apis mellifera] | 41 | 0,006 | cell-cell communication |
| | | pir\|\|T22544 | hypothetical protein F53B6.1 - Caenorhabditis elegans | 40 | 0,01 | |
| | | ref\|NP_492404.1\| | tetraspanin family member (tsp-15) [Caenorhabditis elegans] emb\|CAB03120.2\| C. elegans TSP-15 protein (corresponding sequence F53B6.1) [Caenorhabditis elegans] | 40 | 0,01 | |
| | | emb\|CAE66995.1\| | Hypothetical protein CBG12393 [Caenorhabditis briggsae] | 40 | 0,01 | |
| | | gb\|AAP36787.1\| | Homo sapiens CD63 antigen (melanoma 1 antigen) [synthetic construct] | 37 | 0,062 | |
| | | gb\|AAH67604.1\| | Unknown (protein for IMAGE:6959852) [Danio rerio] | 37 | 0,062 | |
| | | gb\|AAH76407.1\| | Unknown (protein for MGC:100919) [Danio rerio] | 37 | 0,062 | |
| | | ref\|NP_001771.1\| | CD63 antigen; melanoma 1 antigen; ocular melanoma-associated antigen; melanoma-associated antigen ME491; lysosome-associated membrane glycoprotein 3; granulophysin; melanoma-associated an | 37 | 0,062 | |
| | | gb\|AAM34259.1\| | lysosome-associated membrane protein-3 variant [Homo sapiens] | 37 | 0,062 | |
| | | gb\|AAH74552.1\| | Unknown (protein for MGC:69509) [Xenopus tropicalis] | 36 | 0,18 | |
| 1_Zoo_I_D03_20_07 | 381 | gb\|EAL22113.1\| | hypothetical protein CNBC2510 [Cryptococcus neoformans var. neoformans B-3501A] | 42 | 0,002 | cell-cell communication |
| | | ref\|XP_331659.1\| | hypothetical protein [Neurospora crassa] gb\|EAA35466.1\| hypothetical protein [Neurospora crassa] emb\|CAE76318.1\| related to glyoxal oxidase precursor [Neurospora crassa] | 40 | 0,011 | |
| | | sp\|P83097\|WSCK_DRON | Putative tyrosine-protein kinase Wsck precursor | 39 | 0,018 | |
| | | ref\|NP_733018.1\| | CG31127-PA [Drosophila melanogaster] gb\|AAN14006.1\| CG31127-PA [Drosophila melanogaster] gb\|AAN71639.1\| SD05152p [Drosophila melanogaster] | 39 | 0,018 | |
| | | ref\|XP_387414.1\| | hypothetical protein FG07238.1 [Gibberella zeae PH-1] gb\|EAA77836.1\| hypothetical protein FG07238.1 [Gibberella zeae PH-1] | 38 | 0,04 | |
| | | ref\|XP_383926.1\| | hypothetical protein FG03750.1 [Gibberella zeae PH-1] gb\|EAA71442.1\| hypothetical protein FG03750.1 [Gibberella zeae PH-1] | 38 | 0,052 | |
| | | gb\|EAL19732.1\| | hypothetical protein CNBG3600 [Cryptococcus neoformans var. neoformans B-3501A] | 37 | 0,089 | |
| | | ref\|XP_220206.2\| | similar to Kremen2 protein [Rattus norvegicus] | 36 | 0,15 | |
| | | ref\|NP_082692.1\| | kringle-containing transmembrane protein 2 [Mus musculus] sp\|Q8K1S7\|KRM2_MOUSE Kremen protein 2 precursor (Kringle-containing protein marking the eye and the nose) (Dickkopf receptor 2) emb\|C | 36 | 0,15 | |
| | | ref\|XP_139908.1\| | similar to hypothetical protein MGC10791 [Mus musculus] | 36 | 0,15 | |
| 1_Zoo_I_B09_66_03 | 593 | emb\|CAE65829.1\| | Hypothetical protein CBG10951 [Caenorhabditis briggsae] | 41 | 0,019 | cell-cell communication |
| | | sp\|O35566\|C151_MOUS | Platelet-endothelial tetraspan antigen 3 (PETA-3) (GP27) (Membrane glycoprotein SFA-1) (CD151 antigen) gb\|AAC25976.1\| PETA-3 [Mus musculus] gb\|AAC25952.1\| platelet endothelial tetraspan antiger | 39 | 0,054 | |
| | | ref\|NP_033972.1\| | CD151 antigen [Mus musculus] dbj\|BAA22447.1\| SFA-1 [Mus musculus] | 39 | 0,054 | |
| | | ref\|NP_071968.1\| | CD151 antigen; platelet endothelial tetraspan antigen-3 [Rattus norvegicus] sp\|Q9QZA6\|C151_RAT Platelet endothelial tetraspan antigen 3 (CD151 antigen) gb\|AAF05763.2\| platelet endothelial tetraspan | 39 | 0,071 | |
| | | gb\|AAH12236.1\| | Cd151 protein [Mus musculus] | 37 | 0,27 | |
| | | ref\|XP_372453.1\| | similar to RIKEN cDNA 1110014F12 [Homo sapiens] | 35 | 0,78 | |
| | | ref\|NP_666285.1\| | hypothetical protein MGC30714 [Mus musculus] gb\|AAH24685.1\| Hypothetical protein MGC30714 [Mus musculus] | 35 | 1 | |
| | | ref\|NP_477766.1\| | wsv244 [shrimp white spot syndrome virus] gb\|AAL33248.1\| wsv244 [shrimp white spot syndrome virus] | 34 | 1,7 | |
| | | gb\|AAK77790.1\| | ORF121 [shrimp white spot syndrome virus] gb\|AAL89167.1\| WSSV299 [shrimp white spot syndrome virus] | 34 | 1,7 | |
| | | ref\|XP_394297.1\| | similar to ENSANGP00000017817 [Apis mellifera] | 34 | 2,3 | |
| 1_Zoo_I_E05_37_09 | 596 | emb\|CAE65829.1\| | Hypothetical protein CBG10951 [Caenorhabditis briggsae] | 41 | 0,019 | cell-cell communication |
| | | ref\|NP_071968.1\| | CD151 antigen; platelet endothelial tetraspan antigen-3 [Rattus norvegicus] sp\|Q9QZA6\|C151_RAT Platelet endothelial tetraspan antigen 3 (CD151 antigen) gb\|AAF05763.2\| platelet endothelial tetraspan | 40 | 0,025 | |
| | | sp\|O35566\|C151_MOUS | Platelet-endothelial tetraspan antigen 3 (PETA-3) (GP27) (Membrane glycoprotein SFA-1) (CD151 antigen) gb\|AAC25976.1\| PETA-3 [Mus musculus] gb\|AAC25952.1\| platelet endothelial tetraspan antiger | 40 | 0,025 | |
| | | ref\|NP_033972.1\| | CD151 antigen [Mus musculus] dbj\|BAA22447.1\| SFA-1 [Mus musculus] | 40 | 0,025 | |
| | | gb\|AAH12236.1\| | Cd151 protein [Mus musculus] | 38 | 0,12 | |
| | | dbj\|BAD04070.1\| | CD63 antigen [Felis catus] | 38 | 0,16 | |
| | | sp\|P48509\|C151_HUMA | Platelet-endothelial tetraspan antigen 3 (PETA-3) (GP27) (Membrane glycoprotein SFA-1) (CD151 antigen) dbj\|BAA06229.1\| CD151 [Homo sapiens] gb\|AAK14179.1\| hemidesmosomal tetraspanin CD151 | 37 | 0,21 | |
| | | ref\|NP_999939.1\| | zgc:63823 [Danio rerio] gb\|AAH55255.1\| Zgc:63823 [Danio rerio] | 37 | 0,27 | |
| | | gb\|AAP36787.1\| | Homo sapiens CD63 antigen (melanoma 1 antigen) [synthetic construct] | 36 | 0,61 | |
| | | ref\|NP_001771.1\| | CD63 antigen; melanoma 1 antigen; ocular melanoma-associated antigen; melanoma-associated antigen ME491; lysosome-associated membrane glycoprotein 3; granulophysin; melanoma-associated an | 36 | 0,61 | |
| 1_Zoo_I_F02_14_12 | 495 | gb\|AAM73990.1\| | alpha-tubulin 3 [Strongylocentrotus droebechiensis] | 267 | 8,00E-71 | cytoscelet |
| | | sp\|P18258\|TBA1_PARLI | TUBULIN ALPHA-1 CHAIN pir\|\|S11207 tubulin alpha chain - sea urchin (Paracentrotus lividus) emb\|CAA37680.1\| unnamed protein product [Paracentrotus lividus] | 266 | 1,00E-70 | |
| | | gb\|AAP80598.1\| | putative alpha-tubulin [Oikopleura dioica] | 266 | 1,00E-70 | |
| | | pir\|\|A60671 | tubulin alpha chain - sea urchin (Paracentrotus lividus) | 266 | 2,00E-70 | |
| | | gb\|AAP80595.1\| | putative alpha-tubulin [Oikopleura dioica] | 266 | 2,00E-70 | |
| | | gb\|AAB07727.1\| | alpha-1 tubulin [Hirudo medicinalis] gb\|AAB07890.1\| alpha-1 tubulin [Hirudo medicinalis] | 266 | 2,00E-70 | |
| | | sp\|P41383\|TBA2_PATV | TUBULIN ALPHA-2/ALPHA-4 CHAIN pir\|\|S42033 tubulin alpha chain - common limpet emb\|CAA54712.1\| alpha tubulin [Patella vulgata] emb\|CAA55978.1\| alpha tubulin 2 [Patella vulgata] | 265 | 2,00E-70 | |
| | | gb\|AAP49551.1\| | alpha-tubulin [Aurelia aurita] | 265 | 2,00E-70 | |
| | | gb\|AAC97929.1\| | alpha tubulin [Notothenia coriiceps] gb\|AAG15324.1\| alpha tubulin [Notothenia coriiceps] | 265 | 2,00E-70 | |
| | | ref\|XP_147357.1\| | tubulin alpha 6 [Mus musculus] | 265 | 3,00E-70 | |

Additional file 3: Subtraction library BLAST results top 10. Engine: NCBI. Database: NR.

| Clone | bp | ID | Homology | Score | E value | category |
|---|---|---|---|---|---|---|
| 1 Zoo I G04 31 14 | 448 | ref\|NP 729025.1\| | CG32238-PA [Drosophila melanogaster] gb\|AAF47928.1\| CG32238-PA [Drosophila melanogaster] | 45 | 2,00E-04 | cytoscelet |
| | | ref\|NP 573197.2\| | CG8918-PA [Drosophila melanogaster] gb\|AAF48704.1\| CG8918-PA [Drosophila melanogaster] | 45 | 2,00E-04 | |
| | | gb\|AAK92989.1\| | GH21352p [Drosophila melanogaster] | 45 | 2,00E-04 | |
| | | sp\|Q80UG8\|TTL4 MOUS | Tubulin tyrosine ligase-like protein 4 dbj\|BAC97885.1\| mKIAA0173 protein [Mus musculus] | 44 | 5,00E-04 | |
| | | ref\|XP 237292.2\| | similar to Hypothetical protein KIAA0173 [Rattus norvegicus] | 44 | 5,00E-04 | |
| | | ref\|XP 129771.3\| | expressed sequence AI451681 [Mus musculus] | 44 | 5,00E-04 | |
| | | sp\|Q14679\|TTL4 HUMA | Tubulin tyrosine ligase-like protein 4 | 44 | 5,00E-04 | |
| | | ref\|NP 055455.2\| | tubulin tyrosine ligase-like family, member 4 [Homo sapiens] gb\|AAH21707.1\| Tubulin tyrosine ligase-like family, member 4 [Homo sapiens] | 44 | 5,00E-04 | |
| | | dbj\|BAA11490.2\| | KIAA0173 [Homo sapiens] | 44 | 5,00E-04 | |
| | | ref\|XP 092778.3\| | similar to RIKEN cDNA 4930509O20 [Homo sapiens] | 44 | 7,00E-04 | |
| 1 Zoo I G05 39 13 | 545 | emb\|CAF96334.1\| | unnamed protein product [Tetraodon nigroviridis] | 44 | 0,002 | cytoscelet |
| | | ref\|XP 195428.2\| | similar to dJ366N23.3 (NYD-TSPG protein) [Mus musculus] | 43 | 0,004 | |
| | | ref\|XP 092778.3\| | similar to RIKEN cDNA 4930509O20 [Homo sapiens] | 42 | 0,005 | |
| | | sp\|P38160\|TTL PIG | Tubulin--tyrosine ligase (TTL) pir\|\|A45443 tubulin-tyrosine ligase (EC 6.3.2.25) [validated] - pig emb\|CAA48494.1\| tubulin-tyrosine ligase [Sus scrofa] | 42 | 0,005 | |
| | | emb\|CAD98091.1\| | hypothetical protein [Homo sapiens] | 41 | 0,012 | |
| | | ref\|NP 714923.1\| | tubulin tyrosine ligase; tubulin-tyrosine ligase; 2410003M22Rik [Homo sapiens] sp\|Q8NG68\|TTL_HUMAN Tubulin--tyrosine ligase (TTL) gb\|AAH36819.1\| TTL protein [Homo sapiens] dbj\|BAC06832.2\| tubu | 41 | 0,012 | |
| | | ref\|XP 217869.2\| | similar to dJ366N23.3 (NYD-TSPG protein) [Rattus norvegicus] | 41 | 0,015 | |
| | | ref\|NP 083340.1\| | RIKEN cDNA 4930509O20 [Mus musculus] dbj\|BAB29950.1\| unnamed protein product [Mus musculus] | 40 | 0,02 | |
| | | emb\|CAE65034.1\| | Hypothetical protein CBG09874 [Caenorhabditis briggsae] | 40 | 0,02 | |
| | | sp\|P38584\|TTL BOVIN | Tubulin--tyrosine ligase (TTL) | 40 | 0,026 | |
| 1 Zoo I G11 87 13 | 495 | emb\|CAA30852.1\| | alpha tubulin [Gallus gallus] | 269 | 2,00E-71 | cytoscelet |
| | | sp\|P09644\|TBA5 CHICK | TUBULIN ALPHA-5 CHAIN | 269 | 2,00E-71 | |
| | | pir\|\|UBCHA5 | tubulin alpha-5 chain - chicken | 269 | 2,00E-71 | |
| | | gb\|AAM73990.1\| | alpha-tubulin 3 [Strongylocentrotus droebechiensis] | 269 | 2,00E-71 | |
| | | gb\|AAC97929.1\| | alpha tubulin [Notothenia coriiceps] gb\|AAG15324.1\| alpha tubulin [Notothenia coriiceps] | 268 | 3,00E-71 | |
| | | sp\|P18258\|TBA1 PARLI | TUBULIN ALPHA-1 CHAIN pir\|\|S11207 tubulin alpha chain - sea urchin (Paracentrotus lividus) emb\|CAA37680.1\| unnamed protein product [Paracentrotus lividus] | 268 | 3,00E-71 | |
| | | ref\|XP 147357.1\| | tubulin alpha 6 [Mus musculus] | 268 | 3,00E-71 | |
| | | ref\|NP 033474.1\| | tubulin, alpha 6; tubulin alpha 6 [Mus musculus] sp\|P05216\|TBA6_MOUSE Tubulin alpha-6 chain (Alpha-tubulin 6) pir\|\|I77428 tubulin alpha chain isotype M-alpha-6 - mouse gb\|AAA40503.1\| alpha-tubulin | 268 | 3,00E-71 | |
| | | sp\|P08537\|TBA XENLA | Tubulin alpha chain pir\|\|S00253 tubulin alpha chain - African clawed frog emb\|CAA30094.1\| unnamed protein product [Xenopus laevis] | 268 | 3,00E-71 | |
| | | ref\|NP 116093.1\| | tubulin alpha 6 [Homo sapiens] sp\|Q9BQE3\|TBA6_HUMAN Tubulin alpha-6 chain (Alpha-tubulin 6) gb\|AAH04949.1\| Tubulin alpha 6 [Homo sapiens] gb\|AAH05946.1\| Tubulin alpha 6 [Homo sapiens] gb\|A | 268 | 3,00E-71 | |
| 1 Zoo I H04 32 16 | 487 | ref\|XP 233723.2\| | similar to hypothetical protein FLJ36119 [Rattus norvegicus] | 52 | 4,00E-06 | cytoscelet |
| | | ref\|XP 340624.1\| | tubulin-tyrosine ligase, putative [Trypanosoma brucei] gb\|AAQ15983.1\| tubulin-tyrosine ligase, putative [Trypanosoma brucei] | 47 | 2,00E-04 | |
| | | ref\|XP 195428.2\| | similar to dJ366N23.3 (NYD-TSPG protein) [Mus musculus] | 46 | 3,00E-04 | |
| | | dbj\|BAC85781.1\| | unnamed protein product [Homo sapiens] | 45 | 4,00E-04 | |
| | | ref\|NP 701337.1\| | hypothetical protein [Plasmodium falciparum 3D7] gb\|AAN36061.1\| hypothetical protein [Plasmodium falciparum 3D7] | 44 | 0,001 | |
| | | pir\|\|E88575 | protein ZK1128.6 [imported] - Caenorhabditis elegans | 44 | 0,002 | |
| | | pir\|\|T27699 | hypothetical protein ZK1128.6 - Caenorhabditis elegans | 44 | 0,002 | |
| | | ref\|NP 499251.2\| | tubulin-tyrosine ligase (69.1 kD) (3L221) [Caenorhabditis elegans] emb\|CAA87425.3\| Hypothetical protein ZK1128.6 [Caenorhabditis elegans] | 44 | 0,002 | |
| | | gb\|EAA21855.1\| | Tubulin-tyrosine ligase family, putative [Plasmodium yoelii yoelii] | 42 | 0,004 | |
| | | ref\|XP 396662.1\| | similar to ENSANGP00000021502 [Apis mellifera] | 41 | 0,008 | |
| 1 Zoo I A10 73 02 | 354 | dbj\|BAB88855.1\| | cysteine sulfinic acid decarboxylase [Ciona intestinalis] | 95 | 4,00E-19 | metabolism |
| | | ref\|XP 291059.3\| | similar to cysteine sulfinic acid decarboxylase [Homo sapiens] | 95 | 4,00E-19 | |
| | | ref\|XP 392052.1\| | similar to ENSANGP00000014813 [Apis mellifera] | 93 | 1,00E-18 | |
| | | ref\|NP 659191.1\| | cysteine sulfinic acid decarboxylase [Mus musculus] sp\|Q9DBE0\|CSD_MOUSE Cysteine sulfinic acid decarboxylase (Sulfinoalanine decarboxylase) (Cysteine-sulfinate decarboxylase) dbj\|BAB23747.1\| u | 92 | 2,00E-18 | |
| | | gb\|AAK60398.1\| | cysteine sulfinic acid decarboxylase [Mus musculus] | 92 | 2,00E-18 | |
| | | ref\|XP 345994.1\| | similar to cysteine sulfinic acid decarboxylase [Rattus norvegicus] | 90 | 9,00E-18 | |
| | | gb\|AAH52327.1\| | Unknown (protein for IMAGE:6313667) [Mus musculus] | 90 | 9,00E-18 | |
| | | ref\|XP 135211.2\| | RIKEN cDNA 1110027M19 [Mus musculus] | 90 | 9,00E-18 | |
| | | ref\|NP 068518.1\| | cysteine-sulfinate decarboxylase [Rattus norvegicus] sp\|Q64611\|CSD_RAT Cysteine sulfinic acid decarboxylase (Sulfinoalanine decarboxylase) (Cysteine-sulfinate decarboxylase) pir\|\|S71489 sulfinoalani | 89 | 2,00E-17 | |
| | | gb\|AAC42063.1\| | cysteine sulfinic acid decarboxylase prf\|\|2113203A Cys sulfinic acid decarboxylase | 89 | 2,00E-17 | |
| 1 Zoo I C07 51 05 | 395 | ref\|NP 612071.1\| | CG13904-PA [Drosophila melanogaster] gb\|AAF47429.1\| CG13904-PA [Drosophila melanogaster] | 107 | 5,00E-23 | metabolism |
| | | gb\|AAL90165.1\| | AT24870p [Drosophila melanogaster] | 105 | 3,00E-22 | |
| | | ref\|NP 608720.1\| | CG18558-PA [Drosophila melanogaster] gb\|AAF51196.2\| CG18558-PA [Drosophila melanogaster] | 105 | 3,00E-22 | |
| | | emb\|CAE71479.1\| | Hypothetical protein CBG18398 [Caenorhabditis briggsae] | 104 | 3,00E-22 | |
| | | pir\|\|T19837 | hypothetical protein C38H2.2 - Caenorhabditis elegans | 103 | 1,00E-21 | |
| | | ref\|NP 499293.2\| | core1 UDP-galactose:N-acetylgalactosamine-alpha-R beta1,3-galactosyltransferase (44.1 kD) (3L473) [Caenorhabditis elegans] gb\|AAG36940.1\| core1 UDP-galactose:N-acetylgalactosamine-alpha-R bet | 103 | 1,00E-21 | |
| | | gb\|AAH03174.1\| | Unknown (protein for IMAGE:2957836) [Homo sapiens] | 95 | 3,00E-19 | |
| | | ref\|NP 724653.1\| | CG8708-PB [Drosophila melanogaster] gb\|AAM68854.1\| CG8708-PB [Drosophila melanogaster] gb\|AAN71398.1\| RE41083p [Drosophila melanogaster] | 95 | 3,00E-19 | |
| | | ref\|NP 064541.1\| | core1 UDP-galactose:N-acetylgalactosamine-alpha-R beta 1,3-galac [Homo sapiens] gb\|AAF81981.1\| core1 UDP-galactose:N-acetylgalactosamine-alpha-R beta 1,3-galactosyltransferase [Homo sapiens] | 95 | 3,00E-19 | |
| | | emb\|CAC45046.1\| | beta-1,3-galactosyltransferase b3Gal-T8 [Homo sapiens] | 95 | 3,00E-19 | |

Additional file 3: Subtraction library BLAST results top 10. Engine: NCBI. Database: NR.

| Clone | bp | ID | Homology | Score | E value | category |
|---|---|---|---|---|---|---|
| 1_Zoo_I_E10_77_10 | 552 | ref\|XP_233723.2\| | similar to hypothetical protein FLJ36119 [Rattus norvegicus] | 54 | 2,00E-06 | metabolism |
| | | ref\|XP_340624.1\| | tubulin-tyrosine ligase, putative [Trypanosoma brucei] gb\|AAQ15983.1\| tubulin-tyrosine ligase, putative [Trypanosoma brucei] | 47 | 2,00E-04 | |
| | | dbj\|BAC85781.1\| | unnamed protein product [Homo sapiens] | 45 | 6,00E-04 | |
| | | ref\|NP_701337.1\| | hypothetical protein [Plasmodium falciparum 3D7] gb\|AAN36061.1\| hypothetical protein [Plasmodium falciparum 3D7] | 45 | 8,00E-04 | |
| | | ref\|XP_195428.2\| | similar to dJ366N23.3 (NYD-TSPG protein) [Mus musculus] | 44 | 0,001 | |
| | | ref\|NP_499251.2\| | tubulin-tyrosine ligase (69.1 kD) (3L221) [Caenorhabditis elegans] emb\|CAA87425.3\| Hypothetical protein ZK1128.6 [Caenorhabditis elegans] | 44 | 0,002 | |
| | | pir\|\|T27699 | hypothetical protein ZK1128.6 - Caenorhabditis elegans | 44 | 0,002 | |
| | | pir\|\|E88575 | protein ZK1128.6 [imported] - Caenorhabditis elegans | 44 | 0,002 | |
| | | gb\|EAA21855.1\| | Tubulin-tyrosine ligase family, putative [Plasmodium yoelii yoelii] | 44 | 0,002 | |
| | | ref\|XP_217869.2\| | similar to dJ366N23.3 (NYD-TSPG protein) [Rattus norvegicus] | 42 | 0,007 | |
| 1_Zoo_I_B11_82_03 | 365 | gb\|EAL19461.1\| | hypothetical protein CNBG4080 [Cryptococcus neoformans var. neoformans B-3501A] | 39 | 0,018 | metabolism |
| | | gb\|EAA54899.1\| | hypothetical protein MG05690.4 [Magnaporthe grisea 70-15] | 37 | 0,091 | |
| | | ref\|XP_322395.1\| | hypothetical protein [Neurospora crassa] gb\|EAA28544.1\| hypothetical protein [Neurospora crassa] | 37 | 0,12 | |
| | | gb\|EAL04370.1\| | hypothetical protein CaO19.5867 [Candida albicans SC5314] | 37 | 0,12 | |
| | | gb\|EAL04216.1\| | hypothetical protein CaO19.13289 [Candida albicans SC5314] | 37 | 0,12 | |
| | | ref\|NP_595526.1\| | hypothetical protein [Schizosaccharomyces pombe] emb\|CAB09116.1\| transmembrane receptor activity (predicted); serine-rich protein; WSC domain; carbohydrate binding (predicted); proteophosphoglyc | 35 | 0,35 | |
| | | sp\|P87179\|YB1E_SCHP | Serine-rich protein C30B4.01c precursor emb\|CAA20314.2\| SPBC30B4.01c [Schizosaccharomyces pombe] | 35 | 0,35 | |
| | | pir\|\|T40167 | hypothetical protein SPBC30B4.01c - fission yeast (Schizosaccharomyces pombe) (fragment) | 35 | 0,35 | |
| | | ref\|NP_014536.1\| | cell wall integrity and stress response component 3; Wsc3p [Saccharomyces cerevisiae] sp\|Q12215\|WSC3_YEAST Cell wall integrity and stress response component 3 precursor pir\|\|S51892 probable me | 34 | 0,59 | |
| | | emb\|CAG84051.1\| | unnamed protein product [Yarrowia lipolytica] | 33 | 1 | |
| 1_Zoo_I_C02_11_06 | 543 | gb\|EAA39251.1\| | GLP_457_13116_11626 [Giardia lamblia ATCC 50803] | 44 | 0,002 | metabolism |
| | | ref\|NP_965450.1\| | hypothetical protein LJ1643 [Lactobacillus johnsonii NCC 533] gb\|AAS09416.1\| hypothetical protein LJ1643 [Lactobacillus johnsonii NCC 533] | 37 | 0,16 | |
| | | emb\|CAH03336.1\| | Calcium transporting ATPase, putative [Paramecium tetraurelia] | 33 | 2,4 | |
| | | ref\|NP_705044.1\| | hypothetical protein [Plasmodium falciparum 3D7] emb\|CAD52279.1\| hypothetical protein [Plasmodium falciparum 3D7] | 33 | 4 | |
| | | dbj\|BAC39011.1\| | unnamed protein product [Mus musculus] | 32 | 5,3 | |
| | | dbj\|BAA76856.2\| | KIAA1012 protein [Homo sapiens] | 32 | 5,3 | |
| | | emb\|CAG08186.1\| | unnamed protein product [Tetraodon nigroviridis] | 32 | 5,3 | |
| | | gb\|AAH72296.1\| | Unknown (protein for MGC:82500) [Xenopus laevis] | 32 | 5,3 | |
| | | sp\|Q9Y2L5\|TR85_HUMA | TRS85 homolog | 32 | 5,3 | |
| | | ref\|NP_055754.2\| | KIAA1012 [Homo sapiens] | 32 | 5,3 | |
| 1_Zoo_II_A06_137_02 | 613 | gi\|6708335\|gb\| | putative secreted protein [Ixodes sca 64.3 | 64 | 5,00E-09 | metabolism |
| | | ref\|XP_321129.1\| | ENSANGP00000007905 [Anopheles gambiae] gb\|EAA00963.2\| ENSANGP00000007905 [Anopheles gambiae str. PEST] | 39 | 0,075 | n |
| | | ref\|XP_372634.1\| | similar to double homeobox protein [Homo sapiens] | 33 | 0,14 | |
| | | pir\|\|S12206 | hypothetical protein 2 (rRNA external transcribed spacer) - mouse | 36 | 0,63 | |
| | | ref\|NP_848515.1\| | late envelope protein 9 [Homo sapiens] | 35 | 1,1 | |
| | | ref\|XP_342070.1\| | similar to RIKEN cDNA 2610009I02 [Rattus norvegicus] | 33 | 4,1 | |
| | | emb\|CAG06352.1\| | unnamed protein product [Tetraodon nigroviridis] | 33 | 4,1 | |
| | | ref\|NP_848133.1\| | small proline rich-like 4A; late envelope protein 8 [Homo sapiens] | 33 | 5,4 | |
| | | gb\|AAL87135.1\| | monomeric alpha-macroglobulin [Rana catesbeiana] | 32 | 7 | |
| | | ref\|NP_051961.1\| | gp072L [Rabbit fibroma virus] gb\|AAF17954.1\| gp072L [Rabbit fibroma virus] | 32 | 7 | |
| | | pir\|\|T03095 | homeoprotein Sail - fruit fly (Drosophila melanogaster) gb\|AAB53640.1\| mirror [Drosophila melanogaster] gb\|AAC23943.1\| homeoprotein Sail [Drosophila melanogaster] | 32 | 9,2 | |
| 1_Zoo_I_A05_33_01 | 532 | ref\|NP_496167.1\| | protein phosphatase family member (2K316) [Caenorhabditis elegans] pir\|\|T22522 phosphoprotein phosphatase (EC 3.1.3.16) 1 F52H3.6 [similarity] - Caenorhabditis elegans emb\|CAA91326.1\| Hypotheti | 100 | 2,00E-20 | intracellular signalling |
| | | emb\|CAE67133.1\| | Hypothetical protein CBG12555 [Caenorhabditis briggsae] | 99 | 4,00E-20 | |
| | | gb\|AAK64283.1\| | protein phosphatase [Oryza sativa] dbj\|BAD22973.1\| protein phosphatase [Oryza sativa (japonica cultivar-group)] dbj\|BAD23102.1\| protein phosphatase [Oryza sativa (japonica cultivar-group)] | 99 | 6,00E-20 | |
| | | ref\|NP_568625.1\| | serine/threonine protein phosphatase PP1 isozyme 7 (TOPP7) [Arabidopsis thaliana] dbj\|BAA24283.1\| protein phosphatase 1 catalytic subunit [Arabidopsis thaliana] dbj\|BAA97417.1\| protein phosphatase | 99 | 6,00E-20 | |
| | | ref\|NP_851123.1\| | serine/threonine protein phosphatase PP1 isozyme 7 (TOPP7) [Arabidopsis thaliana] sp\|O82733\|PP17_ARATH Serine/threonine protein phosphatase PP1 isozyme 7 gb\|AAM97129.1\| expressed protein [A | 99 | 6,00E-20 | |
| | | ref\|NP_187209.1\| | serine/threonine protein phosphatase, putative [Arabidopsis thaliana] gb\|AAF26139.1\| putative serine/threonine protein phosphatase type one [Arabidopsis thaliana] gb\|AAQ65155.1\| At3g05580 [Arabidops | 99 | 6,00E-20 | |
| | | sp\|O04857\|PP12_TOBA | Serine/threonine protein phosphatase PP1 isozyme 2 pir\|\|T03596 phosphoprotein phosphatase (EC 3.1.3.16) 1 - common tobacco emb\|CAB07804.1\| protein phosphatase type 1 [Nicotiana tabacum] | 98 | 1,00E-19 | |
| | | sp\|P22198\|PP1_MAIZE | Serine/threonine protein phosphatase PP1 pir\|\|S29317 phosphoprotein phosphatase (EC 3.1.3.16) 1 - maize gb\|AAA33545.1\| protein phosphatase-1 prf\|\|1909338A protein phosphatase 1 | 97 | 1,00E-19 | |
| | | pir\|\|T09550 | phosphoprotein phosphatase (EC 3.1.3.16) 1, catalytic epsilon chain - alfalfa emb\|CAA05494.1\| protein phosphatase 1, catalytic epsilon subunit [Medicago sativa] | 97 | 1,00E-19 | |
| | | ref\|NP_908906.1\| | putative serine/threonine protein phosphatase [Oryza sativa (japonica cultivar-group)] dbj\|BAB93408.1\| putative serine/threonine protein phosphatase [Oryza sativa (japonica cultivar-group)] | 97 | 1,00E-19 | |
| 1_Zoo_I_B05_34_03 | 470 | ref\|XP_404547.1\| | PP1_EMENI SERINE/THREONINE PROTEIN PHOSPHATASE PP1 [Aspergillus nidulans FGSC A4] sp\|P20654\|PP1_EMENI Serine/threonine protein phosphatase PP1 pir\|\|A32549 phosphoprotein phos | 55 | 4,00E-07 | intracellular signalling |
| | | ref\|XP_327775.1\| | hypothetical protein ( (AF071751) protein phosphatase-Z-like serine/threonine protein phosphatase [Neurospora crassa] ) gb\|EAA35800.1\| hypothetical protein ( (AF071751) protein phosphatase-Z-like se | 55 | 4,00E-07 | |
| | | gb\|AAD09995.1\| | protein phosphatase-Z-like serine/threonine protein phosphatase [Neurospora crassa] gb\|AAD09996.1\| protein phosphatase-Z-like serine/threonine protein phosphatase [Neurospora crassa] | 55 | 4,00E-07 | |
| | | ref\|NP_505733.1\| | yeast Glc Seven-like Phosphatase (37.2 kD) (gsp-1) [Caenorhabditis elegans] pir\|\|T21553 phosphoprotein phosphatase (EC 3.1.3.16) 1-beta F29F11.6 [similarity] - Caenorhabditis elegans emb\|CAA9827 | 55 | 5,00E-07 | |
| | | gb\|EAA48491.1\| | hypothetical protein MG00149.4 [Magnaporthe grisea 70-15] | 55 | 5,00E-07 | |
| | | gb\|EAL22523.1\| | hypothetical protein CNBB4010 [Cryptococcus neoformans var. neoformans B-3501A] | 55 | 7,00E-07 | |
| | | gb\|EAA57520.1\| | hypothetical protein MG10195.4 [Magnaporthe grisea 70-15] | 55 | 7,00E-07 | |
| | | sp\|P48727\|YME1_CAEE | Putative serine/threonine protein phosphatase F56C9.1 in chromosome III pir\|\|T16476 phosphoprotein phosphatase (EC 3.1.3.16) 1-alpha F56C9.1 [similarity] - Caenorhabditis elegans gb\|AAK18957.1\| H | 54 | 9,00E-07 | |
| | | gb\|AAR88564.1\| | AT31252p [Drosophila melanogaster] | 54 | 1,00E-06 | |
| | | ref\|NP_524947.1\| | CG8822-PA [Drosophila melanogaster] gb\|AAF51146.1\| CG8822-PA [Drosophila melanogaster] | 54 | 1,00E-06 | |

Additional file 3: Subtraction library BLAST results top 10. Engine: NCBI. Database: NR.

| Clone | bp | ID | Homology | Score | E value | category |
|---|---|---|---|---|---|---|
| 1_Zoo_I_C10_75_06 | 525 | ref\|NP_193170.1\| | casein kinase I (CKI1) [Arabidopsis thaliana] pir\|\|C71405 probable casein kinase I - Arabidopsis thaliana emb\|CAA55395.1\| casein kinase I [Arabidopsis thaliana] emb\|CAB10213.1\| casein kinase I [Arabid | 90 | 2,00E-17 | intracellular signalling |
| | | emb\|CAE63293.1\| | Hypothetical protein CBG07674 [Caenorhabditis briggsae] | 87 | 1,00E-16 | |
| | | gb\|AAP47012.1\| | casein kinase I epsilon [Gallus gallus] | 87 | 1,00E-16 | |
| | | dbj\|BAB32922.1\| | casein kinase1 epsilon-2 [Rattus norvegicus] | 87 | 1,00E-16 | |
| | | dbj\|BAB03473.1\| | casein kinase 1 epsilon-3 [Rattus norvegicus] | 87 | 1,00E-16 | |
| | | ref\|NP_955877.1\| | casein kinase 1, delta; wu:fb50c04 [Danio rerio] gb\|AAH54583.1\| Casein kinase 1, delta [Danio rerio] | 87 | 1,00E-16 | |
| | | pdb\|1CKJ\|A | Chain A, Casein Kinase I Delta Truncation Mutant Containing Residues 1 - 317 Complex With Bound Tungstate pdb\|1CKJ\|B Chain B, Casein Kinase I Delta Truncation Mutant Containing Residues 1 - 31 | 87 | 1,00E-16 | |
| | | emb\|CAG05944.1\| | unnamed protein product [Tetraodon nigroviridis] | 87 | 1,00E-16 | |
| | | ref\|NP_989089.1\| | hypothetical protein MGC75636 [Xenopus tropicalis] gb\|AAH62487.1\| Hypothetical protein MGC75636 [Xenopus tropicalis] | 87 | 1,00E-16 | |
| | | ref\|NP_001884.2\| | casein kinase 1, delta isoform 1 [Homo sapiens] sp\|P48730\|KC1D_HUMAN Casein kinase I, delta isoform (CKI-delta) (CKId) gb\|AAH03558.1\| Casein kinase 1, delta, isoform 1 [Homo sapiens] dbj\|BAC10 | 87 | 1,00E-16 | |
| 1_Zoo_I_C11_83_05 | 547 | ref\|NP_193170.1\| | casein kinase I (CKI1) [Arabidopsis thaliana] pir\|\|C71405 probable casein kinase I - Arabidopsis thaliana emb\|CAA55395.1\| casein kinase I [Arabidopsis thaliana] emb\|CAB10213.1\| casein kinase I [Arabid | 82 | 8,00E-15 | intracellular signalling |
| | | emb\|CAA55396.1\| | casein kinase I [Arabidopsis thaliana] | 77 | 2,00E-13 | |
| | | ref\|NP_188976.1\| | casein kinase, putative [Arabidopsis thaliana] dbj\|BAB02278.1\| casein kinase [Arabidopsis thaliana] gb\|AAL06840.1\| AT3g23340/MLM24_21 [Arabidopsis thaliana] gb\|AAL67096.1\| AT3g23340/MLM24_21 | 77 | 2,00E-13 | |
| | | dbj\|BAD21585.1\| | putative casein kinase I [Oryza sativa (japonica cultivar-group)] | 76 | 3,00E-13 | |
| | | pir\|\|E86176 | protein F19P19.10 [imported] - Arabidopsis thaliana gb\|AAB70431.1\| F19P19.10 [Arabidopsis thaliana] | 75 | 6,00E-13 | |
| | | ref\|NP_171939.1\| | casein kinase, putative [Arabidopsis thaliana] | 75 | 6,00E-13 | |
| | | gb\|AAO22771.1\| | putative casein kinase I [Arabidopsis thaliana] | 75 | 7,00E-13 | |
| | | ref\|NP_563695.2\| | protein kinase (ADK1) [Arabidopsis thaliana] pir\|B86170 ADK1 [imported] - Arabidopsis thaliana gb\|AAD10678.1\| ADK1 [Arabidopsis thaliana] gb\|AAL38850.1\| putative protein kinase ADK1 [Arabidopsis t | 74 | 1,00E-12 | |
| | | pir\|\|A55661 | protein kinase ADK1 - Arabidopsis thaliana gb\|AAB47968.1\| dual specificity kinase 1 | 74 | 1,00E-12 | |
| | | ref\|XP_395574.1\| | similar to Casein kinase 1, delta [Apis mellifera] | 74 | 2,00E-12 | |
| 1_Zoo_I_C12_91_06 | 545 | ref\|NP_193170.1\| | casein kinase I (CKI1) [Arabidopsis thaliana] pir\|\|C71405 probable casein kinase I - Arabidopsis thaliana emb\|CAA55395.1\| casein kinase I [Arabidopsis thaliana] emb\|CAB10213.1\| casein kinase I [Arabid | 84 | 2,00E-15 | intracellular signalling |
| | | emb\|CAA55396.1\| | casein kinase I [Arabidopsis thaliana] | 79 | 5,00E-14 | |
| | | ref\|NP_188976.1\| | casein kinase, putative [Arabidopsis thaliana] dbj\|BAB02278.1\| casein kinase [Arabidopsis thaliana] gb\|AAL06840.1\| AT3g23340/MLM24_21 [Arabidopsis thaliana] gb\|AAL67096.1\| AT3g23340/MLM24_21 | 79 | 5,00E-14 | |
| | | dbj\|BAD21585.1\| | putative casein kinase I [Oryza sativa (japonica cultivar-group)] | 78 | 9,00E-14 | |
| | | ref\|NP_171939.1\| | casein kinase, putative [Arabidopsis thaliana] | 77 | 1,00E-13 | |
| | | pir\|\|E86176 | protein F19P19.10 [imported] - Arabidopsis thaliana gb\|AAB70431.1\| F19P19.10 [Arabidopsis thaliana] | 77 | 1,00E-13 | |
| | | gb\|AAO22771.1\| | putative casein kinase I [Arabidopsis thaliana] | 77 | 2,00E-13 | |
| | | ref\|NP_199223.1\| | casein kinase, putative [Arabidopsis thaliana] dbj\|BAB10977.1\| casein kinase I [Arabidopsis thaliana] | 75 | 6,00E-13 | |
| | | ref\|NP_524602.1\| | CG2048-PC [Drosophila melanogaster] ref\|NP_733414.1\| CG2048-PA [Drosophila melanogaster] ref\|NP_733415.1\| CG2048-PB [Drosophila melanogaster] gb\|AAD27857.1\| double-time [Drosophila mela | 75 | 7,00E-13 | |
| | | sp\|O76324\|DCO_DROM | Discs overgrown protein kinase (Double-time protein) gb\|AAC39134.1\| casein kinase I homolog [Drosophila melanogaster] | 75 | 7,00E-13 | |
| 1_Zoo_I_D02_12_08 | 540 | ref\|XP_306392.1\| | ENSANGP00000000059 [Anopheles gambiae] gb\|EAA01910.1\| ENSANGP00000000059 [Anopheles gambiae str. PEST] | 95 | 9,00E-19 | intracellular signalling |
| | | ref\|XP_317893.1\| | ENSANGP00000024066 [Anopheles gambiae] gb\|EAA43798.1\| ENSANGP00000024066 [Anopheles gambiae str. PEST] | 95 | 9,00E-19 | |
| | | ref\|XP_317894.1\| | ENSANGP00000004936 [Anopheles gambiae] gb\|EAA12933.1\| ENSANGP00000004936 [Anopheles gambiae str. PEST] | 95 | 9,00E-19 | |
| | | ref\|XP_223273.2\| | similar to protein phosphatase with EF-hands-2 [Rattus norvegicus] | 91 | 2,00E-17 | |
| | | ref\|NP_035278.1\| | serine/threonine protein phosphatase with EF-hand motifs 2 [Mus musculus] sp\|O35385\|PPE2_MOUSE Serine/threonine protein phosphatase with EF-hands-2 (PPEF-2) gb\|AAB82798.1\| protein phospha | 91 | 2,00E-17 | |
| | | ref\|NP_006230.2\| | serine/threonine protein phosphatase with EF-hand motifs 2 isoform a; protein phosphatase with EF hands 2 [Homo sapiens] | 90 | 2,00E-17 | |
| | | sp\|O14830\|PPE2_HUMA | Serine/threonine protein phosphatase with EF-hands-2 (PPEF-2) gb\|AAB82796.1\| protein phosphatase with EF-hands-2 long form [Homo sapiens] | 90 | 2,00E-17 | |
| | | emb\|CAE69136.1\| | Hypothetical protein CBG15166 [Caenorhabditis briggsae] | 89 | 4,00E-17 | |
| | | ref\|NP_741091.1\| | protein Phosphatase with EF hands, homologous to Drosophila retinal degeneration C (80.3 kD) (pef-1) [Caenorhabditis elegans] pir\|\|T42239 probable phosphoprotein phosphatase (EC 3.1.3.16) - Caeno | 89 | 6,00E-17 | |
| | | gb\|AAM22065.1\| | Phosphatase with ef hands protein 1, isoform b [Caenorhabditis elegans] | 89 | 6,00E-17 | |
| 1_Zoo_I_D10_76_08 | 446 | ref\|NP_596317.1\| | serine-threonine protein phosphatase pp1-1 [Schizosaccharomyces pombe] sp\|P13681\|PP11_SCHPO Serine/threonine protein phosphatase PP1-1 pir\|\|A32550 phosphoprotein phosphatase (EC 3.1.3.16 | 63 | 1,00E-09 | intracellular signalling |
| | | sp\|Q9UW86\|PP1_NEUC | Serine/threonine protein phosphatase PP1 gb\|AAD47567.1\| protein phosphatase-1; PPP1 [Neurospora crassa] | 62 | 2,00E-09 | |
| | | gb\|AAC05275.1\| | serine/threonine protein phosphatase type 1 [Neurospora crassa] | 62 | 2,00E-09 | |
| | | ref\|XP_387409.1\| | PP1_NEUCR Serine/threonine protein phosphatase PP1 [Gibberella zeae PH-1] gb\|EAA77831.1\| PP1_NEUCR Serine/threonine protein phosphatase PP1 [Gibberella zeae PH-1] | 62 | 2,00E-09 | |
| | | ref\|XP_322129.1\| | SERINE/THREONINE PROTEIN PHOSPHATASE PP1 [Neurospora crassa] gb\|EAA26918.1\| SERINE/THREONINE PROTEIN PHOSPHATASE PP1 [Neurospora crassa] | 62 | 2,00E-09 | |
| | | gb\|AAT52055.1\| | protein phosphatase 1 alpha [Drosophila buzzatii] | 62 | 2,00E-09 | |
| | | gb\|EAA48491.1\| | hypothetical protein MG00149.4 [Magnaporthe grisea 70-15] | 62 | 2,00E-09 | |
| | | pir\|\|S29310 | phosphoprotein phosphatase (EC 3.1.3.16) - Paramecium tetraurelia gb\|AAA19173.1\| phosphoprotein phosphatase 1 emb\|CAA47831.1\| serine /threonine specific protein phosphatase [Paramecium tetrau | 62 | 2,00E-09 | |
| | | gb\|AAA19174.1\| | phosphoprotein phosphatase 1 | 62 | 2,00E-09 | |
| | | ref\|XP_402442.1\| | hypothetical protein UM04827.1 [Ustilago maydis 521] gb\|EAK86282.1\| hypothetical protein UM04827.1 [Ustilago maydis 521] | 62 | 3,00E-09 | |
| 1_Zoo_I_E02_13_10 | 567 | gb\|EAA48491.1\| | hypothetical protein MG00149.4 [Magnaporthe grisea 70-15] | 87 | 2,00E-16 | intracellular signalling |
| | | gb\|AAR88564.1\| | AT31252p [Drosophila melanogaster] | 87 | 2,00E-16 | |
| | | ref\|NP_524947.1\| | CG8822-PA [Drosophila melanogaster] gb\|AAF51146.1\| CG8822-PA [Drosophila melanogaster] | 87 | 2,00E-16 | |
| | | ref\|XP_327775.1\| | hypothetical protein ( (AF071751) protein phosphatase-Z-like serine/threonine protein phosphatase [Neurospora crassa] ) gb\|EAA35800.1\| hypothetical protein ( (AF071751) protein phosphatase-Z-like se | 86 | 3,00E-16 | |
| | | ref\|XP_404547.1\| | PP1_EMENI SERINE/THREONINE PROTEIN PHOSPHATASE PP1 [Aspergillus nidulans FGSC A4] sp\|P20654\|PP1_EMENI Serine/threonine protein phosphatase PP1 pir\|\|A32549 phosphoprotein phos | 86 | 6,00E-16 | |
| | | sp\|P48727\|YME1_CAEE | Putative serine/threonine protein phosphatase F56C9.1 in chromosome III pir\|\|T16476 phosphoprotein phosphatase (EC 3.1.3.16) 1-alpha F56C9.1 [similarity] - Caenorhabditis elegans gb\|AAK18957.1\| H | 86 | 6,00E-16 | |
| | | ref\|NP_505733.1\| | yeast Glc Seven-like Phosphatase (37.2 kD) (gsp-1) [Caenorhabditis elegans] pir\|\|T21553 phosphoprotein phosphatase (EC 3.1.3.16) 1-beta F29F11.6 [similarity] - Caenorhabditis elegans emb\|CAA9827 | 85 | 8,00E-16 | |
| | | emb\|CAE57617.1\| | Hypothetical protein CBG00598 [Caenorhabditis briggsae] | 85 | 8,00E-16 | |
| | | gb\|EAA57520.1\| | hypothetical protein MG10195.4 [Magnaporthe grisea 70-15] | 85 | 1,00E-15 | |
| | | pir\|\|S29310 | phosphoprotein phosphatase (EC 3.1.3.16) - Paramecium tetraurelia gb\|AAA19173.1\| phosphoprotein phosphatase 1 emb\|CAA47831.1\| serine /threonine specific protein phosphatase [Paramecium tetrau | 84 | 1,00E-15 | |

Additional file 3: Subtraction library BLAST results top 10. Engine: NCBI. Database: NR.

| Clone | bp | ID | Homology | Score | E value | category |
|---|---|---|---|---|---|---|
| 1 Zoo I E03 21 09 | 555 | emb\|CAE01426.2\| | protein kinase A catalytic subunit 2 [Aspergillus fumigatus] | 85 | 1,00E-15 | intracellular signalling |
| | | ref\|XP 408854.1\| | hypothetical protein AN4717.2 [Aspergillus nidulans FGSC A4] gb\|EAA60759.1\| hypothetical protein AN4717.2 [Aspergillus nidulans FGSC A4] | 80 | 2,00E-14 | |
| | | gb\|AAA57318.1\| | serine/threonine protein kinase | 73 | 3,00E-12 | |
| | | ref\|XP 402071.1\| | hypothetical protein UM04456.1 [Ustilago maydis 521] gb\|AAA75366.1\| vinclozolin resistance protein gb\|EAK85724.1\| hypothetical protein UM04456.1 [Ustilago maydis 521] | 73 | 3,00E-12 | |
| | | ref\|XP 449711.1\| | unnamed protein product [Candida glabrata] emb\|CAG62687.1\| unnamed protein product [Candida glabrata] | 72 | 6,00E-12 | |
| | | gb\|EAA38644.1\| | GLP_59_15138_16382 [Giardia lamblia ATCC 50803] | 72 | 6,00E-12 | |
| | | sp\|P54644\|KRAC DICDI | RAC-family serine/threonine-protein kinase homolog gb\|AAA76692.1\| rac-alpha serine/threonine kinase homolog | 72 | 6,00E-12 | |
| | | gb\|AAK01549.1\| | cAMP-dependent protein kinase catalytic subunit [Toxoplasma gondii] | 71 | 1,00E-11 | |
| | | ref\|NP 005391.1\| | protein kinase C, epsilon [Homo sapiens] sp\|Q02156\|KPCE_HUMAN Protein kinase C, epsilon type (nPKC-epsilon) pir\|\|S28942 protein kinase C (EC 2.7.1.-) epsilon - human emb\|CAA46388.1\| protein kin | 70 | 2,00E-11 | |
| | | emb\|CAH03534.1\| | Putative kinase, similar to Sgk1 serine/threonine-protein kinase [Paramecium tetraurelia] | 70 | 2,00E-11 | |
| 1 Zoo I E04 29 10 | 398 | ref\|NP 496167.1\| | protein phosphatase family member (2K316) [Caenorhabditis elegans] pir\|\|T22522 phosphoprotein phosphatase (EC 3.1.3.16) 1 F52H3.6 [similarity] - Caenorhabditis elegans emb\|CAA91326.1\| Hypotheti | 100 | 6,00E-21 | intracellular signalling |
| | | ref\|NP 187209.1\| | serine/threonine protein phosphatase, putative [Arabidopsis thaliana] gb\|AAF26139.1\| putative serine/threonine protein phosphatase type one [Arabidopsis thaliana] gb\|AAQ65155.1\| At3g05580 [Arabidops | 99 | 2,00E-20 | |
| | | emb\|CAE67133.1\| | Hypothetical protein CBG12555 [Caenorhabditis briggsae] | 99 | 2,00E-20 | |
| | | ref\|NP 568625.1\| | serine/threonine protein phosphatase PP1 isozyme 7 (TOPP7) [Arabidopsis thaliana] dbj\|BAA24283.1\| protein phosphatase 1 catalytic subunit [Arabidopsis thaliana] dbj\|BAA97417.1\| protein phosphatase | 99 | 2,00E-20 | |
| | | gb\|AAK64283.1\| | protein phosphatase [Oryza sativa] dbj\|BAD22973.1\| protein phosphatase [Oryza sativa (japonica cultivar-group)] dbj\|BAD23102.1\| protein phosphatase [Oryza sativa (japonica cultivar-group)] | 99 | 2,00E-20 | |
| | | ref\|NP 851123.1\| | serine/threonine protein phosphatase PP1 isozyme 7 (TOPP7) [Arabidopsis thaliana] sp\|O82733\|PP17_ARATH Serine/threonine protein phosphatase PP1 isozyme 7 gb\|AAM97129.1\| expressed protein [A | 99 | 2,00E-20 | |
| | | pir\|\|T09544 | phosphoprotein phosphatase (EC 3.1.3.16), catalytic beta chain - alfalfa emb\|CAA05491.1\| protein phosphatase 1, catalytic beta subunit [Medicago sativa] | 98 | 3,00E-20 | |
| | | pir\|\|T09550 | phosphoprotein phosphatase (EC 3.1.3.16) 1, catalytic epsilon chain - alfalfa emb\|CAA05494.1\| protein phosphatase 1, catalytic epsilon subunit [Medicago sativa] | 98 | 3,00E-20 | |
| | | sp\|O04857\|PP12 TOBA | Serine/threonine protein phosphatase PP1 isozyme 2 pir\|\|T03596 phosphoprotein phosphatase (EC 3.1.3.16) 1 - common tobacco emb\|CAB07804.1\| protein phosphatase type 1 [Nicotiana tabacum] | 98 | 4,00E-20 | |
| | | ref\|NP 908906.1\| | putative serine/threonine protein phosphatase [Oryza sativa (japonica cultivar-group)] dbj\|BAB93408.1\| putative serine/threonine protein phosphatase [Oryza sativa (japonica cultivar-group)] | 97 | 5,00E-20 | |
| 1 Zoo I G02 15 14 | 489 | emb\|CAG03461.1\| | unnamed protein product [Tetraodon nigroviridis] | 151 | 4,00E-36 | intracellular signalling |
| | | ref\|XP 317423.1\| | ENSANGP00000011546 [Anopheles gambiae] gb\|EAA12230.1\| ENSANGP00000011546 [Anopheles gambiae str. PEST] | 149 | 2,00E-35 | |
| | | emb\|CAG01116.1\| | unnamed protein product [Tetraodon nigroviridis] | 149 | 2,00E-35 | |
| | | gb\|AAQ81631.1\| | protein kinase A [Rattus norvegicus] | 149 | 2,00E-35 | |
| | | sp\|Q8MJ44\|KAPA CANI | cAMP-dependent protein kinase, alpha-catalytic subunit (PKA C-alpha) gb\|AAM88381.1\| protein kinase A alpha [Canis familiaris] | 149 | 2,00E-35 | |
| | | pdb\|1CTP\|E | Chain E, Camp-Dependent Protein Kinase (E.C.2.7.1.37) (Capk) (Catalytic Subunit) | 149 | 2,00E-35 | |
| | | pdb\|2CPK\|E | Chain E, c-AMP-Dependent Protein Kinase (E.C.2.7.1.37) (cAPK) (Catalytic Subunit) pdb\|1ATP\|E Chain E, c-AMP-Dependent Protein Kinase (E.C.2.7.1.37) (cAPK) (Catalytic Subunit) Complex With The I | 149 | 2,00E-35 | |
| | | pdb\|1CMK\|E | Chain E, Camp-Dependent Protein Kinase Catalytic Subunit (E.C.2.7.1.37) pdb\|1CDK\|A Chain A, Camp-Dependent Protein Kinase Catalytic Subunit (E.C.2.7.1.37) (Protein Kinase A) Complexed With Pro | 149 | 2,00E-35 | |
| | | ref\|NP 997401.1\| | cAMP-dependent protein kinase catalytic subunit alpha isoform 2; protein kinase A catalytic subunit; PKA C-alpha [Homo sapiens] | 149 | 2,00E-35 | |
| | | gb\|AAF76424.1\| | sperm cAMP-dependent protein kinase catalytic subunit Cs [Ovis aries] | 149 | 2,00E-35 | |
| 1 Zoo I H05 40 15 | 402 | ref\|NP 496167.1\| | protein phosphatase family member (2K316) [Caenorhabditis elegans] pir\|\|T22522 phosphoprotein phosphatase (EC 3.1.3.16) 1 F52H3.6 [similarity] - Caenorhabditis elegans emb\|CAA91326.1\| Hypotheti | 100 | 6,00E-21 | intracellular signalling |
| | | emb\|CAE67133.1\| | Hypothetical protein CBG12555 [Caenorhabditis briggsae] | 99 | 2,00E-20 | |
| | | ref\|NP 187209.1\| | serine/threonine protein phosphatase, putative [Arabidopsis thaliana] gb\|AAF26139.1\| putative serine/threonine protein phosphatase type one [Arabidopsis thaliana] gb\|AAQ65155.1\| At3g05580 [Arabidops | 99 | 2,00E-20 | |
| | | ref\|NP 568625.1\| | serine/threonine protein phosphatase PP1 isozyme 7 (TOPP7) [Arabidopsis thaliana] dbj\|BAA24283.1\| protein phosphatase 1 catalytic subunit [Arabidopsis thaliana] dbj\|BAA97417.1\| protein phosphatase | 99 | 2,00E-20 | |
| | | ref\|NP 851123.1\| | serine/threonine protein phosphatase PP1 isozyme 7 (TOPP7) [Arabidopsis thaliana] sp\|O82733\|PP17_ARATH Serine/threonine protein phosphatase PP1 isozyme 7 gb\|AAM97129.1\| expressed protein [A | 99 | 2,00E-20 | |
| | | gb\|AAK64283.1\| | protein phosphatase [Oryza sativa] dbj\|BAD22973.1\| protein phosphatase [Oryza sativa (japonica cultivar-group)] dbj\|BAD23102.1\| protein phosphatase [Oryza sativa (japonica cultivar-group)] | 99 | 2,00E-20 | |
| | | emb\|CAC85302.1\| | putative serine/threonine protein phosphatase [Trypanosoma cruzi] | 98 | 4,00E-20 | |
| | | sp\|O04857\|PP12 TOBA | Serine/threonine protein phosphatase PP1 isozyme 2 pir\|\|T03596 phosphoprotein phosphatase (EC 3.1.3.16) 1 - common tobacco emb\|CAB07804.1\| protein phosphatase type 1 [Nicotiana tabacum] | 98 | 4,00E-20 | |
| | | pir\|\|T09544 | phosphoprotein phosphatase (EC 3.1.3.16), catalytic beta chain - alfalfa emb\|CAA05491.1\| protein phosphatase 1, catalytic beta subunit [Medicago sativa] | 97 | 5,00E-20 | |
| | | pir\|\|T09550 | phosphoprotein phosphatase (EC 3.1.3.16) 1, catalytic epsilon chain - alfalfa emb\|CAA05494.1\| protein phosphatase 1, catalytic epsilon subunit [Medicago sativa] | 97 | 5,00E-20 | |
| 1 Zoo I H06 48 16 | 552 | ref\|XP 233723.2\| | similar to hypothetical protein FLJ36119 [Rattus norvegicus] | 53 | 4,00E-06 | intracellular signalling |
| | | ref\|NP 701337.1\| | hypothetical protein [Plasmodium falciparum 3D7] gb\|AAN36061.1\| hypothetical protein [Plasmodium falciparum 3D7] | 46 | 4,00E-04 | |
| | | dbj\|BAC85781.1\| | unnamed protein product [Homo sapiens] | 46 | 5,00E-04 | |
| | | ref\|XP 340624.1\| | tubulin-tyrosine ligase, putative [Trypanosoma brucei] gb\|AAQ15983.1\| tubulin-tyrosine ligase, putative [Trypanosoma brucei] | 45 | 6,00E-04 | |
| | | pir\|\|E88575 | protein ZK1128.6 [imported] - Caenorhabditis elegans | 45 | 6,00E-04 | |
| | | ref\|NP 499251.2\| | tubulin-tyrosine ligase (69.1 kD) (3L221) [Caenorhabditis elegans] emb\|CAA87425.3\| Hypothetical protein ZK1128.6 [Caenorhabditis elegans] | 45 | 6,00E-04 | |
| | | pir\|\|T27699 | hypothetical protein ZK1128.6 - Caenorhabditis elegans | 45 | 6,00E-04 | |
| | | gb\|EAA21855.1\| | Tubulin-tyrosine ligase family, putative [Plasmodium yoelii yoelii] | 45 | 0,001 | |
| | | ref\|XP 195428.2\| | similar to dJ366N23.3 (NYD-TSPG protein) [Mus musculus] | 44 | 0,002 | |
| | | emb\|CAG01732.1\| | unnamed protein product [Tetraodon nigroviridis] | 42 | 0,007 | |
| 1 Zoo II A03 113 01 | 481 | pir\|\|T29290 | phosphoprotein phosphatase (EC 3.1.3.16) C34D4.2 [similarity] - Caenorhabditis elegans | 56 | 2,00E-07 | intracellular signalling |
| | | ref\|NP 501125.1\| | protein phosphatase 1 catalytic family member (4H921) [Caenorhabditis elegans] gb\|AAB00704.2\| Hypothetical protein C34D4.2 [Caenorhabditis elegans] | 56 | 2,00E-07 | |
| | | ref\|NP 506574.1\| | protein phosphatase family member (5O909) [Caenorhabditis elegans] pir\|\|T21288 phosphoprotein phosphatase (EC 3.1.3.16) F23B12.1 [similarity] - Caenorhabditis elegans emb\|CAB01164.1\| Hypothetic | 55 | 4,00E-07 | |
| | | emb\|CAE67810.1\| | Hypothetical protein CBG13388 [Caenorhabditis briggsae] | 54 | 1,00E-06 | |
| | | emb\|CAA76756.1\| | serine-threonine protein phosphatase [Drosophila melanogaster] | 53 | 2,00E-06 | |
| | | ref\|NP 477384.1\| | CG3245-PA [Drosophila melanogaster] gb\|AAF46772.1\| CG3245-PA [Drosophila melanogaster] | 53 | 2,00E-06 | |
| | | ref\|NP 727418.1\| | CG2096-PA [Drosophila melanogaster] gb\|AAL39192.1\| GH05039p [Drosophila melanogaster] gb\|AAF46582.2\| CG2096-PA [Drosophila melanogaster] | 53 | 3,00E-06 | |
| | | gb\|AAM11400.1\| | RE17877p [Drosophila melanogaster] | 53 | 3,00E-06 | |
| | | ref\|NP 524738.1\| | CG2096-PB [Drosophila melanogaster] sp\|P48462\|PP1B_DROME Serine/threonine protein phosphatase beta isoform (Flap wing protein) pir\|\|S13828 phosphoprotein phosphatase (EC 3.1.3.16) 1-beta ca | 53 | 3,00E-06 | |
| | | gb\|AAM11075.1\| | GH20565p [Drosophila melanogaster] | 52 | 4,00E-06 | |

Additional file 3: Subtraction library BLAST results top 10. Engine: NCBI. Database: NR.

| Clone | bp | ID | Homology | Score | E value | category |
|---|---|---|---|---|---|---|
| 1 Zoo I E08 61 10 | 605 | ref\|XP 315833.1\| | ENSANGP00000004457 [Anopheles gambiae] gb\|EAA10801.2\| ENSANGP00000004457 [Anopheles gambiae str. PEST] | 60 | 4,00E-08 | other |
| | | gb\|EAL19732.1\| | hypothetical protein CNBG3600 [Cryptococcus neoformans var. neoformans B-3501A] | 59 | 9,00E-08 | |
| | | emb\|CAD11404.1\| | hypothetical protein [Neurospora crassa] | 57 | 2,00E-07 | |
| | | ref\|XP 323510.1\| | hypothetical protein [Neurospora crassa] gb\|EAA31492.1\| hypothetical protein [Neurospora crassa] | 57 | 2,00E-07 | |
| | | ref\|XP 322125.1\| | hypothetical protein [Neurospora crassa] gb\|EAA26914.1\| hypothetical protein [Neurospora crassa] | 57 | 3,00E-07 | |
| | | ref\|XP 390611.1\| | hypothetical protein FG10435.1 [Gibberella zeae PH-1] gb\|EAA68209.1\| hypothetical protein FG10435.1 [Gibberella zeae PH-1] | 55 | 1,00E-06 | |
| | | ref\|NP 647705.1\| | CG32300-PB [Drosophila melanogaster] gb\|AAF47625.1\| CG32300-PB [Drosophila melanogaster] gb\|AAL39805.1\| LD43716p [Drosophila melanogaster] emb\|CAD23246.1\| peptide O-xylosyltransferase [ | 55 | 1,00E-06 | |
| | | ref\|XP 385939.1\| | hypothetical protein FG05763.1 [Gibberella zeae PH-1] gb\|EAA75838.1\| hypothetical protein FG05763.1 [Gibberella zeae PH-1] | 55 | 1,00E-06 | |
| | | ref\|XP 322395.1\| | hypothetical protein [Neurospora crassa] gb\|EAA28544.1\| hypothetical protein [Neurospora crassa] | 54 | 2,00E-06 | |
| | | gb\|EAA50002.1\| | hypothetical protein MG03761.4 [Magnaporthe grisea 70-15] | 54 | 2,00E-06 | |
| 1 Zoo II B02 106 04 | 429 | ref\|NP 780670.1\| | RIKEN cDNA C030014K22 gene [Mus musculus] dbj\|BAC33132.1\| unnamed protein product [Mus musculus] | 68 | 3,00E-11 | other |
| | | dbj\|BAC28491.1\| | unnamed protein product [Mus musculus] | 68 | 3,00E-11 | |
| | | ref\|XP 097622.4\| | similar to RIKEN cDNA C030014K22 gene [Homo sapiens] | 68 | 3,00E-11 | |
| | | emb\|CAF97569.1\| | unnamed protein product [Tetraodon nigroviridis] | 59 | 3,00E-08 | |
| | | emb\|CAF95856.1\| | unnamed protein product [Tetraodon nigroviridis] | 57 | 8,00E-08 | |
| | | emb\|CAF95189.1\| | unnamed protein product [Tetraodon nigroviridis] | 56 | 1,00E-07 | |
| | | gb\|AAH29924.2\| | C9orf59 protein [Homo sapiens] | 55 | 2,00E-07 | |
| | | ref\|NP 203745.1\| | chromosome 9 open reading frame 59 [Homo sapiens] gb\|AAH49388.1\| Chromosome 9 open reading frame 59 [Homo sapiens] | 55 | 2,00E-07 | |
| | | dbj\|BAB15724.1\| | FLJ00024 protein [Homo sapiens] | 55 | 2,00E-07 | |
| | | ref\|NP 780720.1\| | RIKEN cDNA A130092J06 gene [Mus musculus] dbj\|BAC37667.1\| unnamed protein product [Mus musculus] | 55 | 2,00E-07 | |
| 1 Zoo I H12 96 16 | 574 | emb\|CAF97569.1\| | unnamed protein product [Tetraodon nigroviridis] | 49 | 8,00E-05 | other |
| | | emb\|CAF95856.1\| | unnamed protein product [Tetraodon nigroviridis] | 49 | 8,00E-05 | |
| | | ref\|NP 780720.1\| | RIKEN cDNA A130092J06 gene [Mus musculus] dbj\|BAC37667.1\| unnamed protein product [Mus musculus] | 48 | 1,00E-04 | |
| | | ref\|XP 097622.4\| | similar to RIKEN cDNA C030014K22 gene [Homo sapiens] | 48 | 1,00E-04 | |
| | | ref\|NP 203745.1\| | chromosome 9 open reading frame 59 [Homo sapiens] gb\|AAH49388.1\| Chromosome 9 open reading frame 59 [Homo sapiens] | 48 | 1,00E-04 | |
| | | ref\|NP 780670.1\| | RIKEN cDNA C030014K22 gene [Mus musculus] dbj\|BAC33132.1\| unnamed protein product [Mus musculus] | 46 | 5,00E-04 | |
| | | ref\|NP 705944.1\| | epha4b; Eph receptor a4b; eph-like receptor tyrosine kinase 2 [Danio rerio] gb\|AAK54725.1\| Eph receptor EphA4b [Danio rerio] | 33 | 2,7 | |
| | | gb\|AAH75486.1\| | Unknown (protein for MGC:89323) [Xenopus tropicalis] | 33 | 4,6 | |
| | | ref\|NP 592936.1\| | GATA-type transcription factor family protein Gaf2p [Schizosaccharomyces pombe] sp\|Q10134\|FEP1_SCHPO Iron-sensing transcription factor 1 (Transcription factor gaf2) (Gaf-2) pir\|\|T38291 GATA-type | 32 | 6 | |
| | | pir\|\|T43298 | transcription factor gaf2 - fission yeast (Schizosaccharomyces pombe) gb\|AAB38022.1\| homologous to GATA-binding transcription factor [Schizosaccharomyces pombe] | 32 | 6 | |
| 1 Zoo II F05 134 11 | 414 | gb\|AAP36485.1\| | Homo sapiens sulfotransferase family, cytosolic, 1C, member 1 [synthetic construct] | 97 | 9,00E-20 | other |
| | | ref\|NP 001047.1\| | sulfotransferase family, cytosolic, 1C, member 1 isoform a; sulfotransferase 1C1 [Homo sapiens] sp\|O00338\|S1C1_HUMAN Sulfotransferase 1C1 (SULT1C#1) (ST1C2) (humSULTC2) gb\|AAC51285.1\| su | 97 | 9,00E-20 | |
| | | ref\|NP 789795.1\| | sulfotransferase family, cytosolic, 1C, member 1 isoform b; sulfotransferase 1C1 [Homo sapiens] gb\|AAF72802.1\| sulfotransferase 1C1 [Homo sapiens] gb\|AAF72803.1\| sulfotransferase 1C1 [Homo sapie | 97 | 9,00E-20 | |
| | | ref\|NP 803454.1\| | sulfotransferase, estrogen-preferring [Bos taurus] sp\|P19217\|SUOE_BOVIN Estrogen sulfotransferase (Sulfotransferase, estrogen-preferring) (ST1E1) pir\|\|S29045 estrone sulfotransferase (EC 2.8.2.4) - b | 97 | 9,00E-20 | |
| | | ref\|NP 081211.3\| | sulfotransferase family, cytosolic, 1C, member 1 [Mus musculus] sp\|Q9D939\|SUC1_MOUSE Sulfotransferase 1C1 dbj\|BAB25002.1\| unnamed protein product [Mus musculus] gb\|AAG00823.1\| sulfotransfe | 96 | 2,00E-19 | |
| | | gb\|AAH22665.1\| | Sulfotransferase family, cytosolic, 1C, member 1 [Mus musculus] | 96 | 2,00E-19 | |
| | | pir\|\|JC7283 | hydroxyarylamine sulfotransferase (EC 2.8.2.-) 2A - rat | 92 | 2,00E-18 | |
| | | sp\|Q9WUW8\|STK1 RAT | Sulfotransferase K1 (rSULT1C2) pir\|\|JC7282 hydroxyarylamine sulfotransferase (EC 2.8.2.-) 2 - rat emb\|CAB41460.1\| sulfotransferase K1 [Rattus norvegicus] | 92 | 2,00E-18 | |
| | | ref\|XP 236811.1\| | similar to Sulfotransferase K1 (rSULT1C2) [Rattus norvegicus] | 92 | 3,00E-18 | |
| | | gb\|AAF86583.1\| | sulfotransferase ST1B2 [Canis familiaris] | 91 | 4,00E-18 | |
| 1 Zoo II F06 142 12 | 221 | emb\|CAF92700.1\| | unnamed protein product [Tetraodon nigroviridis] | 62 | 2,00E-09 | other |
| | | emb\|CAB72286.1\| | EG:BACR25B3.1 [Drosophila melanogaster] | 61 | 7,00E-09 | |
| | | ref\|NP 524643.2\| | CG7981-PA [Drosophila melanogaster] gb\|AAF45786.3\| CG7981-PA [Drosophila melanogaster] | 61 | 7,00E-09 | |
| | | ref\|NP 726815.1\| | CG7981-PD [Drosophila melanogaster] emb\|CAD31650.1\| perlecan [Drosophila melanogaster] gb\|AAN09079.1\| CG7981-PD [Drosophila melanogaster] | 61 | 7,00E-09 | |
| | | ref\|NP 726814.1\| | CG7981-PE [Drosophila melanogaster] gb\|AAN09078.1\| CG7981-PE [Drosophila melanogaster] | 61 | 7,00E-09 | |
| | | ref\|NP 726816.1\| | CG7981-PB [Drosophila melanogaster] gb\|AAN09080.1\| CG7981-PB [Drosophila melanogaster] | 61 | 7,00E-09 | |
| | | ref\|NP 726813.1\| | CG7981-PC [Drosophila melanogaster] gb\|AAN09077.1\| CG7981-PC [Drosophila melanogaster] | 61 | 7,00E-09 | |
| | | emb\|CAG12052.1\| | unnamed protein product [Tetraodon nigroviridis] | 60 | 9,00E-09 | |
| | | emb\|CAG09045.1\| | unnamed protein product [Tetraodon nigroviridis] | 60 | 1,00E-08 | |
| | | ref\|XP 235261.2\| | similar to RIKEN cDNA C820005L12 [Rattus norvegicus] | 59 | 2,00E-08 | |
| 1 Zoo II H05 136 15 | 413 | ref\|NP 187850.1\| | extensin family protein [Arabidopsis thaliana] gb\|AAG51010.1\| hypothetical protein; 75534-76595 [Arabidopsis thaliana] dbj\|BAB03151.1\| unnamed protein product [Arabidopsis thaliana] | 47 | 6,00E-05 | other |
| | | ref\|XP 372890.1\| | hypothetical protein XP_377699 [Homo sapiens] | 46 | 2,00E-04 | |
| | | ref\|XP 358745.1\| | hypothetical protein XP_358745 [Mus musculus] | 46 | 2,00E-04 | |
| | | gb\|AAR36900.1\| | newborn larvae-specific serine protease SS2-1 [Trichinella spiralis] | 46 | 2,00E-04 | |
| | | gb\|AAK16520.1\| | newborn larvae-specific serine protease SS2 [Trichinella spiralis] | 46 | 2,00E-04 | |
| | | ref\|NP 898392.1\| | conserved hypothetical protein [Synechococcus sp. WH 8102] emb\|CAE08818.1\| conserved hypothetical protein [Synechococcus sp. WH 8102] | 42 | 0,003 | |
| | | ref\|NP 776304.1\| | coagulation factor V (proaccelerin, labile factor) [Bos taurus] sp\|Q28107\|FA5_BOVIN Coagulation factor V precursor (Activated protein C cofactor) pir\|\|KFBO5 coagulation factor V precursor - bovine gb\|AA | 41 | 0,005 | |
| | | ref\|NP 075004.1\| | hypothetical protein AsloCp44 [Euglena longa] sp\|P58151\|YCXC_ASTLO Hypothetical 51.9 kDa protein in rps4-rps11 intergenic region (ORF426) emb\|CAC24615.1\| hypothetical protein [Euglena longa] | 41 | 0,005 | |
| | | gb\|AAA30513.1\| | factor V | 41 | 0,005 | |
| | | ref\|NP 486602.1\| | unknown protein [Nostoc sp. PCC 7120] pir\|\|AC2126 hypothetical protein alr2562 [imported] - Nostoc sp. (strain PCC 7120) dbj\|BAB74261.1\| ORF_ID:alr2562~unknown protein [Nostoc sp. PCC 7120] | 40 | 0,008 | |

Additional file 3: Subtraction library BLAST results top 10. Engine: NCBI. Database: NR.

| Clone | bp | ID | Homology | Score | E value | category |
|---|---|---|---|---|---|---|
| 1 Zoo II G04 127 14 | 552 | ref\|NP 009614.2\| | Ubiquitin-specific protease that specifically disassembles unanchored ubiquitin chains; involved in fructose-1,6-bisphosphatase (FBPase) degradation; Ubp14p [Saccharomyces cerevisiae] | 35 | 0,65 | other* |
| | | sp\|P38237\|UBPE YEAS | Ubiquitin carboxyl-terminal hydrolase 14 (Ubiquitin thiolesterase 14) (Ubiquitin-specific processing protease 14) (Deubiquitinating enzyme 14) pir\|\|S45916 hypothetical protein YBR058c - yeast (Saccharon | 35 | 0,65 | |
| | | ref\|NP 700710.1\| | hypothetical protein [Plasmodium falciparum 3D7] gb\|AAN35434.1\| hypothetical protein [Plasmodium falciparum 3D7] | 33 | 4,2 | |
| | | ref\|NP 473088.1\| | hypothetical protein [Plasmodium falciparum 3D7] pir\|\|D71606 hypothetical protein PFB0800c - malaria parasite (Plasmodium falciparum) gb\|AAC71949.1\| hypothetical protein PFB0800c [Plasmodium falc | 32 | 7,2 | |
| | | gb\|AAA81668.1\| | ATPase subunit 6 | 32 | 7,2 | |
| | | ref\|NP 701989.1\| | hypothetical protein [Plasmodium falciparum 3D7] gb\|AAN36713.1\| hypothetical protein [Plasmodium falciparum 3D7] | 32 | 9,5 | |
| | | ref\|NP 703411.1\| | hypothetical protein [Plasmodium falciparum 3D7] emb\|CAD51431.1\| hypothetical protein [Plasmodium falciparum 3D7] | 32 | 9,5 | |
| 1 Zoo II H02 112 16 | 410 | ref\|NP 736406.1\| | Unknown [Streptococcus agalactiae NEM316] emb\|CAD47632.1\| Unknown [Streptococcus agalactiae NEM316] | 35 | 0,42 | other* |
| | | ref\|NP 485240.1\| | unknown protein [Nostoc sp. PCC 7120] pir\|\|AB1956 hypothetical protein alr1197 [imported] - Nostoc sp. (strain PCC 7120) dbj\|BAB73154.1\| ORF_ID:alr1197~unknown protein [Nostoc sp. PCC 7120] | 32 | 2,1 | |
| | | emb\|CAG85200.1\| | unnamed protein product [Debaryomyces hansenii] | 32 | 2,1 | |
| | | gb\|AAM81963.1\| | DNA polymerase I-like protein B [Trypanosoma brucei] | 32 | 2,1 | |
| | | ref\|NP 985645.1\| | AFR098Wp [Eremothecium gossypii] gb\|AAS53469.1\| AFR098Wp [Eremothecium gossypii] | 32 | 2,7 | |
| | | gb\|AAM73861.1\| | putative serine/threonine protein kinase; HcSTK [Haemonchus contortus] | 32 | 3,6 | |
| | | gb\|AAM73859.1\| | putative serine/threonine protein kinase; HcSTK [Haemonchus contortus] | 32 | 3,6 | |
| | | gb\|AAM73862.1\| | putative serine/threonine protein kinase; HcSTK [Haemonchus contortus] | 32 | 3,6 | |
| | | gb\|AAM73860.1\| | putative serine/threonine protein kinase; HcSTK [Haemonchus contortus] | 32 | 3,6 | |
| | | ref\|ZP 00159980.1\| | COG0541: Signal recognition particle GTPase [Anabaena variabilis ATCC 29413] | 32 | 3,6 | |
| 1 Zoo II H06 144 16 | 501 | sp\|Q9FH83\|WR52 ARAT | Probable WRKY transcription factor 52 (WRKY DNA-binding protein 52) (Disease resistance protein RRS1) (Resistance to Ralstonia solanacearum 1 protein) | 35 | 0,66 | other* |
| | | ref\|NP 199339.1\| | disease resistance protein (TIR-NBS-LRR class), putative [Arabidopsis thaliana] dbj\|BAB10247.1\| disease resistance protein-like [Arabidopsis thaliana] | 35 | 0,66 | |
| | | dbj\|BAC41834.1\| | putative disease resistance protein [Arabidopsis thaliana] | 35 | 0,66 | |
| | | ref\|NP 955021.1\| | PHD finger protein 16 [Mus musculus] tpe\|CAE30499.1\| TPA: Jade3 protein [Mus musculus] | 34 | 1,5 | |
| | | dbj\|BAC97901.1\| | mKIAA0215 protein [Mus musculus] | 34 | 1,5 | |
| | | ref\|XP 325400.1\| | hypothetical protein [Neurospora crassa] gb\|EAA31271.1\| hypothetical protein [Neurospora crassa] | 33 | 1,9 | |
| | | emb\|CAG80597.1\| | unnamed protein product [Yarrowia lipolytica] | 33 | 1,9 | |
| | | ref\|XP 326769.1\| | hypothetical protein [Neurospora crassa] gb\|EAA31518.1\| hypothetical protein [Neurospora crassa] emb\|CAE76117.1\| related to N-acetyltransferase [Neurospora crassa] | 33 | 2,5 | |
| | | ref\|YP 012715.1\| | conserved hypothetical protein [Listeria monocytogenes str. 4b F2365] gb\|AAT02892.1\| conserved hypothetical protein [Listeria monocytogenes str. 4b F2365] | 33 | 2,5 | |
| | | ref\|ZP 00230014.1\| | conserved hypothetical protein [Listeria monocytogenes str. 4b H7858] gb\|EAL10165.1\| conserved hypothetical protein [Listeria monocytogenes str. 4b H7858] | 33 | 2,5 | |
| 1 Zoo I A09 65 01 | 567 | ref\|NP 912801.1\| | unnamed protein product [Oryza sativa (japonica cultivar-group)] dbj\|BAA85215.1\| unnamed protein product [Oryza sativa (japonica cultivar-group)] | 37 | 0,18 | other* |
| | | emb\|CAG80731.1\| | unnamed protein product [Yarrowia lipolytica] | 37 | 0,18 | |
| | | gb\|AAD41627.1\| | dynein light chain 2 [Schistosoma japonicum] gb\|AAD41629.1\| dynein light chain 4 [Schistosoma japonicum] | 37 | 0,24 | |
| | | emb\|CAG84363.1\| | unnamed protein product [Debaryomyces hansenii] | 37 | 0,31 | |
| | | ref\|NP 194466.2\| | dynein light chain, putative [Arabidopsis thaliana] gb\|AAL36091.1\| unknown protein [Arabidopsis thaliana] gb\|AAM20341.1\| unknown protein [Arabidopsis thaliana] | 36 | 0,53 | |
| | | ref\|NP 188233.1\| | dynein light chain, putative [Arabidopsis thaliana] dbj\|BAB02678.1\| unnamed protein product [Arabidopsis thaliana] gb\|AAM63499.1\| putative dynein light chain protein [Arabidopsis thaliana] | 36 | 0,53 | |
| | | ref\|NP 594368.1\| | dynein light chain [Schizosaccharomyces pombe] sp\|Q9UR05\|DYL1_SCHPO Dynein light chain 1, cytoplasmic pir\|\|T39205 dynein light chain - fission yeast (Schizosaccharomyces pombe) emb\|CAB5415 | 36 | 0,53 | |
| | | pir\|\|T05760 | hypothetical protein M4I22.170 - Arabidopsis thaliana emb\|CAA19730.1\| putative protein [Arabidopsis thaliana] emb\|CAB79591.1\| putative protein [Arabidopsis thaliana] | 36 | 0,53 | |
| | | ref\|NP 984289.1\| | ADR193Wp [Eremothecium gossypii] gb\|AAS52113.1\| ADR193Wp [Eremothecium gossypii] | 35 | 0,69 | |
| | | ref\|NP 175635.1\| | dynein light chain type 1 family protein [Arabidopsis thaliana] pir\|\|E96562 unknown protein, 73838-74229 [imported] - Arabidopsis thaliana gb\|AAF29412.1\| dynein light chain, putative [Arabidopsis thaliana | 35 | 0,69 | |
| 1 Zoo I A11 81 01 | 279 | dbj\|BAD19631.1\| | hypothetical protein [Oryza sativa (japonica cultivar-group)] | 35 | 0,39 | other* |
| | | emb\|CAA11051.1\| | immunoglobulin heavy chain [Acipenser baerii] | 32 | 3,3 | |
| | | emb\|CAE69531.1\| | Hypothetical protein CBG15740 [Caenorhabditis briggsae] | 31 | 5,6 | |
| | | emb\|CAA73713.1\| | immunoglobulin heavy chain [Acipenser baerii] | 31 | 7,3 | |
| | | pir\|\|T45134 | hypothetical protein [imported] - Microbacterium ammoniaphilum (fragment) emb\|CAA55649.1\| unknown [Microbacterium ammoniaphilum] | 31 | 7,3 | |
| | | emb\|CAA11014.1\| | immunoglobulin heavy chain [Acipenser baerii] | 30 | 9,6 | |
| 1 Zoo I A12 89 02 | 317 | ref\|NP 593798.1\| | WD repeat protein, possible mitotic checkpoint [Schizosaccharomyces pombe] pir\|\|T38301 probable mitotic checkpoint WD repeat protein - fission yeast (Schizosaccharomyces pombe) emb\|CAB16243.1\| | 35 | 0,28 | other* |
| | | ref\|XP 341238.1\| | similar to toll-associated serine protease [Rattus norvegicus] | 34 | 0,82 | |
| | | ref\|NP 037078.1\| | extracellular matrix protein 2 [Rattus norvegicus] sp\|P24054\|SPL1_RAT SPARC-like protein 1 precursor (Matrix glycoprotein Sc1) pir\|\|GERTX1 matrix glycoprotein SC1 precursor - rat gb\|AAA68708.1\| SC | 34 | 0,82 | |
| | | ref\|XP 357292.1\| | similar to 60S ribosomal protein L23a [Mus musculus] | 32 | 4,1 | |
| | | ref\|NP 652526.1\| | CG3576-PB [Drosophila melanogaster] ref\|NP_727075.1\| CG3576-PA [Drosophila melanogaster] gb\|AAL28803.1\| LD18904p [Drosophila melanogaster] gb\|AAL57756.1\| longevity protein [Drosophila mela | 32 | 4,1 | |
| | | ref\|XP 234397.2\| | similar to 60S ribosomal protein L23a [Rattus norvegicus] | 31 | 5,3 | |
| | | ref\|XP 195305.2\| | similar to 60S ribosomal protein L23a [Mus musculus] | 31 | 5,3 | |
| | | ref\|NP 116803.1\| | MS115, putative beta-agarase precursor [Microscilla sp. PRE1] gb\|AAK62837.1\| MS115, putative beta-agarase precursor [Microscilla sp. PRE1] | 31 | 5,3 | |
| | | emb\|CAC37287.1\| | C367G8.3 (novel protein similar to RPL23A (60S ribosomal protein L23A)) [Homo sapiens] gb\|AAK61228.1\| 60S ribosomal protein L23A like [Homo sapiens] | 31 | 7 | |
| | | ref\|XP 312609.1\| | ENSANGP00000001289 [Anopheles gambiae] gb\|EAA08227.1\| ENSANGP00000001289 [Anopheles gambiae str. PEST] | 31 | 7 | |

Additional file 3: Subtraction library BLAST results top 10. Engine: NCBI. Database: NR.

| Clone | bp | ID | Homology | Score | E value | category |
|---|---|---|---|---|---|---|
| 1_Zoo_I_B04_26_04 | 454 | gb\|EAL23322.1\| | hypothetical protein CNBA4380 [Cryptococcus neoformans var. neoformans B-3501A] | 36 | 0,18 | other* |
| | | ref\|NP_077341.1\| | 5-hydroxytryptamine (serotonin) receptor 6 [Rattus norvegicus] sp\|P31388\|5H6_RAT 5-hydroxytryptamine 6 receptor (5-HT-6) (Serotonin receptor 6) (ST-B17) pir\|\|JN0591 serotonin receptor 6 - rat gb\|AAA | 33 | 1,5 | |
| | | gb\|EAL19732.1\| | hypothetical protein CNBG3600 [Cryptococcus neoformans var. neoformans B-3501A] | 32 | 2 | |
| | | gb\|EAA46797.1\| | hypothetical protein MG10491.4 [Magnaporthe grisea 70-15] | 32 | 2,6 | |
| | | ref\|NP_955369.1\| | similar to RIKEN cDNA 1810059G22 [Homo sapiens] gb\|AAH51355.1\| Similar to RIKEN cDNA 1810059G22 [Homo sapiens] | 32 | 2,6 | |
| | | ref\|XP_134707.2\| | RIKEN cDNA 4921509F24 [Mus musculus] | 32 | 2,6 | |
| | | dbj\|BAB29584.1\| | unnamed protein product [Mus musculus] | 32 | 2,6 | |
| | | emb\|CAG06428.1\| | unnamed protein product [Tetraodon nigroviridis] | 32 | 3,4 | |
| | | dbj\|BAB14975.1\| | unnamed protein product [Homo sapiens] | 32 | 3,4 | |
| | | ref\|ZP_00304902.1\| | COG0318: Acyl-CoA synthetases (AMP-forming)/AMP-acid ligases II [Novosphingobium aromaticivorans DSM 12444] | 32 | 3,4 | |
| 1_Zoo_I_B07_50_03 | 320 | ref\|XP_320743.1\| | ENSANGP00000020029 [Anopheles gambiae] gb\|EAA00362.1\| ENSANGP00000020029 [Anopheles gambiae str. PEST] | 34 | 0,82 | other* |
| | | emb\|CAE74414.1\| | Hypothetical protein CBG22146 [Caenorhabditis briggsae] | 33 | 1,1 | |
| | | gb\|EAA41880.1\| | GLP_158_68817_64606 [Giardia lamblia ATCC 50803] | 33 | 1,8 | |
| | | ref\|NP_375057.1\| | enterotoxin P [Staphylococcus aureus subsp. aureus N315] ref\|NP_835571.1\| enterotoxin P [Staphylococcus phage phiN315] pir\|\|C89984 enterotoxin P [imported] - Staphylococcus aureus (strain N315) d | 32 | 2,4 | |
| | | prf\|\|1704203A | enterotoxin A | 32 | 2,4 | |
| | | dbj\|BAC87286.1\| | unnamed protein product [Homo sapiens] | 32 | 3,1 | |
| | | gb\|EAA42869.1\| | GLP_574_135852_134011 [Giardia lamblia ATCC 50803] | 31 | 5,3 | |
| | | ref\|NP_293990.1\| | conserved hypothetical protein [Deinococcus radiodurans R1] pir\|\|D75540 conserved hypothetical protein - Deinococcus radiodurans (strain R1) gb\|AAF09850.1\| conserved hypothetical protein [Deinococc | 31 | 5,3 | |
| | | sp\|Q9XY92\|SPEE_DICD | Spermidine synthase (Putrescine aminopropyltransferase) (SPDSY) gb\|AAD32851.1\| spermidine synthase [Dictyostelium discoideum] | 31 | 6,9 | |
| | | sp\|P79703\|JUNB_CYPC | Transcription factor jun-B gb\|AAB39939.1\| junB transcription factor [Cyprinus carpio] | 31 | 6,9 | |
| 1_Zoo_I_B08_58_04 | 315 | emb\|CAE66995.1\| | Hypothetical protein CBG12393 [Caenorhabditis briggsae] | 37 | 0,13 | other* |
| | | ref\|NP_001002186.1\| | zgc:91880 [Danio rerio] gb\|AAH74038.1\| Unknown (protein for MGC:91880) [Danio rerio] | 36 | 0,22 | |
| | | gb\|AAM09300.1\| | CC chemokine with stalk CK2 [Oncorhynchus mykiss] | 35 | 0,37 | |
| | | emb\|CAG02436.1\| | unnamed protein product [Tetraodon nigroviridis] | 34 | 0,63 | |
| | | emb\|CAG13131.1\| | unnamed protein product [Tetraodon nigroviridis] | 34 | 0,83 | |
| | | ref\|NP_694438.1\| | putative replication protein E1 [Reindeer papillomavirus] gb\|AAN09917.1\| putative replication protein E1 [reindeer papillomavirus] | 33 | 1,1 | |
| | | gb\|EAA15185.1\| | nucleotide-binding protein [Plasmodium yoelii yoelii] | 33 | 1,1 | |
| | | pir\|\|T22544 | hypothetical protein F53B6.1 - Caenorhabditis elegans | 33 | 1,4 | |
| | | ref\|NP_492404.1\| | tetraspanin family member (tsp-15) [Caenorhabditis elegans] emb\|CAB03120.2\| C. elegans TSP-15 protein (corresponding sequence F53B6.1) [Caenorhabditis elegans] | 33 | 1,4 | |
| | | ref\|XP_314330.1\| | ENSANGP00000012724 [Anopheles gambiae] gb\|EAA09730.1\| ENSANGP00000012724 [Anopheles gambiae str. PEST] | 33 | 1,4 | |
| 1_Zoo_I_C08_59_06 | 452 | dbj\|BAD25060.1\| | putative zinc-binding protein [Oryza sativa (japonica cultivar-group)] | 35 | 0,4 | other* |
| | | ref\|NP_990301.1\| | cation-independent mannose-6-phosphate receptor [Gallus gallus] pir\|\|I50726 cation-independent mannose-6-phosphate receptor - chicken gb\|AAC59718.1\| cation-independent mannose-6-phosphate rec | 33 | 1,2 | |
| | | ref\|XP_452462.1\| | unnamed protein product [Kluyveromyces lactis] sp\|O59863\|PLB_KLULA Lysophospholipase precursor (Phospholipase B) (KlPLB) dbj\|BAA28619.1\| phospholipase B [Kluyveromyces lactis] emb\|CAH013 | 33 | 1,5 | |
| | | ref\|XP_396698.1\| | similar to CG9281-PB [Apis mellifera] | 33 | 1,5 | |
| | | gb\|AAK95906.1\| | polymerase [Influenza B virus (B/Shangdong/7/97)] | 31 | 4,4 | |
| | | gb\|AAM19148.1\| | envelope glycoprotein [Crimean-Congo hemorrhagic fever virus] | 31 | 4,4 | |
| | | gb\|AAK94481.1\| | envelope glycoprotein [Crimean-Congo hemorrhagic fever virus] | 31 | 4,4 | |
| | | gb\|AAK94477.1\| | envelope glycoprotein [Crimean-Congo hemorrhagic fever virus] gb\|AAM19152.1\| envelope glycoprotein [Crimean-Congo hemorrhagic fever virus] | 31 | 4,4 | |
| | | gb\|AAK94480.1\| | envelope glycoprotein [Crimean-Congo hemorrhagic fever virus] gb\|AAM19150.1\| envelope glycoprotein [Crimean-Congo hemorrhagic fever virus] | 31 | 4,4 | |
| | | gb\|AAF06861.1\| | polymerase [Influenza B virus (B/Beijing/184/93)] | 31 | 4,4 | |
| 1_Zoo_I_D11_84_07 | 552 | emb\|CAG83560.1\| | unnamed protein product [Yarrowia lipolytica] | 36 | 0,5 | other* |
| | | emb\|CAF89910.1\| | unnamed protein product [Tetraodon nigroviridis] | 32 | 5,5 | |
| | | emb\|CAF92278.1\| | unnamed protein product [Tetraodon nigroviridis] | 32 | 5,5 | |
| | | ref\|NP_281993.1\| | putative integral membrane protein [Campylobacter jejuni subsp. jejuni NCTC 11168] pir\|\|H81355 probable integral membrane protein Cj0832c [imported] - Campylobacter jejuni (strain NCTC 11168) emb | 32 | 7,2 | |
| | | ref\|XP_396525.1\| | similar to Dosage compensation regulator (Male-less protein) (No action potential protein) [Apis mellifera] | 32 | 9,5 | |
| 1_Zoo_I_E07_53_09 | 576 | ref\|XP_223090.2\| | similar to Kif21b [Rattus norvegicus] | 37 | 0,32 | other* |
| | | ref\|XP_371332.1\| | kinesin family member 21B [Homo sapiens] | 35 | 1,2 | |
| | | ref\|NP_505184.1\| | reverse transcriptase RNase H-like protein family member (5I956) [Caenorhabditis elegans] pir\|\|T32228 hypothetical protein T23B12.9 - Caenorhabditis elegans gb\|AAB69941.1\| Hypothetical protein T23B | 34 | 2,1 | |
| | | dbj\|BAB01254.1\| | centromere protein [Arabidopsis thaliana] | 33 | 2,7 | |
| | | ref\|NP_188918.1\| | kinase interacting family protein [Arabidopsis thaliana] | 33 | 2,7 | |
| | | gb\|EAK94874.1\| | hypothetical protein CaO19.9845 [Candida albicans SC5314] | 33 | 3,6 | |
| | | gb\|EAK94815.1\| | hypothetical protein CaO19.2309 [Candida albicans SC5314] | 33 | 3,6 | |
| | | gb\|EAL18878.1\| | hypothetical protein CNBI1390 [Cryptococcus neoformans var. neoformans B-3501A] | 33 | 4,7 | |
| | | ref\|NP_473182.1\| | hypothetical protein, conserved [Plasmodium falciparum 3D7] emb\|CAB39005.1\| hypothetical protein, conserved [Plasmodium falciparum 3D7] | 32 | 8 | |
| | | ref\|NP_296903.1\| | conserved hypothetical protein [Chlamydia muridarum] pir\|\|C81693 conserved hypothetical protein TC0526 [imported] - Chlamydia muridarum (strain Nigg) gb\|AAF39368.1\| conserved hypothetical protein | 32 | 8 | |
| 1_Zoo_I_F08_62_12 | 398 | ref\|XP_355968.1\| | RIKEN cDNA 8030491N06 [Mus musculus] | 33 | 0,96 | other* |
| | | ref\|XP_219126.2\| | similar to FLJ00251 protein [Rattus norvegicus] | 32 | 2,8 | |
| | | ref\|NP_393607.1\| | amylopullulanase related protein [Thermoplasma acidophilum DSM 1728] emb\|CAC11276.1\| amylopullulanase related protein [Thermoplasma acidophilum] | 31 | 6,2 | |
| | | ref\|NP_702453.1\| | hypothetical protein, conserved [Plasmodium falciparum 3D7] gb\|AAN37177.1\| hypothetical protein, conserved [Plasmodium falciparum 3D7] | 30 | 8,1 | |

## Additional file 3: Subtraction library BLAST results top 10. Engine: NCBI. Database: NR.

| Clone | bp | ID | Homology | Score | E value | category |
|---|---|---|---|---|---|---|
| 1_Zoo_I_G06_47_14 | 653 | ref\|NP_325899.1\| | conserved hypothetical protein [Mycoplasma pulmonis UAB CTIP] pir\|\|D90520 conserved hypothetical protein MYPU_0680 [imported] - Mycoplasma pulmonis (strain UAB CTIP) emb\|CAC13241.1\| conser | 36 | 0,73 | other* |
| | | gb\|EAK87323.1\| | hypothetical protein containing a signal peptide and low complexity repeats, member of cryptosporidium SKSR gene family, telomeric localized gene [Cryptosporidium parvum] | 35 | 0,95 | |
| | | gb\|AAF04793.1\| | structural polyprotein [Eastern equine encephalitis virus] | 34 | 2,1 | |
| | | ref\|ZP_00040252.1\| | COG3210: Large exoproteins involved in heme utilization or adhesion [Xylella fastidiosa Ann-1] | 33 | 3,6 | |
| | | ref\|NP_008734.1\|ND4_15 | NADH dehydrogenase subunit 4 [Smithornis sharpei] pir\|\|T11189 NADH2 dehydrogenase (ubiquinone) (EC 1.6.5.3) chain 4 - Smithornis sharpei mitochondrion gb\|AAD32523.1\| NADH dehydrogenase sub | 33 | 6,2 | |
| | | ref\|NP_727328.1\| | CG32704-PA [Drosophila melanogaster] gb\|AAF46470.2\| CG32704-PA [Drosophila melanogaster] | 32 | 8,1 | |
| | | sp\|P08768\|POLS_EEEV | Structural polyprotein (P130) [Contains: Coat protein C (Capsid protein C); Spike glycoprotein E3; Spike glycoprotein E2; 6 kDa peptide; Spike glycoprotein E1] pir\|\|VHWVEE structural polyprotein - easter | 32 | 8,1 | |
| | | ref\|ZP_00038092.1\| | COG3210: Large exoproteins involved in heme utilization or adhesion [Xylella fastidiosa Dixon] | 32 | 8,1 | |
| 1_Zoo_I_G09_71_13 | 577 | ref\|XP_223090.2\| | similar to Kif21b [Rattus norvegicus] | 35 | 0,94 | other* |
| | | ref\|NP_505184.1\| | reverse transcriptase RNase H-like protein family member (5I956) [Caenorhabditis elegans] pir\|\|T32228 hypothetical protein T23B12.9 - Caenorhabditis elegans gb\|AAB69941.1\| Hypothetical protein T23B | 34 | 2,1 | |
| | | ref\|ZP_00236583.1\| | conserved hypothetical protein protein [Bacillus cereus G9241] gb\|EAL15859.1\| conserved hypothetical protein protein [Bacillus cereus G9241] | 33 | 3,6 | |
| | | ref\|XP_371332.1\| | kinesin family member 21B [Homo sapiens] | 33 | 3,6 | |
| | | gb\|EAK94874.1\| | hypothetical protein CaO19.9845 [Candida albicans SC5314] | 33 | 3,6 | |
| | | gb\|EAK94815.1\| | hypothetical protein CaO19.2309 [Candida albicans SC5314] | 33 | 3,6 | |
| | | emb\|CAB62997.1\| | dJ180M12.1 (KIAA0645) [Homo sapiens] | 32 | 8 | |
| | | ref\|NP_978201.1\| | conserved hypothetical protein [Bacillus cereus ATCC 10987] gb\|AAS40809.1\| conserved hypothetical protein [Bacillus cereus ATCC 10987] | 32 | 8 | |
| | | dbj\|BAA31620.2\| | KIAA0645 protein [Homo sapiens] | 32 | 8 | |
| | | ref\|XP_376007.1\| | KIAA0645 gene product [Homo sapiens] sp\|O75140\|DEP5_HUMAN DEP domain containing protein 5 pir\|\|T00376 KIAA0645 protein - human | 32 | 8 | |
| 1_Zoo_I_H08_64_16 | 511 | ref\|NP_404035.1\| | conserved hypothetical protein [Yersinia pestis] ref\|NP_671090.1\| hypothetical protein [Yersinia pestis KIM] ref\|NP_995064.1\| conserved hypothetical protein [Yersinia pestis biovar Medievalis str. 91001] pir\|\|AC0 | 35 | 0,69 | other* |
| | | emb\|CAD61269.1\| | SI:zC214P16.5 (novel protein similar to human RAB3 GTPase-activating protein (RAB3GAP)) [Danio rerio] | 35 | 0,9 | |
| | | ref\|ZP_00129398.1\| | hypothetical protein [Desulfovibrio desulfuricans G20] | 34 | 1,2 | |
| | | ref\|ZP_00295462.1\| | COG1357: Uncharacterized low-complexity proteins [Methanosarcina barkeri str. fusaro] | 33 | 2 | |
| | | ref\|XP_394083.1\| | similar to ENSANGP00000011733 [Apis mellifera] | 33 | 2 | |
| | | ref\|XP_403694.1\| | hypothetical protein UM06079.1 [Ustilago maydis 521] gb\|EAK86902.1\| hypothetical protein UM06079.1 [Ustilago maydis 521] | 33 | 3,4 | |
| | | gb\|AAC02666.1\| | polyprotein [Arabidopsis thaliana] | 32 | 4,5 | |
| | | gb\|AAC02664.1\| | polyprotein [Arabidopsis thaliana] | 32 | 4,5 | |
| | | gb\|AAC02669.1\| | polyprotein [Arabidopsis thaliana] | 32 | 4,5 | |
| | | ref\|ZP_00220227.1\| | COG1674: DNA segregation ATPase FtsK/SpoIIIE and related proteins [Burkholderia cepacia R1808] | 32 | 4,5 | |
| 1_Zoo_I_H11_88_15 | 549 | emb\|CAG13731.1\| | unnamed protein product [Tetraodon nigroviridis] | 36 | 0,49 | other* |
| | | ref\|NP_774312.1\| | bll7672 [Bradyrhizobium japonicum USDA 110] dbj\|BAC52937.1\| bll7672 [Bradyrhizobium japonicum USDA 110] | 35 | 0,84 | |
| | | ref\|NP_648240.3\| | CG32355-PA [Drosophila melanogaster] gb\|AAL28576.1\| HL05638p [Drosophila melanogaster] gb\|AAN12010.2\| CG32355-PA [Drosophila melanogaster] | 34 | 1,9 | |
| | | gb\|AAM29365.1\| | HL08076p [Drosophila melanogaster] | 34 | 1,9 | |
| | | ref\|ZP_00039879.1\| | COG0557: Exoribonuclease R [Xylella fastidiosa Dixon] | 33 | 2,4 | |
| | | ref\|NP_643105.1\| | conserved hypothetical protein [Xanthomonas axonopodis pv. citri str. 306] gb\|AAM37641.1\| conserved hypothetical protein [Xanthomonas axonopodis pv. citri str. 306] | 33 | 3,2 | |
| | | ref\|NP_779039.1\| | truncated ribonuclease R [Xylella fastidiosa Temecula1] gb\|AAO28688.1\| truncated ribonuclease R [Xylella fastidiosa Temecula1] | 33 | 3,2 | |
| | | gb\|AAB96954.1\| | unknown [Mycobacterium bovis BCG] | 33 | 3,2 | |
| | | ref\|ZP_00041828.1\| | COG0557: Exoribonuclease R [Xylella fastidiosa Ann-1] | 33 | 3,2 | |
| | | ref\|NP_049591.1\| | haem lyase [Tetrahymena pyriformis] gb\|AAD41936.1\| haem lyase [Tetrahymena pyriformis] | 33 | 4,2 | |
| 1_Zoo_II_C02_107_06 | 407 | gb\|EAL23390.1\| | hypothetical protein CNBA0400 [Cryptococcus neoformans var. neoformans B-3501A] | 36 | 0,15 | other* |
| | | ref\|NP_003610.1\| | neurotrypsin precursor; brain-specific serine protease 3; leydin [Homo sapiens] sp\|P56730\|NETR_HUMAN Neurotrypsin precursor (Motopsin) (Leydin) emb\|CAA04816.1\| neurotrypsin [Homo sapiens] | 36 | 0,19 | |
| | | ref\|ZP_00185615.2\| | hypothetical protein Rxyl023183 [Rubrobacter xylanophilus DSM 9941] | 34 | 0,56 | |
| | | gb\|AAC26795.1\| | M protein [Streptococcus pyogenes] | 33 | 0,95 | |
| | | ref\|ZP_00226586.1\| | COG0569: K+ transport systems, NAD-binding component [Kineococcus radiotolerans SRS30216] | 33 | 0,95 | |
| | | gb\|AAN46661.1\| | M protein precursor [Streptococcus pyogenes] | 33 | 1,2 | |
| | | ref\|ZP_00228209.1\| | hypothetical protein Krad06001650 [Kineococcus radiotolerans SRS30216] | 33 | 1,6 | |
| | | ref\|NP_701981.1\| | hypothetical protein [Plasmodium falciparum 3D7] gb\|AAN36705.1\| hypothetical protein [Plasmodium falciparum 3D7] | 33 | 1,6 | |
| | | gb\|AAP73846.1\| | hypothetical protein [Oryza sativa (japonica cultivar-group)] | 33 | 1,6 | |
| | | gb\|AAH43850.1\| | MGC53673 protein [Xenopus laevis] | 32 | 2,1 | |
| 1_Zoo_II_F03_118_11 | 511 | emb\|CAG13047.1\| | unnamed protein product [Tetraodon nigroviridis] | 37 | 0,24 | other* |
| | | gb\|AAT41732.1\| | At5g27860 [Arabidopsis thaliana] | 35 | 0,9 | |
| | | dbj\|BAC03505.1\| | unnamed protein product [Homo sapiens] | 33 | 2,6 | |
| | | ref\|XP_410487.1\| | hypothetical protein AN6350.2 [Aspergillus nidulans FGSC A4] gb\|EAA58734.1\| hypothetical protein AN6350.2 [Aspergillus nidulans FGSC A4] | 33 | 3,4 | |
| | | ref\|XP_410086.1\| | hypothetical protein AN5949.2 [Aspergillus nidulans FGSC A4] gb\|EAA57812.1\| hypothetical protein AN5949.2 [Aspergillus nidulans FGSC A4] | 32 | 5,8 | |
| | | dbj\|BAA95483.1\| | Kielin [Xenopus laevis] | 32 | 7,6 | |
| | | emb\|CAG00254.1\| | unnamed protein product [Tetraodon nigroviridis] | 32 | 7,6 | |
| | | emb\|CAF87357.1\| | unnamed protein product [Tetraodon nigroviridis] | 32 | 7,6 | |
| | | ref\|XP_327045.1\| | predicted protein [Neurospora crassa] emb\|CAD70387.1\| hypothetical protein [Neurospora crassa] gb\|EAA34295.1\| predicted protein [Neurospora crassa] | 32 | 7,6 | |
| | | gb\|AAN11303.1\| | vitellogenin VTG [Penaeus semisulcatus] | 31 | 9,9 | |
| 80 clones with “no match” BLAST results are not listed. | | | | | | |

* clones from the category “other” with E values between 1x10-2 and 0,94
